# From Farm to Retail: Decoding the Elemental Landscape of Milk and Dairy Products Across Organic and Conventional Production Systems Using ICP–MS

**DOI:** 10.3390/toxics14020124

**Published:** 2026-01-28

**Authors:** Ovidiu Iliuță Marcus, Alexandra Tabaran, Oana Lucia Crișan Reget, Sorin Daniel Dan, Luciana-Catalina Panait, Caroline-Maria Lăcătuș, Maria Popescu, Andrei Răzvan Codea, Robert Cristian Purdoiu, Radu Lăcătuș, Ioan Valentin Petrescu-Mag, Alexandru Nicolescu, Florin-Dumitru Bora

**Affiliations:** 1 Department of Clinical Sciences, Faculty of Veterinary Medicine, University of Agricultural Sciences and Veterinary Medicine of Cluj-Napoca, 3–5 Mănăștur Street, 400372 Cluj-Napoca, Romania; ovidiu-iliuta.marcus@student.usamvcluj.ro (O.I.M.); radu.lacatus@usamvcluj.ro (R.L.); 2 Department of Animal Production and Food Safety, Faculty of Veterinary Medicine, University of Agricultural Sciences and Veterinary Medicine of Cluj-Napoca, 400684 Cluj-Napoca, Romania; alexandra.lapusan@usamvcluj.ro (A.T.); oana.reget@usamvcluj.ro (O.L.C.R.); sorindan@usamvcluj.ro (S.D.D.); luciana.rus@usamvcluj.ro (L.-C.P.); carolinelacatus@yahoo.com (C.-M.L.); 3 Equine Clinic, Faculty of Veterinary Medicine, University of Agricultural Sciences and Veterinary Medicine (UASVM) Cluj-Napoca, 3–5 Mănăștur Street, 400372 Cluj-Napoca, Romania; maria.popescu@usamvcluj.ro; 4 Internal Medicine Department, University of Agricultural Sciences and Veterinary Medicine (UASVM) Cluj-Napoca, 3–5 Mănăștur Street, 400372 Cluj-Napoca, Romania; razvan.codea@usamvcluj.ro; 5 Department of Veterinary Medical Imaging, University of Agricultural Sciences and Veterinary Medicine (UASVM) Cluj-Napoca, 3–5 Mănăștur Street, 400372 Cluj-Napoca, Romania; 6 Department of Environmental Engineering and Protection, Faculty of Agriculture, University of Agricultural Sciences and Veterinary Medicine (UASVM) Cluj-Napoca, 3–5 Mănăștur Street, 400372 Cluj-Napoca, Romania; ioan.mag@usamvcluj.ro; 7 Laboratory of Chromatography, Advanced Horticultural Research Institute of Transylvania, Faculty of Horticulture and Business for Rural Development, University of Agricultural Sciences and Veterinary Medicine of Cluj-Napoca, 400372 Cluj-Napoca, Romania; 8 Viticulture and Oenology Department, Faculty of Horticulture and Business in Rural Development, University of Agricultural Sciences and Veterinary Medicine of Cluj-Napoca, 3–5 Mănăștur Street, 400372 Cluj-Napoca, Romania

**Keywords:** dairy products, trace metals, multi-elemental analysis, ICP–MS, organic and conventional production systems, dairy supply chain, heavy metal contamination, buffalo milk, donkey milk, goat milk

## Abstract

The presence of trace and toxic elements in milk and dairy products is an important food safety issue, as contamination can occur along the dairy supply chain and may be influenced by animal species, production system, and processing conditions. This study aimed to investigate and compare the multi-elemental composition of milk and selected dairy products obtained from organic, conventional, and commercial production systems in north-western Romania. A total of 307 samples, including raw milk from different animal species (cow, goat, buffalo, donkey) as well as yogurt, cheese, and mozzarella, were collected from farms and retail outlets. Samples were subjected to standardized microwave-assisted acid digestion and analyzed for toxic and essential elements (Pb, Cd, Hg, As, Cr, Ni, Al, Sn, Cu, and Zn) using inductively coupled plasma mass spectrometry (ICP–MS), with quality assurance ensured through certified reference materials and proficiency testing. The results indicated low concentrations of toxic metals across all dairy matrices, with Pb ranging from 0.0047 to 0.0117 mg/kg, Cd from 0.0008 to 0.0011 mg/kg, and As from 0.0007 to 0.0664 mg/kg, depending on animal species and production system. Mercury was consistently below the limit of detection in all datasets (LCD = 100%). Essential and transition elements were systematically quantified, occurring within expected ranges (Al: 0.021–0.264 mg/kg; Cu: 0.078–0.270 mg/kg; Zn: 3.245–7.963 mg/kg; Sn ≈ 0.0030–0.0035 mg/kg). All toxic element concentrations were below the maximum limits established by European Union legislation. Variations in elemental profiles were observed between animal species and production systems, with organic cow milk showing the most homogeneous composition. All toxic element concentrations were below the maximum limits established by European Union legislation. Overall, the findings confirm the safety of the analyzed dairy products and emphasize the relevance of multi-elemental monitoring as a practical tool for dairy supply chain surveillance and risk assessment.

## 1. Introduction

With over 80% of the global population regularly consuming dairy products, their relevance goes beyond the food industry, with additional social and ecological implications [1]. In human nutrition, milk and dairy products represent a crucial source of macronutrients (e.g., proteins, amino acids, lipids, and lactose), which are needed for essential physiological processes, with additional minerals (e.g., Ca, Mg, Na, P, and K) also being present. Moreover, trace essential elements (e.g., Fe, Zn, Cu, and Se) also contribute to this nutritional profile, presenting biochemical functions as enzymatic cofactors and immune regulators [2,3].

Although the secretion of trace elements is meaningful for the nutritional quality of milk, the same phenomenon can be observed for elements presenting toxicity (such as Cd, Pb, Hg, Ni, Mn, or As), which can become concentrated in milk and dairy due to exposure to environmental contamination, feedstuff, and pollution [4,5,6]. Due to this aspect, the increase in the demands of the dairy sector is a major factor with potential serious effects on public health [7]. As global consumption continues to rise, public health authorities are making efforts to ensure the safety of these products [6]. One of the most recent regulatory updates in the European Union is the Commission Regulation (EU) 2023/915 of the European Union (EU) (from April 2023), which establishes the lower maximum levels for food contaminants [8,9].

The complex profile of milk is based on the dynamic accumulation of its components, where several factors were identified as major, namely the animal species used for collection, the production systems used, and the applied technological processing. While cow milk (especially from *Bos taurus*, Bovidae) dominates the global market, there is a growing interest in alternative forms, including buffalo (*Bubalus bubalis*, Bovidae), goat (*Capra hircus*, Bovidae), and donkey (*Equus asinus*, Equidae) milk, which show unique nutritional profiles as well as being used in specialized products (such as Mozzarella cheese or ghee) [10,11,12,13]. Despite this, information regarding trace elements with potential toxicity in buffalo, goat, and donkey milk is still scarce, and research is still ongoing [14].

Within the food sector, the difference between “organic” and “conventional” production systems is another key factor, being especially important for consumer perception and choice [15], where the so-called organic dairy products are perceived as environmentally friendly, sustainable, and with lower risks of contamination in comparison to the conventional ones [16,17,18]. However, it remains unclear whether organic production systems can eliminate the effects of the ubiquitous environmental pollution, which is present both in soil and groundwater [19,20]. What is more, recent data also suggest that there might be no significant difference between organic and conventional milk in terms of their macromineral and trace element content [21]. At the same time, the content in macronutrients (such as fatty acids and some vitamins) can be differentiated against these two classes of products [18].

Furthermore, there is a serious research gap regarding the fate of trace elements during the processing of raw milk into its commercial derivatives, such as during fermentation (to obtain yogurt) and coagulation (to obtain cheese). In this case, it remains unclear how toxic elements are partitioned and whether they can cause serious concentration effects, especially during every stage of dairy production [22,23]. From an analytical perspective, high-sensitivity techniques such as inductively coupled plasma mass spectrometry (ICP-MS) are used in the quantification of such trace toxic metals within milk and dairy products, with detection limits as low as µg/kg [24].

This study aimed to characterize the multi-elemental composition of milk and selected dairy products from different animal species, production systems, and processing stages using ICP–MS, and to evaluate their compliance with European Union regulatory limits. Specifically, we investigated whether essential and toxic metal profiles vary with (i) animal species (cow, buffalo, goat, donkey), (ii) processing sequences (raw milk, yogurt, and cheese), and (iii) production systems (organic, conventional, and commercial).

The novelty of this work lies in its large and structured sample set (307 samples), the simultaneous comparison of four dairy species within a single regional production context, and the integrated supply-chain perspective spanning raw milk to processed products. This combined approach enables a comprehensive assessment of species-, processing-, and production-system–driven effects on elemental profiles and supports food safety monitoring and traceability within the dairy supply chain.

## 2. Materials and Methods

### 2.1. Study Site and Sampling Design

Sampling was conducted exclusively in October 2025 in north-western Romania, at primary production sites and retail outlets located in Sălaj County (Crișeni) and Satu Mare County (Certeze and Moiseni). These locations were selected to represent typical dairy production systems in a predominantly rural region of north-western Romania. This sampling window was selected to ensure uniformity of climatic and feeding conditions across all production systems. The study integrated three supply chains—organic farm, farm-origin conventional, and commercial retail—to capture the main dairy matrices circulating in the region. All samples were assigned internal identification codes according to production system, animal species, matrix, and batch, with complete metadata reported in the Supplementary Material (Tables S1–S7).

The dataset comprises 307 dairy product samples distributed across multiple matrices (milk, yogurt, cheese, and mozzarella) and two counties, as detailed in Tables S1–S7. The organic series from Sălaj County includes *n* = 90 samples collected on 5 October 2025: cow milk *n* = 30 (S1-MLK-001–030; Table S1), cow yogurt *n* = 30 (S1-YGT-031–060; Table S2), and cow cheese *n* = 30 (S1-CHS-061–090; Table S3). The farm-origin conventional series from Satu Mare County comprises *n* = 100 samples collected on 24 October 2025: goat milk *n* = 30 (S2-GOA-001C–030C; Table S4), buffalo milk *n* = 40 (S2-BUF-001B–040B; Table S5), and donkey milk *n* = 30 (S2-DNK-001M–030M; Table S6). The commercial/retail component collected on 30 October 2025 includes *n* = 60 conventional cow products purchased from retail (milk LM1–LM20; yogurt IM1–IM20; cheese BM1–BM20) and *n* = 57 buffalo farm-origin products from the Certeze area (buffalo mozzarella *n* = 30, BB1–BB30; buffalo yogurt *n* = 27, IB1–IB27), all reported in Table S7 and transported under refrigeration for analysis within 24 h. Hereafter, commercial/retail samples refer to finished, industrially processed and packaged dairy products purchased from retail outlets (produced from pooled milk and subjected to technological standardization), whereas farm-origin conventional samples refer to raw milk and/or farm-processed products collected directly from individual conventional (non-organic) farms.

#### Animal Feeding Systems and Botanical Composition of Pastures

Animals included in this study were raised under traditional feeding systems adapted to the geographic and climatic conditions of north-western Romania. During the spring–summer grazing season, cows, buffaloes, goats, and donkeys grazed freely on natural mountain pastures characterized by diverse spontaneous flora dominated by perennial grasses and legumes (e.g., *Festuca* spp., *Poa pratensis*, *Lolium perenne*, *Trifolium* spp., *Medicago sativa*). These grasslands are recognized for their high mineral content and biodiversity, which may influence macro- and trace-element concentrations in milk. During the winter season, cows received controlled rations based on organic maize silage, alfalfa hay, and cereal-based concentrates, while buffaloes, goats, and donkeys were fed predominantly natural hay, reflecting their extended grazing period (8–9 months per year) in mountainous areas. All farms operated feeding systems free of synthetic growth promoters, antibiotics, and ionophore additives.

### 2.2. Standardized Technological Procedures for the Production of Dairy Matrices

Milk samples (cow, buffalo, goat, and donkey) were obtained under hygienic conditions from organic, conventional, and commercial production systems. Immediately after milking, raw milk was cooled to 4–8 °C and homogenized to ensure uniform composition. Depending on the production system and species, milk was either maintained raw (organic samples) or subjected to pasteurization using standard industrial regimes, including high-temperature short-time (HTST; 72–75 °C for 15–20 s) or low-temperature long-time (LTLT; 65–70 °C for 30 min) treatments, followed by rapid cooling to 4 °C [25]. Cheese and yogurt samples were produced according to conventional dairy processing practices specific to each product category. Cheese manufacture involved milk pasteurization, starter culture inoculation, rennet-induced coagulation, curd cutting, molding, and short-term ripening under controlled temperature and humidity conditions. Yogurt production included milk standardization, heat treatment (85–95 °C), inoculation with thermophilic starter cultures (*Streptococcus thermophilus* and *Lactobacillus delbrueckii* subsp. *bulgaricus*), fermentation at 41–43 °C until pH 4.4–4.6, and rapid cooling to 4 °C, similar to previous applications [26]. Commercial dairy products were obtained as finished retail units in original sealed packaging and were not subjected to any additional processing prior to laboratory analysis. All samples were refrigerated at 2–4 °C and analyzed within 24 h after collection. Detailed, matrix-specific technological procedures for milk, cheese, mozzarella, and yogurt are provided in the Supplementary Material (Text S1).

### 2.3. Sample Collection Procedures

All dairy samples were collected under standardized aseptic conditions to ensure traceability, representativeness, and analytical reproducibility, in accordance with International Organization for Standardization (ISO) 707:2008 (Milk and Milk Products—Sampling) and Codex Alimentarius (Codex) CX/DS 234-2019 guidelines. Milk samples were collected from bulk cooling tanks after homogenization; the sampling valve was disinfected with 70% ethanol, and the first 200 mL were discarded. Aliquots (50–100 mL) were transferred into sterile polypropylene containers, labeled, stored at 2–4 °C, and transported to the laboratory within 2 h without freezing. Yogurt samples were randomly selected from sealed production or retail units. Aseptic subsamples (150–200 g) were collected from both upper and central layers, placed in sterile airtight containers, labeled, and transported at 2–4 °C, with laboratory delivery within 24 h. Cheese and mozzarella samples (150–200 g) were aseptically collected from both peripheral and internal sections using sterile tools, sealed in sterile containers, and transported at 2–4 °C to the laboratory within 24 h. Commercial products remained in original packaging until analysis.

### 2.4. Storage and Preservation of Samples

All dairy samples (milk, yogurt, cheese, and mozzarella) were stored immediately after collection under controlled refrigerated conditions (2–4 °C) to preserve physicochemical integrity and prevent microbial or compositional changes. Samples were maintained in hermetically sealed sterile containers or in original sealed commercial packaging, as appropriate. Temperature was continuously monitored using calibrated digital dataloggers. No freezing or chemical preservatives were applied in order to avoid alterations in mineral composition prior to analysis. All samples were processed analytically within 24 h of arrival at the laboratory.

### 2.5. Standardized Sample Preparation, Digestion, and Analytical Determination of Metals

#### 2.5.1. Standardized Sample Preparation and Microwave Digestion of Dairy Matrices

All dairy matrices, including organic and commercial milk (cow, goat, buffalo, and donkey), organic and commercial yogurt, organic and commercial cow cheese, and buffalo mozzarella, were prepared following a standardized protocol prior to trace metal determination (Pb, Cd, Hg, As, Cr, Ni, Al, Sn, Cu, and Zn). Liquid samples (milk and yogurt) were homogenized by gentle manual inversion, whereas solid samples (cheese and mozzarella) were homogenized using a sterile stainless-steel laboratory blender (Waring Laboratory Blender, Model 8011BG, Waring Commercial, McConnellsburg, PA, USA). A representative analytical portion of 0.50 ± 0.01 g (cheese and mozzarella) or 0.50 ± 0.01 mL (milk and yogurt) was accurately weighed into high-pressure PTFE vessels for microwave digestion.

For each sample, 5 mL of ultrapure nitric acid (HNO_3_, 65%, TraceMetal™ Grade, Merck Supelco, Darmstadt, Germany) and 2 mL of hydrogen peroxide (H_2_O_2_, 30%, Trace Analysis Grade, Sigma-Aldrich, St. Louis, MO, USA) were added. Microwave-assisted wet mineralization was performed using a Milestone START D Microwave Digestion System (Milestone Srl, Sorisole, Italy) under a controlled four-step temperature program reaching a maximum digestion temperature of 200 °C (Ramp 1: RT → 100 °C in 10 min, hold 5 min; Ramp 2: 100 °C → 150 °C in 10 min, hold 10 min; Ramp 3: 150 °C → 200 °C in 10 min, hold 20 min; Ramp 4: passive cooling to ≤60 °C for 20–30 min), as detailed in Table S8. Microwave digestion conditions for dairy matrices using the Milestone START D system are summarized in Table S8.

After digestion, vessels were opened only after cooling to ≤60 °C, and digests were quantitatively transferred into 25 mL Class A volumetric flasks (Brand GmbH + Co. KG, Wertheim, Germany), diluted to volume with ultrapure deionized water (18.2 MΩ·cm) (Merck Millipore, Darmstadt, Germany), and filtered, when necessary, through 0.45 μm PTFE membrane filters (Whatman™ PTFE, Cytiva, Cardiff, UK). Final solutions were stored in acid-washed polypropylene tubes at 4 °C until instrumental analysis (Eppendorf™ Safe-Lock Tubes, Eppendorf AG, Hamburg, Germany). All preparation procedures were conducted in a clean laboratory environment using trace-metal-free consumables to minimize contamination risk.

#### 2.5.2. Analytical Determination Procedures of Dairy Matrices

The quantification of trace metals in dairy matrices was performed using an ICP–MS system (Thermo Scientific iCAP Q, Bremen, Germany) operated under optimized instrumental conditions to ensure high analytical sensitivity and accuracy. The instrumental settings and data acquisition parameters employed for routine quantitative analysis are summarized in Table S9a,b. Helium collision mode with kinetic energy discrimination (He/KED) was used to minimize polyatomic interferences commonly associated with complex organic matrices such as milk. The instrument was tuned daily using a multi-element tuning solution to maximize signal stability and sensitivity across the mass range.

External calibration was performed using multi-element certified reference standards diluted in 2% HNO_3_ (ultrapure grade). Calibration curves were constructed using seven concentration levels selected to cover the expected analyte concentrations in dairy products. Linearity was evaluated using a linear regression model with an acceptance criterion of r^2^ ≥ 0.995 for all analytes. Internal standards (Bi, In, Ge, Sc, and Rh) were continuously introduced online to correct for matrix-induced suppression/enhancement, instrumental drift, and variability in nebulization efficiency. Detailed calibration levels, monitored isotopes, and collision mode conditions are shown in Table S10.

Method validation and quality assurance were verified by analysis of certified reference materials (CRM) and independent proficiency testing samples. The National Institute of Standards and Technology (NIST) SRM 1549 (Non-fat Milk Powder) was used to assess accuracy through percentage recovery calculations, while a Food Analysis Performance Assessment Scheme (FAPAS) Whole Milk QC material was used to evaluate performance through Z-score interpretation. Results demonstrated high analytical accuracy, with recoveries between 96 and 104% and Z-scores ranging from −0.17 to +0.15, all within accepted validation criteria. Full results are presented in Table S11 (CRM performance) and Table S12 (Z-score evaluation).

The instrumental limit of detection (LOD) for each element was defined as three times the standard deviation of ten replicate measurements of procedural blanks, divided by the slope of the external calibration curve. Concentrations below the LOD were reported as “below the limit of detection” (BLD) and treated as non-quantifiable values.

To characterize the extent of non-detects within each dataset, the left-censoring percentage (LCD) was calculated as the proportion (%) of samples with concentrations < LOD for a given analyte. Thus, LCD = 100% indicates that all measurements were below the detection limit, whereas LCD = 0% indicates that all samples were fully quantified.

For descriptive statistics, BLD values were not included in the calculation of means and standard deviations. In datasets with partial censoring (0% < LCD < 100%), summary statistics are therefore based only on quantified values. In cases of complete censoring (LCD = 100%), only qualitative statements (“<LOD”) are reported, and no numerical summary statistics are provided.

### 2.6. Ethical Considerations

This study was conducted in compliance with ethical and legal standards for research involving food products. Ethical approval was granted by the Bioethics Committee of the University of Agricultural Sciences and Veterinary Medicine Cluj-Napoca (Decision No. 529/3 October 2025), confirming adherence to applicable research integrity requirements and national regulations. No animals were directly involved in experimental procedures, and milk samples were collected exclusively from routine production processes without altering animal welfare conditions. The analyses were carried out according to European Commission Regulation (EU) 2023/915 establishing maximum permissible levels for Pb, Cd, Hg, and As in milk and dairy products [9]. Quality assurance was ensured through certified reference materials and proficiency testing evaluation, guaranteeing scientific reliability and traceability of analytical results.

### 2.7. Statistical Analysis

Statistical analysis was performed using IBM SPSS Statistics 29.0 and GraphPad Prism 10. Descriptive statistics (mean, standard deviation (SD), coefficient of variation (CV%), confidence interval (CI95%), skewness, kurtosis) were calculated, and data normality was assessed using the Shapiro–Wilk test (*p* < 0.05). Group differences were evaluated using one-way ANOVA with Tukey post hoc test or Kruskal–Wallis test with Dunn correction, depending on data distribution. Pearson or Spearman correlation coefficients were applied as appropriate, and statistical significance was set at *p* < 0.05.

## 3. Results

### 3.1. Distribution of Metal Concentrations Across Dairy Matrices by Species and Processing Stage

The analysis of mean concentrations (Table 1) and statistical distributions across datasets. Tables S13–S34 reveal a coherent elemental structure in all investigated dairy matrices. Toxic metals (Pb, Cd, Hg, As) occur at low levels, with Hg consistently below the limit of detection in all datasets left-censoring percentage (LCD = 100%) and Cd frequently non-detectable, whereas transition and essential elements (Cr, Ni, Al, Cu, Zn, Sn) are systematically quantified and dominate the elemental profiles (Table 1 and Tables S13–S34). Organic cow milk (dataset: Table S13; summary: Table S14) shows the lowest values and the narrowest dispersion: Pb = 0.00842 mg/kg (range: 0.00467–0.01166 mg/kg), Cd = 0.00107 mg/kg, As = 0.00105 mg/kg; all elements are fully quantified (LCD = 0%), while Hg is entirely below detection (LCD = 100%). For essential elements, mean concentrations include Al = 0.0213 mg/kg, Cu = 0.0904 mg/kg, Zn = 3.984 mg/kg, and Sn ≈ 0.0030 mg/kg (Table 1). Distributional diagnostics indicate approximate normality for most analytes (Shapiro–Wilk *p* > 0.05), with Ni (*p* = 0.056) and Cu (*p* = 0.076) near the significance threshold (Table S14). Conventional goat milk (Tables S15 and S16) maintains low levels of toxic metals (Pb = 0.00607 mg/kg; Cd = 0.000810 mg/kg; As = 0.000736 mg/kg), with Hg fully undetectable (LCD = 100%), but exhibits greater variability for As (RSD = 34.82%), Ni (RSD = 31.09%), and Al (RSD = 27.39%). Point values for essential elements include Al = 0.0218 mg/kg, Cu = 0.0786 mg/kg, Zn = 3.245 mg/kg, and Sn ≈ 0.0032 mg/kg (Table 1). Broader ranges are observed for Cr (0.0076–0.0174 mg/kg), Ni (0.0061–0.0178 mg/kg), and Al (0.0120–0.0320 mg/kg) (Table S15), with near-normal distributions (Table S16).

Buffalo milk (Tables S17 and S18) displays markedly higher concentrations for several elements: Cr = 0.3772 mg/kg, Ni = 0.3940 mg/kg, Al = 0.2635 mg/kg, Cu = 0.2697 mg/kg, Zn = 7.9628 mg/kg, and Sn ≈ 0.0035 mg/kg (Table 1). Dispersion is high for multiple analytes (e.g., Cd RSD = 65.15%; Al RSD = 50.60%; Zn RSD = 45.26%), with partial censoring for As (LCD = 27.5%); Hg remains undetectable (Table S18). Donkey milk (Tables S19 and S20) also shows elevated heterogeneity: As = 0.06641 mg/kg (RSD = 60.79%), with RSD values of approximately 44–50% for Cr, Ni, Al, and Sn; Hg is fully censored (LCD = 100%), and As is partially censored (LCD = 26.67%) (Table S20). Mean essential-element levels include Al ≈ 0.185 mg/kg, Cu ≈ 0.112 mg/kg, Zn ≈ 4.98 mg/kg, and Sn ≈ 0.0034 mg/kg (Table 1). Organic cow-milk yogurt (Tables S23 and S26) shows Pb at very low levels, with Cd, Hg, and As predominantly below detection, while Cr, Ni, Al, Cu, Zn, and Sn are consistently quantified. Mean values include Al = 0.0285 mg/kg, Cu = 0.1214 mg/kg, Zn = 4.761–5.654 mg/kg, and Sn ≈ 0.0033 mg/kg (Table 1). Distributions are relatively tight (e.g., Zn RSD = 8.29%) (Table S26). Buffalo-milk yogurt (Table S27) follows the same pattern: Cd and Hg are fully non-detectable, Pb is very low, and essential elements are consistently quantified, with higher levels for several metals: Al = 0.0312 mg/kg, Cu = 0.21193 mg/kg, Zn = 6.53444 mg/kg, and Sn ≈ 0.0036 mg/kg (Table 1). No extreme values are observed (Tables S23–S27). Buffalo-milk cheese (Tables S29 and S30) exhibits Cd and Hg fully non-detectable and As largely censored (LCD = 86.7%), together with systematic increases in essential elements: Cr = 0.0146 mg/kg, Ni = 0.0124 mg/kg, Al = 0.0211 mg/kg, Cu = 0.1816 mg/kg, Zn = 6.021 mg/kg, and Sn = 0.0030 mg/kg (Table 1). Zn shows low dispersion (RSD = 4.34%), and Sn is invariant across samples (Table S30).

Buffalo mozzarella (Tables S33 and S34) maintains complete censoring for Cd and Hg and partial censoring for As; mean concentrations are Cr = 0.0186 mg/kg, Ni = 0.0181 mg/kg, Al = 0.0347 mg/kg, Cu = 0.2422 mg/kg, Zn = 7.243 mg/kg, and Sn ≈ 0.0038 mg/kg (Table 1), with low dispersion for quantified elements (e.g., Zn RSD = 6.49%; Cr RSD = 10.99%; Al RSD = 10.00%) (Table S34). Commercial cow cheese (Tables S31 and S32) shows Pb = 0.00946 mg/kg, complete censoring for Cd and Hg, partial censoring for As (LCD = 75%), and higher essential-element concentrations than in milk and yogurt: Al = 0.0294 mg/kg, Cu = 0.1987 mg/kg, Zn = 6.785 mg/kg, and Sn ≈ 0.0035 mg/kg (Table 1), with moderate variability for Pb and Ni (Table S32). Across fermented and solid products, Sn remains stable and low (≈0.003–0.004 mg/kg), with no anomalous values (Tables S23–S34).

**Figure 1:**
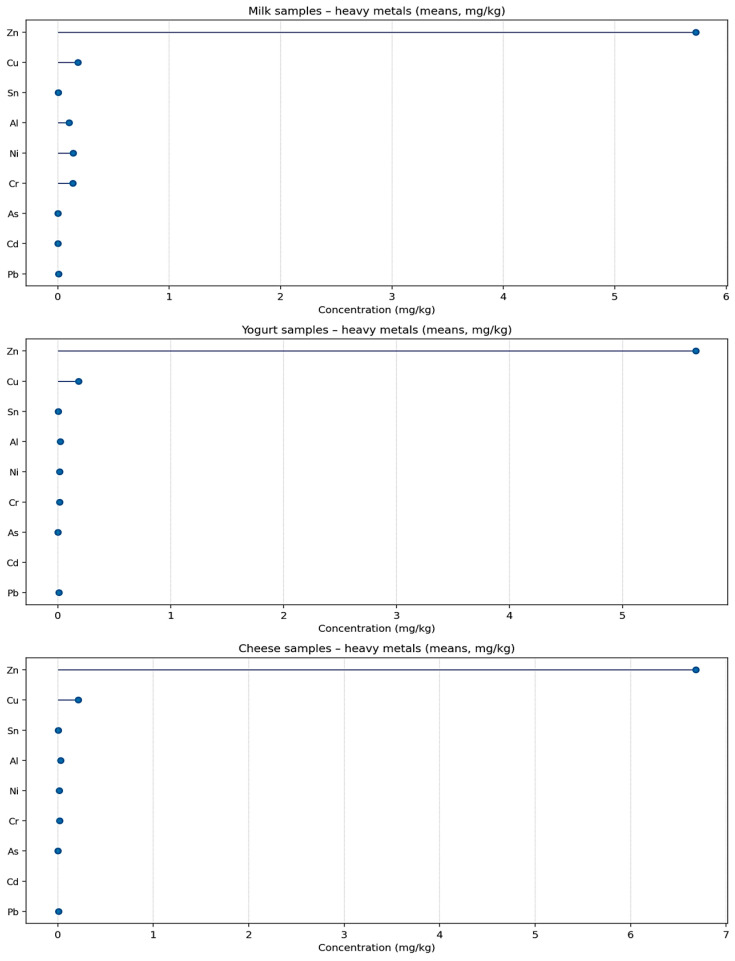
Mean concentrations (mg/kg) of heavy and essential metals in milk, yogurt, and cheese products by species and processing type. Overall distribution of toxic and essential metal concentrations across all dairy matrices and animal species, highlighting inter-species variability in elemental profiles. This figure provides a global overview of concentration ranges and relative abundances for each analyte, emphasizing differences among cow, buffalo, goat, and donkey milk and derived products, irrespective of processing stage or production system.

For most metals, the proportion of censored data is low; exceptions are Hg (LCD = 100% in all datasets) and Cd/As in certain processed products. Distributional metrics (skewness, kurtosis) and normality tests reported in the summary tables (Tables S14, S16, S18, S20, S26, S30, S32 and S34) indicate no extreme departures for quantified elements. Overall, Table 1 and Tables S13–S34 document systematic differences among species and product types, together with higher concentrations of essential elements in solid matrices relative to liquid and fermented products. To facilitate a comparative overview of these distribution patterns across species and processing stages, the main trends are summarized graphically in Figure 1. The figure illustrates a clear stratification of mean elemental concentrations across dairy matrices as a function of both species and product type. For all categories, toxic metals (Pb, Cd, Hg, As) occur at low levels, with Hg uniformly below detection and Cd largely non-detectable outside selected raw-milk matrices. In contrast, transition and essential elements (Cr, Ni, Al, Cu, Zn) dominate the compositional profiles. Among milk products, organic cow and conventional goat milk display the lowest overall metal burdens, while buffalo milk is distinctly characterized by substantially higher mean concentrations of Cr, Ni, Al, Cu, and Zn. This separation is consistently preserved in Figure 1, indicating a species-dependent elemental signature that is evident already at the raw-milk stage. Across processing types, Figure 1 shows a systematic increase in mean concentrations from milk to yogurt and further to cheese for most quantified elements. Fermented products retain the same detectability patterns as their corresponding milks, with Cd, Hg, and As remaining below detection and only modest increases observed for Cr, Ni, Al, Cu, and Zn.

The inter-species differences observed in elemental profiles can be interpreted in light of the distinct feeding systems and pasture characteristics. Buffaloes, goats, and donkeys grazed for extended periods (8–9 months per year) on natural mountain pastures with diverse spontaneous flora (*Festuca* spp., *Poa pratensis*, *Lolium perenne*, *Trifolium* spp., *Medicago sativa*), whereas cows received more controlled winter rations based on maize silage, alfalfa hay, and cereal-based concentrates. This prolonged exposure to heterogeneous pastures and variable soil–plant interfaces may partly explain the higher concentrations and greater dispersion of Cr, Ni, Al, and Zn recorded in buffalo and donkey milk compared to cow milk.

In particular, the elevated levels of Cr and Ni in buffalo milk, together with the higher Al and Zn contents in both buffalo and donkey milk, are consistent with increased ingestion of trace elements through soil-contaminated herbage and mineral-rich forage typical of mountain grasslands. Moreover, species-specific grazing behavior and digestive physiology (e.g., greater roughage intake and differences in rumen or hindgut fermentation efficiency) likely modulate trace-element bioavailability and transfer into milk. By contrast, the more homogeneous elemental composition of organic cow milk reflects the greater dietary standardization and reduced environmental variability associated with controlled feeding practices. These findings indicate that feeding systems and pasture composition are not only contextual descriptors but key drivers of the inter-species variability observed in the present dataset, reinforcing the importance of integrating agro-ecological factors into the interpretation of milk multi-elemental profiles.

Cheese matrices exhibit the highest mean values for these elements, with particularly pronounced enrichment of Zn and Cu, while maintaining low Pb and non-detectable Cd and Hg. The consistent positioning of buffalo-derived products at the upper end of the concentration scale, and of cow- and goat-derived products at lower levels, demonstrates that processing amplifies existing matrix-specific elemental profiles rather than altering their relative structure. Collectively, Figure 1 confirms a stable, ordered distribution of metals governed jointly by species and product type, with minimal representation of toxic elements and progressive enrichment of essential metals in solid dairy matrices.

Figure 1 shows the mean concentrations of heavy metals determined in milk, yogurt, and cheese samples. The figure legend was designed to clearly identify each analyzed metal, and the axis labels specify the corresponding measurement units (mg/kg), ensuring correct interpretation of the data. This presentation allows the reader to easily follow the variations in metal concentrations within each type of dairy product and to compare the relative values among the investigated metals.

**Figure 2:**
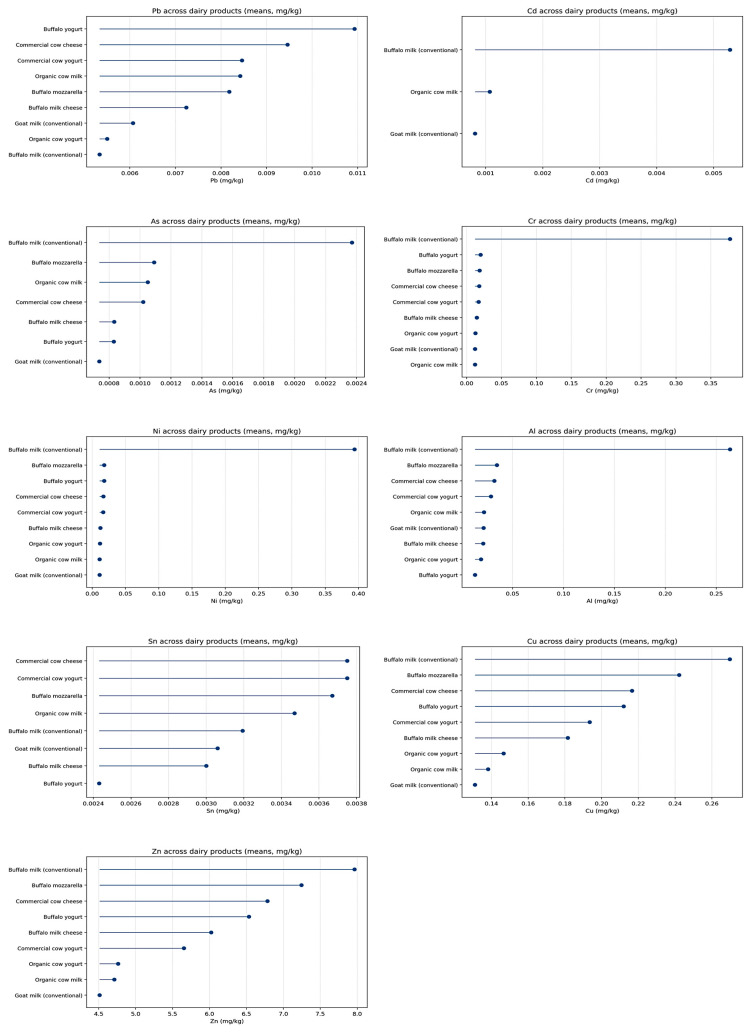
Comparative profiles of mean metal concentrations (mg/kg) in milk, yogurt, and cheese products, stratified by animal species and processing stage. Comparative profiles of metal concentrations by processing stage (raw milk, yogurt, cheese, and mozzarella), illustrating the effect of technological processing on elemental partitioning and concentration patterns. This figure focuses on matrix-driven differences and highlights how fermentation and coagulation influence the distribution of toxic and essential elements across dairy products.

Figure 2 demonstrates a clear separation between toxic trace metals (Pb, Cd, As) and transition and essential elements (Cr, Ni, Al, Sn, Cu, Zn) across all dairy categories. Lead exhibits low and relatively narrow mean values among products, with slightly higher levels in buffalo-derived and certain commercial products, but without disproportionate increases. Cadmium is detectable essentially only in conventional buffalo milk, while it remains below the detection limit in the other matrices. Arsenic occurs at trace levels (µg/kg range), with the highest mean again in buffalo milk and lower values in yogurts and cheeses. In contrast, Cr, Ni, and Al display pronounced inter-species differentiation: buffalo milk clearly exhibits the highest mean concentrations for these elements, whereas cow and goat milks cluster at substantially lower levels. This consistent pattern across the individual subplots in Figure 2 indicates that elemental profiles are strongly species-dependent already at the raw-milk stage. With respect to product type, Figure 2 shows a systematic increase in mean concentrations for most quantified elements from milk to yogurt and further to cheese. This trend is particularly evident for Zn and Cu, which reach their highest levels in solid matrices (cheeses and mozzarella), intermediate values in yogurts, and the lowest means in milk.

To avoid potential misinterpretation arising from the markedly different concentration orders of magnitude among the analyzed elements, all elemental concentrations in Figure 2 are reported in a single standardized unit (mg/kg). It should be noted that essential elements such as Zn and Cu occur at mg/kg levels, whereas toxic trace metals (Pb, Cd, As) and several transition elements (Cr, Ni, Al, Sn) are present at much lower concentrations, typically in the sub-mg/kg or trace range. Consequently, when displayed on a common linear scale, low-level toxic elements may appear visually compressed toward zero. This graphical effect reflects true concentration differences rather than inconsistencies in measurement units. The figure is therefore intended to illustrate relative magnitude patterns across matrices rather than absolute comparability among all elements on a single scale.

Tin remains uniformly low and stable across all product categories, without anomalous values. At the same time, toxic metals retain their low-detectability pattern throughout processing: Cd remains largely undetectable outside buffalo milk, and As persists only at very low levels. Collectively, Figure 2 indicates that technological processing amplifies existing matrix-specific elemental profiles rather than altering their relative structure, with essential elements becoming progressively enriched from liquid to fermented and solid dairy products while toxic metals remain consistently low.

### 3.2. Comparison of Metal Concentrations Between Production Systems (Organic, Conventional, Commercial)

The comparative analysis of metal concentrations in milk, yogurt, and cheese from organic and commercial/retail systems indicates systematic differences between production types. Based on the Mann–Whitney U test, statistically significant differences were observed for most of the analyzed elements, as detailed in Table 2, Table 3 and Table 4.

In the case of milk (Table 2), higher concentrations in the commercial/retail system were observed for most of the analyzed metals, including Cd, As, Cr, Ni, Al, and Sn, with all differences being highly statistically significant (*p* < 0.001). For example, Al was more than five times higher in commercial milk (0.12260 mg/kg) than in organic milk (0.02207 mg/kg), while Cr and Ni showed approximately threefold higher mean values in commercial products. In contrast, Zn was significantly higher in organic milk (4.719 mg/kg) compared with commercial milk (0.468 mg/kg) (*p* < 0.001). No statistically significant differences were observed for Pb and Cu (*p* ≥ 0.05). Mercury (Hg) was below the limit of detection in all samples.

The data for yogurt (Table 3) show that differences between production systems persist after processing. Concentrations of Pb, As, Cr, Ni, Cu, and Zn were significantly higher in commercial products, with most elements showing very high statistical significance (*p* < 0.001). Arsenic (As) was below the limit of detection in organic yogurt but was detected in commercial yogurt (0.00360 mg/kg). No statistically significant differences were observed for Al and Sn (*p* ≥ 0.05). Zinc (Zn) was significantly higher in commercial yogurt (5.65350 mg/kg) compared with organic yogurt (4.76100 mg/kg) (*p* < 0.001).

In cheese (Table 4), a product characterized by a high degree of concentration of mineral substances, the differences become even more evident for most metals.

For this dairy product, Pb, Cr, Ni, Al, Cu, and Zn showed higher mean concentrations in commercial cheeses than in organic cheeses, with significant differences for Pb (*p* = 0.0340) and for Cr, Ni, Al, Cu, and Zn (*p* ≤ 0.001). Although As did not show a statistically significant difference between production systems, mean values were higher in the commercial system, consistent with the trend observed in milk and yogurt. Cadmium (Cd) and mercury (Hg) were below the limit of detection in both types of cheese. Across the three product categories, a consistent pattern was observed, with commercial/retail products generally exhibiting higher mean concentrations of Pb, Cd, As, Cr, Ni, Al, and Sn, while organic products showed higher values for certain essential elements in specific cases (e.g., Zn in milk).

Differences between production systems were observed across multiple elements and product categories (Table 2, Table 3 and Table 4), indicating systematic variability in metal concentrations between organic and commercial/retail samples. These differences were maintained across milk, yogurt, and cheese, indicating consistent variation in trace element concentrations among product categories, with higher mean values for several elements in cheese. Although absolute metal concentrations were low across all samples, statistically significant differences between production systems were observed for several elements, including both potentially toxic and essential metals (Table 2, Table 3 and Table 4).

### 3.3. Effect of Processing on Metal Concentrations in Dairy Products (Milk, Yogurt, Cheese)

Table 5 summarizes the effect of processing on metal concentrations in dairy products (milk, yogurt, and cheese), expressed as mean values (mg/kg, wet weight). Table 6 presents the corresponding results for commercial/retail products. Statistically significant differences were evaluated using the Mann–Whitney U test based on milk–cheese comparisons.

For lead (Pb), mean concentrations in the general dataset (Table 5) were 0.00809 mg/kg in milk, 0.00550 mg/kg in yogurt, and 0.00727 mg/kg in cheese, with a significant difference between milk and cheese (*p* = 0.0340). In commercial/retail products (Table 6), Pb increased progressively from milk (0.00667 mg/kg) to yogurt (0.00846 mg/kg) and cheese (0.00946 mg/kg), also showing a significant milk–cheese difference (*p* = 0.0340).

Cadmium (Cd) was detected in milk (0.00106 mg/kg in the general dataset, Table 5; 0.00186 mg/kg in commercial products, Table 6) but was below the limit of detection (BLD) in both yogurt and cheese; therefore, no statistical comparison was performed. Mercury (Hg) was below the detection limit in all product types in both datasets (Table 5 and Table 6).

Arsenic (As) decreased across products. In the general dataset (Table 5), mean concentrations were 0.00106 mg/kg in milk, BLD in yogurt, and 0.00073 mg/kg in cheese, with a non-significant difference between milk and cheese (*p* = 0.1700). In commercial/retail products (Table 6), As decreased from 0.00425 mg/kg in milk to 0.00360 mg/kg in yogurt and 0.00150 mg/kg in cheese, also without statistical significance (*p* = 0.1700).

Chromium (Cr) showed mean values of 0.01199 mg/kg in milk, 0.01270 mg/kg in yogurt, and 0.01093 mg/kg in cheese in the general dataset (Table 5), with a highly significant milk–cheese difference (*p* = 0.000102). In commercial/retail products (Table 6), Cr decreased from 0.03315 mg/kg in milk to 0.01765 mg/kg in yogurt and 0.01775 mg/kg in cheese, also with a highly significant milk–cheese difference (*p* = 0.000102).

Nickel (Ni) followed a similar pattern. In the general dataset (Table 5), mean concentrations were 0.01121 mg/kg in milk, 0.01174 mg/kg in yogurt, and 0.01053 mg/kg in cheese, with a significant difference between milk and cheese (*p* = 0.000111). In commercial/retail products (Table 6), Ni decreased from 0.02760 mg/kg in milk to 0.01805 mg/kg in yogurt and 0.01820 mg/kg in cheese, again with a significant milk–cheese difference (*p* = 0.000111).

Aluminum (Al) increased across processing stages in the general dataset (Table 5), from 0.02207 mg/kg in milk to 0.02850 mg/kg in yogurt and 0.03200 mg/kg in cheese, with a highly significant milk-cheese difference (*p* = 0.0000004). In commercial/retail products (Table 6), Al decreased from 0.12260 mg/kg in milk to 0.04195 mg/kg in yogurt, followed by a slight increase in cheese (0.04975 mg/kg); the overall milk-cheese comparison remained highly significant (*p* = 0.0000004). Copper (Cu) increased with processing in both datasets. In the general dataset (Table 5), Cu rose from 0.13917 mg/kg in milk to 0.14667 mg/kg in yogurt and 0.15900 mg/kg in cheese (*p* = 0.000294). In commercial/retail products (Table 6), Cu increased from 0.14635 mg/kg in milk to 0.19350 mg/kg in yogurt and 0.21650 mg/kg in cheese, with a highly significant milk–cheese difference (*p* = 0.000294).

Zinc (Zn) showed the most pronounced increase. In the general dataset (Table 5), Zn increased from 4.71900 mg/kg in milk to 4.76100 mg/kg in yogurt and 6.42170 mg/kg in cheese, with a highly significant difference between milk and cheese (*p* = 0.000406). In commercial/retail products (Table 6), Zn rose markedly from 0.46810 mg/kg in milk to 5.65350 mg/kg in yogurt and 6.78500 mg/kg in cheese, also with a highly significant milk–cheese difference (*p* = 0.000406).

Tin (Sn) remained relatively stable or decreased slightly. In the general dataset (Table 5), Sn values were 0.00349 mg/kg in milk, 0.00330 mg/kg in yogurt, and 0.00343 mg/kg in cheese, with no significant difference between milk and cheese (*p* = 0.1090). In commercial/retail products (Table 6), Sn decreased from 0.01305 mg/kg in milk to 0.00375 mg/kg in both yogurt and cheese, also without statistical significance (*p* = 0.1090).

### 3.4. Food Safety Evaluation of Dairy Products Based on EU Maximum Permissible Levels

**Figure 3:**
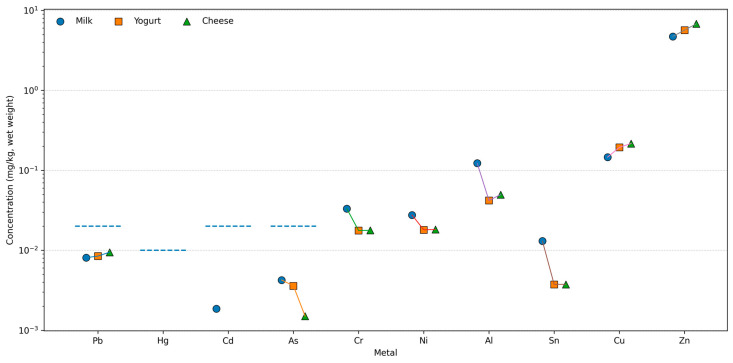
Processing-Related Changes in Metal Concentrations in Dairy Products Relative to EU Maximum Permissible Levels. Mean concentrations of heavy metals in milk, yogurt, and cheese samples (wet weight). The dashed blue line indicates the maximum permissible levels established by European Union legislation: Pb = 0.020 mg/kg, Cd = 0.010 mg/kg, Hg = 0.010 mg/kg, and As = 0.100 mg/kg.

Figure 3 presents the measured concentrations of metals in milk, yogurt, and cheese on a logarithmic scale, together with the corresponding European Union maximum permissible levels (MLs) for regulated elements [9]. The graphical representation allows direct comparison of processing-related changes and regulatory compliance across all analyzed matrices.

For lead (Pb), concentrations ranged from approximately 0.008–0.009 mg/kg in milk, decreased slightly in yogurt (≈0.008–0.0085 mg/kg), and increased modestly in cheese (≈0.009–0.0095 mg/kg). In all three matrices, Pb levels remained clearly below the EU maximum level of 0.020 mg/kg. Mercury (Hg) was below the limit of detection in milk, yogurt, and cheese, and therefore remained well below the EU regulatory threshold of 0.010 mg/kg across all processing stages. Cadmium (Cd) was detected only in milk at low concentrations (approximately 0.001–0.002 mg/kg) and was below the limit of detection in both yogurt and cheese. All values were substantially lower than the EU maximum levels defined for milk-based products. Arsenic (As) showed a decreasing trend with processing: concentrations were approximately 0.004–0.005 mg/kg in milk, declined in yogurt (≈0.003–0.004 mg/kg), and reached the lowest values in cheese (≈0.001–0.002 mg/kg). In all matrices, As concentrations were well below the regulatory limits applicable to milk-based products.

For chromium (Cr), concentrations were highest in milk (≈0.03 mg/kg), decreased in yogurt (≈0.018 mg/kg), and remained at similar levels in cheese (≈0.018 mg/kg). Nickel (Ni) followed a comparable pattern, with values of approximately 0.028 mg/kg in milk, decreasing to ≈ 0.018 mg/kg in yogurt and remaining stable in cheese. Aluminum (Al) showed a pronounced decrease from milk (≈0.12 mg/kg) to yogurt (≈0.04 mg/kg), followed by a slight increase in cheese (≈0.05 mg/kg). Tin (Sn) exhibited a reduction from milk (≈0.013 mg/kg) to yogurt (≈0.004 mg/kg), with similar concentrations in cheese. Copper (Cu) increased progressively across processing stages, from ≈0.15 mg/kg in milk to ≈0.19 mg/kg in yogurt and ≈0.22 mg/kg in cheese. Zinc (Zn) showed the most pronounced increase, rising from ≈4.7 mg/kg in milk to ≈5.6 mg/kg in yogurt and reaching ≈6.8 mg/kg in cheese. For metals without established EU maximum levels (Cr, Ni, Al, Sn, Cu, and Zn), Figure 3 illustrates the magnitude and direction of concentration changes during processing without regulatory reference lines. For all regulated metals (Pb, Hg, Cd, and As), the measured concentrations in milk, yogurt, and cheese remained consistently below the corresponding EU maximum permissible levels throughout all processing stages.

## 4. Discussion

### 4.1. Comparative Interpretation of Elemental Contamination Across Dairy Species

A clear species-dependent pattern was observed across dairy matrices. Cow and goat milk showed low and relatively stable elemental concentrations, with mercury consistently below detection limits, indicating minimal contamination. Chromium and nickel were the most frequently detected elements in these matrices and remained within ranges compatible with natural or technological sources. Commercial cow milk exhibited further reduced variability due to industrial homogenization. In contrast, buffalo and donkey milk displayed higher concentrations and pronounced inter-sample variability across multiple elements, including Cd, Cr, Ni, Al, Cu, and Zn. These differences indicate enhanced metal transfer and greater sensitivity to environmental and management-related factors. Overall, species-specific physiological characteristics represent the primary driver of elemental variability in raw milk, with buffalo and donkey milk requiring increased monitoring compared to cow and goat milk.

### 4.2. Comparative Interpretation of Elemental Contamination Across Yogurt Matrices

All yogurt matrices exhibited uniformly low levels of toxic metals, with Cd and Hg consistently below detection limits and As only sporadically detected at trace concentrations. Lead occurred at low levels across products, while Cr, Ni, Al, Cu, and Zn were consistently quantified with limited variability. These patterns indicate that fermentation and standardized processing largely attenuate species-related differences observed in raw milk, making technological and matrix effects the primary drivers of elemental composition in yogurt.

### 4.3. Comparative Interpretation of Elemental Contamination Across Cheese Matrices

All cheese matrices exhibited low levels of toxic metals, with Cd and Hg consistently below detection limits and As only sporadically detected at trace concentrations. Lead occurred intermittently at low levels, consistent with diffuse background exposure. In contrast, Cr, Ni, Al, Cu, and Zn were consistently quantified with narrow concentration ranges, reflecting the combined effects of intrinsic milk composition and standardized cheese-making processes. Tin was detected at low levels without evidence of accumulation, while Zn showed the highest concentrations, consistent with its natural abundance in dairy products. Overall, these results indicate a high degree of compositional stability across cheese types, confirming the effectiveness of current processing practices in limiting toxic metal transfer.

### 4.4. Global Elemental Fingerprint of Dairy Matrices: Descriptive Patterns, Statistical Validation, and Processing Effects

**Figure 4:**
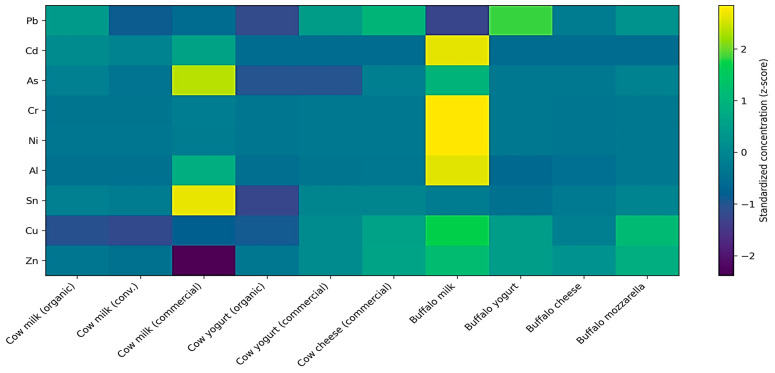
Global elemental fingerprint of dairy matrices across species, production systems, and processing stages. The heatmap displays element-wise z-score–standardized mean concentrations across dairy matrices, emphasizing relative differences among products, species, and processing stages rather than absolute concentrations. Reduced color contrast for certain elements (e.g., Cd and As) reflects limited variability due to extensive left-censoring and low dispersion after standardization. Consequently, the visual structure of the elemental fingerprint is primarily driven by elements exhibiting consistent quantification and higher variability across matrices.

**Figure 5:**
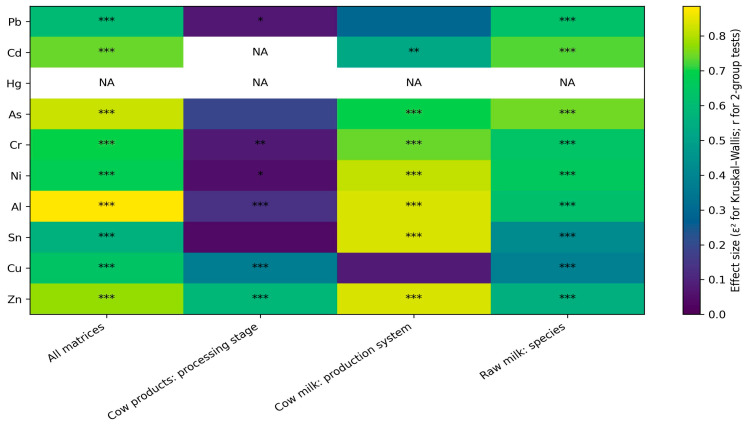
Statistical effect size and significance map of elemental differences across dairy matrices. Heatmap visualization of effect sizes (ε^2^ for Kruskal–Wallis tests; r for two-group comparisons) for all analyzed elements across major experimental factors, including processing stage, production system, and species. Color intensity represents the magnitude of the effect size, while asterisks denote statistical significance (* *p* < 0.05; ** *p* < 0.01; *** *p* < 0.001). Cells labeled NA indicate insufficient quantifiable data due to extensive left-censoring.

Processed dairy products exhibit higher standardized values for Cu and Zn compared with raw milk, with the highest values observed in cheese matrices (Figure 4). A consistent processing-related gradient from milk to yogurt and cheese is evident across all product categories. These visually observed patterns were statistically evaluated using effect size and significance analysis for all elements (Figure 4). In addition, processing factor (PF) analysis was performed to quantify elemental changes induced by technological processing along the milk–yogurt–cheese chain (Figure 5).

Species-related differentiation is also evident for several elements. Matrices derived from buffalo milk are characterized by higher standardized values of Al, Ni, and Zn compared with the corresponding cow milk products, a distinction observed consistently in both raw and processed matrices. This visual separation is coherent across all analyzed matrices and contributes substantially to the overall structure of the elemental fingerprint. In contrast, toxic trace elements, particularly Pb and Cd, display limited chromatic variability across the figure, indicating relatively small differences in mean concentrations among matrices. Elements affected by extensive left-censoring, such as Cd and As, contribute marginally to global differentiation and are represented by similar standardized values across products and production systems.

Differences associated with production systems (organic, conventional, and commercial) are less pronounced than those related to species and processing stage. Such differences are observable only locally for selected elements, notably Al and Zn, without altering the overall distribution patterns highlighted in Figure 4. Elements exhibiting extensive left-censoring or limited variance, such as Cd and As, display reduced color contrast in the heatmap representation due to low dispersion following z-score standardization. Consequently, these elements contribute minimally to the discrimination among dairy matrices, with the global elemental fingerprint being primarily driven by elements that are consistently quantified and exhibit higher variability across products.

The effect size and statistical significance map (Figure 5) provides a comprehensive quantitative evaluation of elemental differences across dairy matrices, explicitly assessing the influence of processing stage, production system, and species for all analyzed elements. Among the tested factors, the processing stage exerts the strongest and most consistent effect, with multiple elements exhibiting moderate to high effect sizes across cow-derived products. In particular, Cu and Zn show robust and statistically significant differentiation between milk, yogurt, and cheese, indicating a systematic concentration shift associated with fermentation and ripening. In contrast, toxic trace elements such as Pb and Cd display uniformly low effect sizes, reflecting limited statistical separation among matrices despite complete inclusion in the analysis.

Species-related comparisons of raw milk further reveal measurable and statistically supported differences for selected elements, most notably Al and Ni, confirming inter-species variability as an independent contributor to elemental heterogeneity. By comparison, differences associated with production system (organic versus commercial cow milk) are characterized by smaller effect sizes and limited statistical significance, indicating a secondary influence relative to processing stage and species. Elements affected by extensive left-censoring or low variance, including Cd, As, and Hg, exhibit negligible or non-evaluable effect sizes, demonstrating that the global elemental fingerprint is predominantly driven by consistently quantified elements with sufficient dispersion across products and matrices.

**Figure 6:**
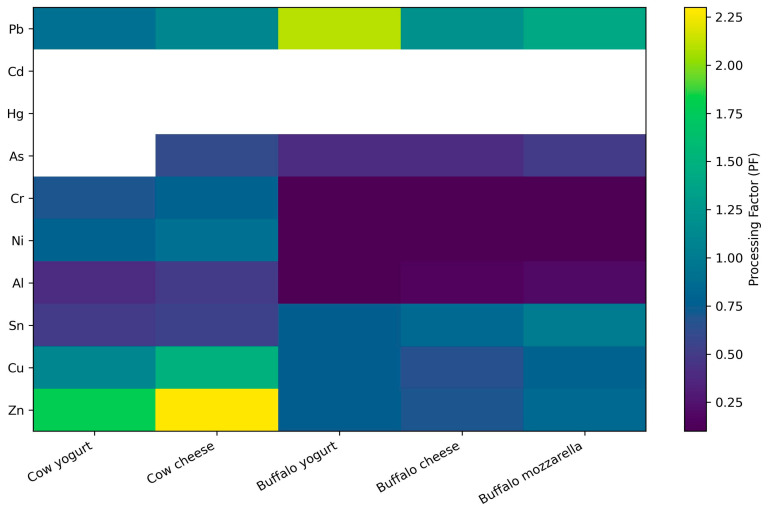
Processing factor (PF) heatmap illustrating elemental redistribution along the dairy processing chain (milk–yogurt–cheese). Processing factors (PF) were calculated as the ratio between mean elemental concentrations in processed products and the corresponding raw milk (PF = C_product/C_milk). PF values greater than unity indicate concentration during processing, whereas PF values close to or below unity indicate limited enrichment or dilution. Elements affected by extensive left-censoring or insufficient quantifiable data were excluded from comparative PF interpretation.

The processing factor (PF) heatmap (Figure 6) quantitatively illustrates element-specific redistribution patterns along the dairy processing chain from raw milk to yogurt and cheese. PF values exceeding unity are consistently observed for Cu and Zn in cheese matrices derived from both cow and buffalo milk, confirming a systematic concentration effect associated with technological processing.

Yogurt matrices display intermediate PF values for these elements, supporting a progressive enrichment trend across processing stages. In contrast, most toxic trace elements, including Pb and Cd, exhibit PF values close to or below unity, indicating limited or negligible concentration during processing. Elements affected by extensive left-censoring or insufficient quantifiable data, such as Cd, As, and Hg, were excluded from comparative PF interpretation, and consequently did not contribute to the dominant processing-driven patterns shown in Figure 5.

The observed enrichment or depletion of elements during dairy processing can be attributed to several interconnected physicochemical and technological mechanisms. One major factor is water loss and dry-matter concentration, particularly during cheese manufacture and yogurt fermentation, which leads to an apparent increase in the concentration of both essential and toxic elements in the solid phase. As moisture is removed or redistributed, elements that remain associated with the protein–mineral matrix become concentrated, resulting in processing factors (PF) greater than unity.

In addition, many trace elements (e.g., Zn, Cu, Fe, and Pb) exhibit strong affinity for milk proteins, especially caseins, through electrostatic interactions and complexation with phosphoserine residues. During coagulation and curd formation, protein-bound elements preferentially partition into the curd, while more weakly associated or highly soluble elements (e.g., K, Na, and in part Mg) are partially lost with the whey, contributing to element-specific enrichment or depletion patterns.

Microbial activity during fermentation may further modify elemental distribution. Lactic acid bacteria can alter pH and redox conditions, promoting changes in metal solubility and speciation, as well as limited biosorption of certain elements onto microbial cell walls. Acidification during yogurt production also facilitates the dissociation of some mineral complexes, enabling partial redistribution between the aqueous and protein-bound fractions.

Technological operations such as fat standardization, whey separation, and curd washing additionally influence elemental fate by selectively removing or retaining matrix fractions with different binding capacities. Elements preferentially associated with the aqueous phase may decrease in concentration following whey drainage, whereas those bound to proteins or retained in the curd matrix become enriched.

Collectively, these mechanisms—water loss, protein binding, whey separation, microbial activity, and technological fractionation—provide a mechanistic basis for the processing-dependent changes in elemental concentrations observed in this study and explain the element-specific variability of processing factors across different dairy matrices.

The levels of toxic elements determined in the present study are in strong agreement with international reports on milk and dairy products from non-industrially impacted areas. In our samples, Hg was below the limit of detection in all matrices, Cd was frequently non-detectable (≈0.0008–0.0053 mg/kg), As occurred at trace levels (≈0.0007–0.0024 mg/kg), and Pb ranged between ≈0.005 and 0.011 mg/kg. Similarly, low background concentrations of Pb, Cd, and As have been reported in buffalo and cow milk from Italy [27], Egypt [28], and Ethiopia [29], while studies from India indicate that elevated metal levels are mainly associated with industrial or polluted areas, whereas milk from residential or agricultural zones shows comparably low concentrations. Comparable patterns were also reported for artisanal cheeses, where Cd and Pb occurred at low levels and no health risk was identified [30], and where Hg was non-detectable and Cd and Pb were present at low concentrations with THQ and HI values below 1 [31]; these findings are further supported by surveys reporting generally low levels of Hg, As, Pb, Cd, Cr, and Cu and minimal consumer risk [28,32,33], as well as by studies showing that heavy metals in milk are typically within acceptable limits except in contaminated regions [19,34]. Regarding essential elements, our concentrations of Zn (≈4.5–8.0 mg/kg) and Cu (≈0.08–0.27 mg/kg) fall within the ranges reported for European and Asian systems [27,28,35] while the higher levels in yogurt and cheese relative to raw milk are consistent with processing-related concentration effects [36,37,38] and with evidence that processing modifies mineral distribution and may enhance bioaccessibility [39]. Our results also align with reports indicating that dairy matrices provide higher and more consistent levels of essential minerals than alternatives, with toxic elements remaining at trace or non-detectable levels [21,40,41,42], and the absence or very low levels of Pb, Ni, and Cd mirror observations across different regions [29], confirming that the elemental profiles of our dairy products are consistent with international data and comply with current food safety and nutritional benchmarks.

### 4.5. Multivariate Characterization of Dairy Matrices Based on Elemental Composition

**Figure 7:**
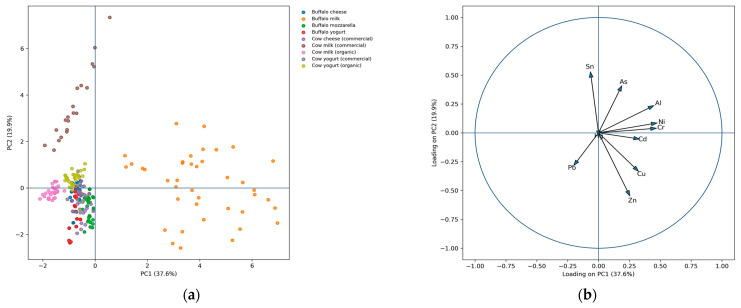
Principal component analysis (PCA) of elemental composition across dairy matrices: (**a**) score plot showing the distribution of milk, yogurt, and cheese samples; (**b**) loading plot illustrating the contribution of individual elements to the principal components.

The principal component analysis (PCA) score plot (Figure 7a) shows a clear multivariate separation of dairy matrices, with the first two principal components accounting for the majority of variance in the dataset. Samples are primarily structured along PC1 according to processing stage, with raw milk positioned at one end of the axis and processed products progressively separated, yogurt occupying intermediate positions and cheese forming the most distinct cluster. This gradient is consistently observed across all species and production systems, indicating that the processing stage represents the dominant contributor to variance in elemental composition. The spatial separation between milk, yogurt, and cheese clusters demonstrates that elemental profiles change systematically along the technological chain.

In addition to processing-related effects, the PCA scores reveal species-dependent differentiation. Buffalo-derived matrices are separated from cow-derived matrices along PC2, both for raw milk and processed products, indicating consistent interspecific differences in elemental composition. By comparison, samples grouped according to production system (organic, conventional, commercial) show substantial overlap within the PCA space, reflecting limited multivariate separation attributable to farming system alone. These results indicate that species and processing stage exert a stronger influence on the global elemental fingerprint than production system.

The PCA loading plot (Figure 7b) identifies the elements contributing most strongly to sample discrimination. Zn and Cu display the highest positive loadings along PC1, aligning with the separation of processed products from raw milk, while Al and Ni contribute substantially to species-related differentiation along PC2. In contrast, Pb, Cd, and As exhibit low loadings across both principal components, indicating minimal contribution to variance and limited discriminatory power. Elements affected by extensive left-censoring or low variance, therefore, contribute marginally to the multivariate structure, confirming that the observed separation among dairy matrices is primarily driven by consistently quantified elements with higher dispersion across products.

### 4.6. Network Showing Relationships Between Dairy Products and Detected Elements

**Figure 8:**
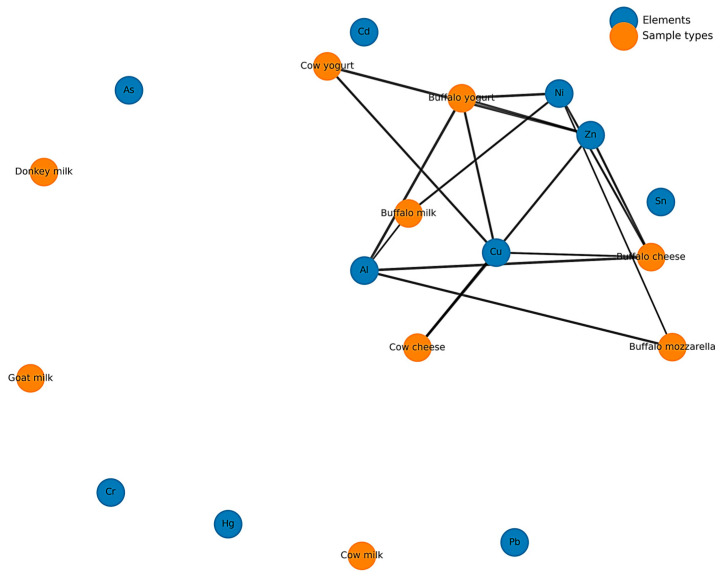
Bipartite correlation network linking elemental composition to dairy matrix types.

Figure 8 presents the association network between the analyzed dairy product types and the detected chemical elements. The topological analysis of the network indicates a significantly higher connectivity for buffalo-derived products (milk, yogurt, cheese, and mozzarella), which are associated with a larger number of elements, including Zn, Cu, Ni, and Al, forming a well-defined central cluster. This distribution indicates the concurrent presence of multiple elements within these dairy matrices.

Cow-derived products exhibit a lower degree of connectivity, with associations restricted to a limited subset of elements, while cow milk appears as an isolated node in the network. Similarly, goat milk and donkey milk are positioned at the periphery, reflecting the absence of detectable elemental associations according to the applied analytical criteria. The observed differences among product types can be attributed to variations in dairy matrix composition, differential elemental binding capacity, and the effects of technological processing, which influence element retention or loss.

Several elements, including As, Cd, Hg, Cr, Sn, and Pb, appear as isolated nodes, indicating that they were not associated with any of the analyzed dairy products. This lack of connectivity reflects concentrations below detection limits or values insufficient to meet the inclusion criteria of the network. Overall, the network structure illustrated in Figure 7 highlights systematic differences in elemental association patterns among dairy products, driven by raw material characteristics and processing-related factors.

The differences in elemental profiles among dairy matrices, animal species, and production systems can be attributed to a combination of biological, technological, and environmental factors. Species-specific metabolic characteristics influence the uptake and transfer of trace elements into milk, while variations in milk composition may further affect elemental partitioning. Differences between production systems are likely related to feeding practices, mineral supplementation, and herd management, which can contribute to variability in elemental content.

In addition, technological processing plays a role in shaping the elemental profiles of derived products. Fermentation, coagulation, and moisture removal during yogurt and cheese manufacture may lead to the relative concentration of certain elements. Regional environmental conditions, including soil, water, and pasture composition, may also indirectly influence the elemental composition through the feed chain. Together, these factors provide a coherent explanation for the variability observed across species, matrices, and production systems.

The consistently low concentrations of toxic elements observed across all analyzed dairy matrices, with values remaining below current European Union maximum limits, support the adequacy of existing production and control practices within the investigated supply chains. From a food safety perspective, these findings reinforce the suitability of milk and derived products from organic, conventional, and commercial systems for human consumption under the studied conditions. Moreover, the systematic detection of essential and trace elements within expected ranges highlights the value of multi-elemental profiling as a complementary tool for routine surveillance, capable of identifying deviations that may signal emerging contamination risks.

In addition, the differentiation of elemental patterns according to animal species, product type, and production system suggests a practical application for traceability. Elemental fingerprints generated by ICP–MS can contribute to product authentication, origin verification, and the monitoring of integrity along the “farm-to-retail” chain. Although not intended as a stand-alone traceability method, multi-elemental analysis may enhance existing documentation- and labeling-based systems by providing an objective, analytical layer of verification. In this context, the present results underline the potential of elemental profiling to support both regulatory oversight and quality assurance in dairy production.

The present investigation provides a comprehensive multi-elemental characterization of milk and selected dairy products; however, several contextual aspects should be acknowledged. The sampling framework was focused on two counties in north-western Romania and on a single collection period, which reflects the conditions of the study area and timeframe. Consequently, potential geographic and seasonal variability associated with environmental factors, feeding practices, and production systems may not be fully represented. In addition, although the overall number of samples was substantial, the representation of certain animal species and product categories was necessarily uneven.

Furthermore, the analytical approach was centered on the determination of elemental concentrations in final dairy matrices, without parallel assessment of environmental compartments such as soil, water, or feed, which could further elucidate sources and transfer pathways. While the sensitivity of ICP–MS ensured reliable quantification for most elements, some toxic metals were frequently below detection limits, restricting a more detailed evaluation of their distribution. Finally, consumer safety was assessed in relation to existing regulatory thresholds, without integrating dietary intake (with cumulative exposure from multiple foods or dairy matrices) or bioaccessibility parameters [6,39]. Study limitations are also related to geographic restrictions (north-western Romania), sampling period, and metal speciation (e.g., As and Cr). Within these boundaries, the study nevertheless offers robust baseline data and supports the applicability of multi-elemental analysis for monitoring dairy products across different production systems.

The fact that all measured concentrations of Pb, Cd, Hg, and As were below the maximum levels established by EU Regulation 2023/915 indicates a low dietary exposure to these toxic elements through the consumption of the investigated dairy products. From a consumer risk perspective, these results suggest that milk and dairy products from the studied production systems do not pose a significant health concern for the general population under normal consumption patterns. Although inter-species and matrix-related differences in elemental profiles were observed, these variations are unlikely to translate into meaningful differences in consumer risk. Nevertheless, continuous multi-elemental monitoring remains warranted, particularly for toxic metals, in order to promptly detect potential changes associated with environmental contamination or technological processing.

All samples were collected during a single sampling campaign conducted in October 2025. Although this approach ensured methodological consistency and minimized short-term variability, it does not capture potential seasonal fluctuations in feed composition and animal metabolism, which may influence trace-element transfer into milk. Therefore, the present results should be interpreted as representative of the autumn production period in north-western Romania. However, the investigated farms operated relatively stable feeding regimes, with extended grazing periods and controlled winter rations, which likely reduced abrupt dietary shifts at the time of sampling. Future studies incorporating multi-seasonal sampling are warranted to evaluate temporal variability and strengthen the generalizability of the findings.

The present study was geographically restricted to two counties in north-western Romania (Sălaj and Satu Mare), which represent predominantly rural areas with limited industrial activity. While this regional focus allowed for a controlled and coherent sampling design, it does not encompass the full ecological and industrial diversity of other Romanian regions. Therefore, the present findings should be interpreted as region-specific and may not be directly extrapolated to areas characterized by different environmental conditions or higher levels of industrial pollution. Future investigations, including broader geographic coverage and sites with contrasting ecological and industrial profiles, are warranted to strengthen the generalizability of the results.

The present investigation was designed primarily as a food safety and traceability study, with a specific focus on the multi-elemental composition of milk and dairy products and their compliance with current European Union regulatory limits. Routine nutritional quality indices, such as protein and fat content, were therefore outside the original analytical scope. Moreover, the determination of fat and protein would have required dedicated analytical platforms (e.g., Kjeldahl, Dumas, Gerber, or FTIR), distinct quality-control protocols, and separate method validation procedures, which were not available within the framework of the present ICP–MS–based study.

In addition, for several processed dairy products included in this work (commercial milk, yogurt, and cheese), fat and protein contents are technologically standardized during industrial processing, resulting in reduced biological variability and potentially limiting the interpretability of correlation analysis with trace-element concentrations. Finally, protein and fat levels in dairy matrices are strongly affected by technological steps such as degreasing, whey separation, and moisture removal, which could confound biological relationships between elemental composition and intrinsic milk quality. For these reasons, routine macronutrient profiling was not integrated into the present experimental design. Future studies combining multi-elemental analysis with compositional quality indices (protein, fat, lactose) are warranted to clarify potential interactions between elemental composition and dairy product quality.

Seasonal factors may influence elemental composition in milk through changes in feed composition, grazing intensity, and animal metabolism. In temperate regions, autumn sampling coincides with the transition from pasture-based diets to stored forages, which may modify the intake of certain trace elements. Although the farms included in this study operated relatively stable feeding regimes during the sampling period, the present results should be interpreted as representative of the autumn production season, and multi-seasonal sampling is warranted to assess temporal variability.

## 5. Conclusions

This study demonstrates a coherent elemental profile across all analyzed dairy matrices, in which toxic metals (Pb, Cd, Hg, and As) occur at very low levels, with Hg consistently below the limit of detection and Cd frequently non-detectable in processed products. In contrast, transition and essential elements (Cr, Ni, Al, Cu, Zn, and Sn) dominate the compositional patterns and reveal clear differentiation according to both animal species and processing stage. Buffalo-derived products generally exhibit higher concentrations of several elements, while technological processing leads to progressive enrichment, particularly for Cu and Zn, with the highest values observed in cheese. Comparisons between production systems show statistically significant differences between organic and commercial/retail products for multiple elements, with a general tendency toward higher concentrations in commercial samples, whereas organic products may present selective nutritional advantages for certain essential elements (e.g., Zn in milk). Overall, although all measured concentrations remain low and below regulatory limits for the elements subject to EU standards, the consistent patterns identified highlight the value of multi-element profiling by ICP–MS as a complementary tool for food safety monitoring, quality control, and product traceability, and underscore the need for continued surveillance of matrices and production chains in which elevated elemental levels may occur.

## Figures and Tables

**Table 1:** Mean Heavy Metal Concentrations (mg/kg) in Milk, Yogurt, and Cheese Products.

Sample Category/Product Type	Identification Code	Pb	Cd	Hg	As	Cr	Ni	Al	Sn	Cu	Zn
Organic cow milk	S1-MLK-001–S1-MLK-030	008	0.001	BLD	0.001	0.012	0.011	0.022	0.003	0.14	4.71
Goat milk (conventional)	S2-GOA-001C–S2-GOA-030C	0.006	0.001	–	0.001	0.012	0.011	0.022	0.003	0.13	4.51
Buffalo milk (conventional)	S2-BUF-001B–S2-BUF-040B	0.005	0.005	–	0.002	0.377	0.394	0.264	0.003	0.27	7.96
Organic cow yogurt	S1-YGT-031–S1-YGT-060	0.006	BLD	BLD	BLD	0.013	0.012	0.019	BLD	0.15	4.76
Commercial cow yogurt	Retail store samples	0.008	BLD	BLD	BLD	0.017	0.016	0.029	0.004	0.19	5.65
Buffalo yogurt	IB1–IB27	0.011	BLD	BLD	0.001	0.020	0.018	0.013	0.002	0.21	6.53
Buffalo milk cheese	S1-CHS-061–S1-CHS-090	0.007	BLD	BLD	0.001	0.015	0.012	0.021	0.003	0.18	6.02
Commercial cow cheese	Retail store samples	0.009	BLD	BLD	0.001	0.018	0.017	0.032	0.004	0.22	6.79
Buffalo mozzarella	BB1–BB30	0.008	BLD	BLD	0.001	0.019	0.018	0.035	0.004	0.24	7.24

Note: Mean concentrations (mg/kg) were calculated from the full analytical datasets reported in the Supplementary Materials. BLD = below limit of detection. Corresponding sample-level datasets and statistical summaries are provided as follows: organic cow milk (Table S13), conventional goat milk (Table S15), conventional buffalo milk (Table S17), organic cow yogurt (Table S23), commercial cow yogurt (Table S26), buffalo yogurt (Table S27), buffalo milk cheese (Table S29), commercial cow cheese (Table S32), and buffalo mozzarella (Table S33). Supplementary Tables S14, S16, S18, S20, S22, S24, S26, S30 and S34 contain descriptive statistics, left-censoring percentages, and distributional diagnostics.

**Table 2:** Comparison of metal concentrations (mg/kg) in cow milk from organic and commercial/retail systems (Mann–Whitney U test).

Element	Organic (Mean ± SD, *n*)	Commercial/Retail (Mean ± SD, *n*)	*p*-Value	Sig.
Pb	0.00809 ± 0.00182 (*n* = 30)	0.00667 ± 0.00200 (*n* = 9)	0.0642	ns
Cd	0.00106 ± 0.00027 (*n* = 30)	0.00186 ± 0.00048 (*n* = 5)	0.0022	**
Hg	BLD	BLD	-	
As	0.00106 ± 0.00023 (*n* = 30)	0.00425 ± 0.00167 (*n* = 8)	0.0000186	***
Cr	0.01199 ± 0.00169 (*n* = 30)	0.03315 ± 0.01273 (*n* = 20)	<0.0000001	***
Ni	0.01121 ± 0.00153 (*n* = 30)	0.02760 ± 0.01035 (*n* = 20)	<0.0000001	***
Al	0.02207 ± 0.00299 (*n* = 30)	0.12260 ± 0.04184 (*n* = 20)	<0.0000001	***
Cu	0.13917 ± 0.01397 (*n* = 30)	0.14635 ± 0.04090 (*n* = 20)	0.5657	ns
Zn	4.71900 ± 0.29595 (*n* = 30)	0.46810 ± 0.09345 (*n* = 20)	<0.0000001	***
Sn	0.00349 ± 0.00038 (*n* = 30)	0.01305 ± 0.00633 (*n* = 20)	<0.0000001	***

Values are expressed as mean ± standard deviation (SD); n represents the number of samples analyzed. Concentrations are reported in mg/kg (wet weight). Statistical differences between production systems (organic vs. commercial/retail) were assessed using the Mann–Whitney U test. Significance levels are indicated as: ns = not significant (*p* ≥ 0.05); ** *p* < 0.01; *** *p* < 0.001. BLD = below the limit of detection; for elements with values entirely below detection (e.g., Hg), no statistical comparison was performed. Extremely small *p*-values are reported in numeric decimal format or as threshold values (e.g., *p* < 0.0000001) to avoid scientific notation.

**Table 3:** Comparison of metal concentrations (mg/kg) in cow yogurt from organic and commercial/retail systems (Mann–Whitney U test).

Element	Organic (Mean ± SD, *n*)	Commercial/Retail (Mean ± SD, *n*)	*p*-Value	Sig.
Pb	0.00550 ± 0.00160 (*n* = 30)	0.00846 ± 0.00285 (*n* = 13)	0.0084	**
Cd	BLD	BLD	-	
Hg	BLD	BLD	-	
As	BLD	0.00360 ± 0.00099 (*n* = 4)	<0.0000001	***
Cr	0.01270 ± 0.00217 (*n* = 30)	0.01765 ± 0.00379 (*n* = 20)	0.0000593	***
Ni	0.01174 ± 0.00196 (*n* = 30)	0.01805 ± 0.00341 (*n* = 20)	<0.0000001	***
Al	0.02850 ± 0.00305 (*n* = 30)	0.04195 ± 0.00662 (*n* = 20)	0.0658	ns
Cu	0.14667 ± 0.02098 (*n* = 30)	0.19350 ± 0.02706 (*n* = 20)	<0.0000001	***
Zn	4.76100 ± 0.39472 (*n* = 30)	5.65350 ± 0.46843 (*n* = 20)	<0.0000001	***
Sn	0.00330 ± 0.00000 (*n* = 30)	0.00375 ± 0.00071 (*n* = 20)	0.0660	ns

Values are expressed as mean ± standard deviation (SD); *n* represents the number of analyzed samples. Concentrations are reported in mg/kg (wet weight). Statistical differences between production systems (organic vs. commercial/retail) were assessed using the Mann–Whitney U test. Significance levels are indicated as: ns = not significant (*p* ≥ 0.05); ** *p* < 0.01; *** *p* < 0.001. BLD = below the limit of detection; for elements with values entirely below detection (e.g., Cd and Hg), no statistical comparison was performed. Extremely small *p*-values are reported in numeric decimal format or as threshold values (e.g., *p* < 0.0000001) to avoid scientific notation.

**Table 4:** Comparison of metal concentrations (mg/kg) in cow cheese from organic and commercial/retail systems (Mann–Whitney U test).

Element	Organic (Mean ± SD, *n*)	Commercial/Retail (Mean ± SD, *n*)	*p*-Value	Sig.
Pb	0.00727 ± 0.00210 (*n* = 30)	0.00946 ± 0.00290 (*n* = 13)	0.0340	*
Cd	BLD	BLD	-	
Hg	BLD	BLD	-	
As	0.00073 ± 0.00019 (*n* = 30)	0.00150 ± 0.00071 (*n* = 20)	0.1700	ns
Cr	0.01093 ± 0.00210 (*n* = 30)	0.01775 ± 0.00390 (*n* = 20)	0.000102	***
Ni	0.01053 ± 0.00194 (*n* = 30)	0.01820 ± 0.00365 (*n* = 20)	0.000111	***
Al	0.03200 ± 0.00604 (*n* = 30)	0.04975 ± 0.01493 (*n* = 20)	0.0000004	***
Cu	0.15900 ± 0.02331 (*n* = 30)	0.21650 ± 0.03329 (*n* = 20)	0.000294	***
Zn	6.42170 ± 0.65796 (*n* = 30)	6.78500 ± 0.47961 (*n* = 20)	0.000406	***
Sn	0.00343 ± 0.00067 (*n* = 30)	0.00375 ± 0.00071 (*n* = 8)	0.1090	ns

Values are expressed as mean ± standard deviation (SD); *n* represents the number of analyzed samples. Concentrations are reported in mg/kg (wet weight). Statistical differences between production systems (organic vs. commercial/retail) were assessed using the Mann-Whitney U test. Significance levels are indicated as: ns = not significant (*p* ≥ 0.05); * *p* < 0.05; *** *p* < 0.001. BLD = below the limit of detection; for elements with values entirely below detection (e.g., Cd and Hg), no statistical comparison was performed. Extremely small *p*-values are reported in numeric decimal format or as threshold values (e.g., *p* < 0.0000001) to avoid scientific notation.

**Table 5:** Effect of Processing on Metal Concentrations (mg/kg) in Dairy Products (Milk, Yogurt, and Cheese) Based on the Mann–Whitney U Test.

Element	Milk (Mean)	Yogurt (Mean)	Cheese (Mean)	*p*-Value (Processing)	Significance	Trend with Processing
Pb	0.00809	0.00550	0.00727	0.0340 (Milk vs. Cheese)	*	Decrease in yogurt, then increase
Cd	0.00106	BLD	BLD	-	-	Decreases/not detected
Hg	BLD	BLD	BLD	-	-	Not detection
As	0.00106	BLD	0.00073	0.1700 (Milk vs. Cheese)	ns	Decrease after processing
Cr	0.01199	0.01270	0.01093	0.000102 (Milk vs. Cheese)	***	Relatively stable
Ni	0.01121	0.01174	0.01053	0.000111 (Milk vs. Cheese)	***	Relatively stable
Al	0.02207	0.02850	0.03200	0.0000004 (Milk vs. Cheese)	***	Increases with processing
Cu	0.13917	0.14667	0.15900	0.000294 (Milk vs. Cheese)	***	Gradual increase
Zn	4.71900	4.76100	6.42170	0.000406 (Milk vs. Cheese)	***	Strong increase in cheese
Sn	0.00349	0.00330	0.00343	0.1090 (Milk vs. Cheese)	ns	Stable

Values represent mean metal concentrations (mg/kg, wet weight). BLD indicates concentrations below the analytical limit of detection. Where all measurements were BLD, statistical testing was not applicable. *p*-values were obtained using the Mann–Whitney U test and represent comparisons between milk and cheese to assess the effect of processing. Significance levels are defined as: ns (*p* ≥ 0.05), * (*p* < 0.05), *** (*p* < 0.001). The processing trend summarizes the direction of change across milk, yogurt, and cheese based on mean values.

**Table 6:** Effect of Processing on Metal Concentrations (mg/kg) in Commercial/Retail Dairy Products (Milk, Yogurt, and Cheese) Based on the Mann–Whitney U Test.

Element	Milk (Mean)	Yogurt (Mean)	Cheese (Mean)	*p*-Value (Processing)	Significance	Trend with Processing
Pb	0.00667	0.00846	0.00946	0.0340 (Milk vs. Cheese)	*	Increases with processing
Cd	0.00186	BLD	BLD	-	-	Decreases/not detected
Hg	BLD	BLD	BLD	-	-	Not detection
As	0.00425	0.00360	0.00150	0.1700 (Milk vs. Cheese)	ns	Decreases with processing
Cr	0.03315	0.01765	0.01775	0.000102 (Milk vs. Cheese)	***	Decreases after milk
Ni	0.02760	0.01805	0.01820	0.000111 (Milk vs. Cheese)	***	Decreases after milk
Al	0.12260	0.04195	0.04975	0.0000004 (Milk vs. Cheese)	***	Decreases after milk, slight increase in cheese
Cu	0.14635	0.19350	0.21650	0.000294 (Milk vs. Cheese)	***	Increases with processing
Zn	0.46810	5.65350	6.78500	0.000406 (Milk vs. Cheese)	***	Very strong increase
Sn	0.01305	0.00375	0.00375	0.1090 (Milk vs. Cheese)	ns	Decreases after milk

All values represent mean concentrations (mg/kg, wet weight). BLD denotes concentrations below the analytical limit of detection. Where measurements were BLD, statistical testing was not applicable. *p*-values were obtained using the Mann–Whitney U test and correspond to milk-cheese comparisons to evaluate the effect of processing. Significance is denoted as ns (*p* ≥ 0.05), * (*p* < 0.05), and *** (*p* < 0.001). The processing trend reflects the qualitative change across milk, yogurt, and cheese based on mean values.

## Data Availability

The data presented in this study is available on request from the corresponding author. The data are not publicly available due to their inclusion in ongoing analyses and supplementary datasets.

## Supplementary Materials

The following supporting information can be downloaded at: https://www.mdpi.com/article/10.3390/toxics14020124/s1, Table S1. Identification and Metadata of Cow Milk Samples Collected from an Organic Dairy Farm in North-western Romania (Sălaj County); Table S2. Identification and Metadata of Cow Yogurt Samples Collected from an Organic Dairy Farm in North-western Romania (Sălaj County); Table S3. Identification and Metadata of Cow Cheese Samples Collected from an Organic Dairy Farm in North-western Romania (Sălaj County); Table S4. Identification and Metadata of Goat Milk Samples Collected from a Goat Farm in North-western Romania (Satu Mare County); Table S5. Identification and Metadata of Buffalo Milk Samples Collected from a Buffalo Farm in North-western Romania (Satu Mare County); Table S6. Identification and Metadata of Donkey Milk Samples Collected from a Donkey Farm in North-western Romania (Satu Mare County); Table S7. Identification and Metadata of Retail Conventional Cow Dairy Products and Buffalo Farm-Origin Dairy Products Analyzed in This Study; Table S8. Microwave digestion conditions for dairy matrices using Milestone START D; Table S9. The instrumental settings (a) and data acquisition parameters (b) of the ICP-MS system, which define the operating conditions and analytical procedures for precise metal quantification; Table S10. Calibration Levels and Instrumental Parameters for ICP–MS Determination of Trace Metals in Dairy Matrices; Table S11. CRM Performance and Quality Control Evaluation for ICP–MS Quantification of Trace Metals in Dairy Matrices; Table S12. Verification of Analytical Accuracy Using FAPAS Whole Milk Quality Control Material and Z-Score Interpretation; Table S13. Analytical dataset of heavy metal concentrations (mg/kg) in organic cow milk samples (*n* = 30); Table S14. Comprehensive statistical evaluation (descriptive, dispersion, distribution and censoring indices) of heavy metal concentrations (mg/kg) in organic cow milk samples (*n* = 30); Table S15. Analytical dataset of heavy metal concentrations (mg/kg) in conventional goat milk samples (*n* = 30); Table S16. Comprehensive statistical evaluation (descriptive, dispersion, distribution and censoring indices) of heavy metal concentrations (mg/kg) in goat milk samples (*n* = 30); Table S17. Analytical dataset of heavy metal concentrations (mg/kg) in conventional buffalo milk samples (*n* = 40); Table S18. Comprehensive statistical evaluation (descriptive, dispersion, distribution and censoring indices) of heavy metal concentrations (mg/kg) in buffalo milk samples (*n* = 40); Table S19. Analytical dataset of heavy metal concentrations (mg/kg) in conventional donkey milk samples (*n* = 30); Table S20. Comprehensive statistical evaluation (descriptive, dispersion, distribution and censoring indices) of heavy metal concentrations (mg/kg) in donkey milk samples (*n* = 30); Table S21. Analytical dataset of heavy metal concentrations (mg/kg) in conventional cow milk samples from a retail store (*n* = 20); Table S22. Comprehensive statistical evaluation (descriptive, dispersion, distribution and censoring indices) of heavy metal concentrations (mg/kg) in cow milk samples from a retail store (*n* = 20); Table S23. Analytical dataset of heavy metal concentrations (mg/kg) in cow yogurt samples produced from organic cow milk (*n* = 30); Table S24. Comprehensive statistical evaluation (descriptive, dispersion, distribution and censoring indices) of heavy metal concentrations (mg/kg) in cow yogurt samples produced from organic cow milk (*n* = 30); Table S25. Analytical dataset of heavy metal concentrations (mg/kg) commercial yogurt samples produced from cow’s milk and purchased from retail stores (*n* = 30); Table S26. Comprehensive statistical evaluation (descriptive, dispersion, distribution and censoring indices) of heavy metal concentrations (mg/kg) in commercial yogurt samples produced from cow’s milk and purchased from retail stores (*n* = 20); Table S27. Analytical dataset of heavy metal concentrations (mg/kg) yogurt samples produced from buffalo milk (*n* = 27); Table S28. Comprehensive statistical evaluation (descriptive, dispersion, distribution and censoring indices) of heavy metal concentrations (mg/kg) in buffalo yogurt samples produced from buffalo milk (*n* = 27); Table S29. Analytical dataset of heavy metal concentrations (mg/kg) in cheese samples produced from buffalo milk (*n* = 30); Table S30. Comprehensive statistical evaluation (descriptive, dispersion, distribution and censoring indices) of heavy metal concentrations (mg/kg) in cheese samples produced from buffalo milk (*n* = 30); Table S31. Analytical dataset of heavy metal concentrations (mg/kg) commercial cheese samples produced from cow’s milk and purchased from retail stores (*n* = 30); Table S32. Comprehensive statistical evaluation (descriptive, dispersion, distribution and censoring indices) of heavy metal concentrations (mg/kg) in commercial cheese samples produced from cow’s milk and purchased from retail stores (*n* = 20); Table S33. Analytical dataset of heavy metal concentrations (mg/kg) Mozzarella cheese samples produced from buffalo milk (*n* = 30); Table S34. Comprehensive statistical evaluation (descriptive, dispersion, distribution and censoring indices) of heavy metal concentrations (mg/kg) in mozzarella cheese samples produced from buffalo milk (*n* = 30); Table S35. Maximum Permissible Levels of Metals in Milk and Dairy Products (EU); Text S1. Methodology.

## Abbreviations

The following abbreviations are used in this manuscript:

ICP–MS	Inductively Coupled Plasma Mass Spectrometry
He/KED	Helium Collision Mode with Kinetic Energy Discrimination
CRM	Certified Reference Material
NIST	National Institute of Standards and Technology
FAPAS	Food Analysis Performance Assessment Scheme
BLD	Below Limit of Detection
LOQ	Limit of Quantification
LCD	Left-Censored Data (percentage of values below LOQ)
SD	Standard Deviation
SE	Standard Error
RSD	Relative Standard Deviation
CV	Coefficient of Variation
CI95%	95% Confidence Interval
GPS	Global Positioning System
HTST	High-Temperature Short-Time (pasteurization)
LTLT	Low-Temperature Long-Time (pasteurization)
EU	European Union
ISO	International Organization for Standardization
AOAC	Association of Official Analytical Chemists
IUPAC	International Union of Pure and Applied Chemistry
QC	Quality Control
RF	Radio Frequency
m/z	Mass-to-Charge Ratio
