# Supplementary material for: From Farm to Retail: Decoding the Elemental Landscape of Milk and Dairy Products Across Organic and Conventional Production Systems Using ICP–MS
Source: Toxics. 2026 Jan 28;14(2):124. doi: 10.3390/toxics14020124 (PMC12944908; doi:10.3390/toxics14020124)
Supplement: Supplementary file 1 [file toxics-14-00124-s001.zip › toxics-4117193-supplementary.pdf]

Table S1. Identification and Metadata of Cow Milk Samples Collected from an Organic Dairy Farm in North-western Romania (Sălaj County)

Identification Code	Set	Sample No.	Sample Type	Source	Animal	Farm	Sample ID	Collection Date	Locality	Internal batch code	Latitude (°N)	Longitude (°E)
S1-MLK-001	Set 1	1	Milk	Dairy	Cow	Organic dairy farm	MSR2025-00001	05.10.2025	Sălaj County	457101	47.23	23.08
S1-MLK-002	Set 1	2	Milk	Dairy	Cow	Organic dairy farm	MSR2025-00002	05.10.2025	Sălaj County	457102	47.23	23.08
S1-MLK-003	Set 1	3	Milk	Dairy	Cow	Organic dairy farm	MSR2025-00003	05.10.2025	Sălaj County	457103	47.23	23.08
S1-MLK-004	Set 1	4	Milk	Dairy	Cow	Organic dairy farm	MSR2025-00004	05.10.2025	Sălaj County	457104	47.23	23.08
S1-MLK-005	Set 1	5	Milk	Dairy	Cow	Organic dairy farm	MSR2025-00005	05.10.2025	Sălaj County	457105	47.23	23.08
S1-MLK-006	Set 1	6	Milk	Dairy	Cow	Organic dairy farm	MSR2025-00006	05.10.2025	Sălaj County	457106	47.23	23.08
S1-MLK-007	Set 1	7	Milk	Dairy	Cow	Organic dairy farm	MSR2025-00007	05.10.2025	Sălaj County	457107	47.23	23.08
S1-MLK-008	Set 1	8	Milk	Dairy	Cow	Organic dairy farm	MSR2025-00008	05.10.2025	Sălaj County	457108	47.23	23.08
S1-MLK-009	Set 1	9	Milk	Dairy	Cow	Organic dairy farm	MSR2025-00009	05.10.2025	Sălaj County	457109	47.23	23.08
S1-MLK-010	Set 1	10	Milk	Dairy	Cow	Organic dairy farm	MSR2025-00010	05.10.2025	Sălaj County	457110	47.23	23.08
S1-MLK-011	Set 1	11	Milk	Dairy	Cow	Organic dairy farm	MSR2025-00011	05.10.2025	Sălaj County	457111	47.23	23.08
S1-MLK-012	Set 1	12	Milk	Dairy	Cow	Organic dairy farm	MSR2025-00012	05.10.2025	Sălaj County	457112	47.23	23.08
S1-MLK-013	Set 1	13	Milk	Dairy	Cow	Organic dairy farm	MSR2025-00013	05.10.2025	Sălaj County	457113	47.23	23.08
S1-MLK-014	Set 1	14	Milk	Dairy	Cow	Organic dairy farm	MSR2025-00014	05.10.2025	Sălaj County	457114	47.23	23.08
S1-MLK-015	Set 1	15	Milk	Dairy	Cow	Organic dairy farm	MSR2025-00015	05.10.2025	Sălaj County	457115	47.23	23.08
S1-MLK-016	Set 1	16	Milk	Dairy	Cow	Organic dairy farm	MSR2025-00016	05.10.2025	Sălaj County	457116	47.23	23.08
S1-MLK-017	Set 1	17	Milk	Dairy	Cow	Organic dairy farm	MSR2025-00017	05.10.2025	Sălaj County	457117	47.23	23.08
S1-MLK-018	Set 1	18	Milk	Dairy	Cow	Organic dairy farm	MSR2025-00018	05.10.2025	Sălaj County	457118	47.23	23.08
S1-MLK-019	Set 1	19	Milk	Dairy	Cow	Organic dairy farm	MSR2025-00019	05.10.2025	Sălaj County	457119	47.23	23.08
S1-MLK-020	Set 1	20	Milk	Dairy	Cow	Organic dairy farm	MSR2025-00020	05.10.2025	Sălaj County	457120	47.23	23.08
S1-MLK-021	Set 1	21	Milk	Dairy	Cow	Organic dairy farm	MSR2025-00021	05.10.2025	Sălaj County	457121	47.23	23.08
S1-MLK-022	Set 1	22	Milk	Dairy	Cow	Organic dairy farm	MSR2025-00022	05.10.2025	Sălaj County	457122	47.23	23.08
S1-MLK-023	Set 1	23	Milk	Dairy	Cow	Organic dairy farm	MSR2025-00023	05.10.2025	Sălaj County	457123	47.23	23.08
S1-MLK-024	Set 1	24	Milk	Dairy	Cow	Organic dairy farm	MSR2025-00024	05.10.2025	Sălaj County	457124	47.23	23.08
S1-MLK-025	Set 1	25	Milk	Dairy	Cow	Organic dairy farm	MSR2025-00025	05.10.2025	Sălaj County	457125	47.23	23.08

S1-MLK-026	Set 1	26	Milk	Dairy	Cow	Organic dairy farm	MSR2025-00026	05.10.2025	Sălaj County	457126	47.23	23.08
S1-MLK-027	Set 1	27	Milk	Dairy	Cow	Organic dairy farm	MSR2025-00027	05.10.2025	Sălaj County	457127	47.23	23.08
S1-MLK-028	Set 1	28	Milk	Dairy	Cow	Organic dairy farm	MSR2025-00028	05.10.2025	Sălaj County	457128	47.23	23.08
S1-MLK-029	Set 1	29	Milk	Dairy	Cow	Organic dairy farm	MSR2025-00029	05.10.2025	Sălaj County	457129	47.23	23.08
S1-MLK-030	Set 1	30	Milk	Dairy	Cow	Organic dairy farm	MSR2025-00030	05.10.2025	Sălaj County	457130	47.23	23.08

All milk samples (S1-MLK-001 to S1-MLK-030) were collected on October 5th, 2025, from an organic dairy farm in north-western Romania. Each sample represents cow milk obtained under hygienic conditions during the morning milking. Immediately after collection, the samples were cooled and stored in stainless steel tanks at 2–4 °C to inhibit microbial activity and preserve the physicochemical characteristics of the milk until laboratory analysis. The identification codes (MSR2025-00001 to MSR2025-00030) correspond to the internal traceability and sample management system established for the monitoring campaign. For confidentiality reasons, the farm name and exact geographic location have been anonymized, and coordinates are reported in rounded form.

Table S2. Identification and Metadata of Cow Yogurt Samples Collected from an Organic Dairy Farm in North-western Romania (Sălaj County)

Identification Code	Set	Sample No.	Sample Type	Source	Animal	Farm	Sample ID	Collection Date	Locality	Internal batch code	Latitude (°N)	Longitude (°E)

S1-YGT-031	Set 2	31	Yogurt	Dairy	Cow	Organic dairy farm	MSR2025-00031	05.10.2025	Sălaj County	457131	47.23	23.08
S1-YGT-032	Set 2	32	Yogurt	Dairy	Cow	Organic dairy farm	MSR2025-00032	05.10.2025	Sălaj County	457132	47.23	23.08
S1-YGT-033	Set 2	33	Yogurt	Dairy	Cow	Organic dairy farm	MSR2025-00033	05.10.2025	Sălaj County	457133	47.23	23.08
S1-YGT-034	Set 2	34	Yogurt	Dairy	Cow	Organic dairy farm	MSR2025-00034	05.10.2025	Sălaj County	457134	47.23	23.08
S1-YGT-035	Set 2	35	Yogurt	Dairy	Cow	Organic dairy farm	MSR2025-00035	05.10.2025	Sălaj County	457135	47.23	23.08
S1-YGT-036	Set 2	36	Yogurt	Dairy	Cow	Organic dairy farm	MSR2025-00036	05.10.2025	Sălaj County	457136	47.23	23.08
S1-YGT-037	Set 2	37	Yogurt	Dairy	Cow	Organic dairy farm	MSR2025-00037	05.10.2025	Sălaj County	457137	47.23	23.08
S1-YGT-038	Set 2	38	Yogurt	Dairy	Cow	Organic dairy farm	MSR2025-00038	05.10.2025	Sălaj County	457138	47.23	23.08
S1-YGT-039	Set 2	39	Yogurt	Dairy	Cow	Organic dairy farm	MSR2025-00039	05.10.2025	Sălaj County	457139	47.23	23.08
S1-YGT-040	Set 2	40	Yogurt	Dairy	Cow	Organic dairy farm	MSR2025-00040	05.10.2025	Sălaj County	457140	47.23	23.08
S1-YGT-041	Set 2	41	Yogurt	Dairy	Cow	Organic dairy farm	MSR2025-00041	05.10.2025	Sălaj County	457141	47.23	23.08
S1-YGT-042	Set 2	42	Yogurt	Dairy	Cow	Organic dairy farm	MSR2025-00042	05.10.2025	Sălaj County	457142	47.23	23.08
S1-YGT-043	Set 2	43	Yogurt	Dairy	Cow	Organic dairy farm	MSR2025-00043	05.10.2025	Sălaj County	457143	47.23	23.08
S1-YGT-044	Set 2	44	Yogurt	Dairy	Cow	Organic dairy farm	MSR2025-00044	05.10.2025	Sălaj County	457144	47.23	23.08
S1-YGT-045	Set 2	45	Yogurt	Dairy	Cow	Organic dairy farm	MSR2025-00045	05.10.2025	Sălaj County	457145	47.23	23.08
S1-YGT-046	Set 2	46	Yogurt	Dairy	Cow	Organic dairy farm	MSR2025-00046	05.10.2025	Sălaj County	457146	47.23	23.08
S1-YGT-047	Set 2	47	Yogurt	Dairy	Cow	Organic dairy farm	MSR2025-00047	05.10.2025	Sălaj County	457147	47.23	23.08
S1-YGT-048	Set 2	48	Yogurt	Dairy	Cow	Organic dairy farm	MSR2025-00048	05.10.2025	Sălaj County	457148	47.23	23.08
S1-YGT-049	Set 2	49	Yogurt	Dairy	Cow	Organic dairy farm	MSR2025-00049	05.10.2025	Sălaj County	457149	47.23	23.08
S1-YGT-050	Set 2	50	Yogurt	Dairy	Cow	Organic dairy farm	MSR2025-00050	05.10.2025	Sălaj County	457150	47.23	23.08
S1-YGT-051	Set 2	51	Yogurt	Dairy	Cow	Organic dairy farm	MSR2025-00051	05.10.2025	Sălaj County	457151	47.23	23.08
S1-YGT-052	Set 2	52	Yogurt	Dairy	Cow	Organic dairy farm	MSR2025-00052	05.10.2025	Sălaj County	457152	47.23	23.08
S1-YGT-053	Set 2	53	Yogurt	Dairy	Cow	Organic dairy farm	MSR2025-00053	05.10.2025	Sălaj County	457153	47.23	23.08
S1-YGT-054	Set 2	54	Yogurt	Dairy	Cow	Organic dairy farm	MSR2025-00054	05.10.2025	Sălaj County	457154	47.23	23.08
S1-YGT-055	Set 2	55	Yogurt	Dairy	Cow	Organic dairy farm	MSR2025-00055	05.10.2025	Sălaj County	457155	47.23	23.08
S1-YGT-056	Set 2	56	Yogurt	Dairy	Cow	Organic dairy farm	MSR2025-00056	05.10.2025	Sălaj County	457156	47.23	23.08
S1-YGT-057	Set 2	57	Yogurt	Dairy	Cow	Organic dairy farm	MSR2025-00057	05.10.2025	Sălaj County	457157	47.23	23.08
S1-YGT-058	Set 2	58	Yogurt	Dairy	Cow	Organic dairy farm	MSR2025-00058	05.10.2025	Sălaj County	457158	47.23	23.08

S1-YGT-059	Set 2	59	Yogurt	Dairy	Cow	Organic dairy farm	MSR2025-00059	05.10.2025	Sălaj County	457159	47.23	23.08
S1-YGT-060	Set 2	60	Yogurt	Dairy	Cow	Organic dairy farm	MSR2025-00060	05.10.2025	Sălaj County	457160	47.23	23.08

All yogurt samples (IDs S1-YGT-031 to S1-YGT-060) were produced from cow's milk sourced at an organic dairy farm in north-western Romania (Sălaj County) on 5 October 2025. Milk was pasteurized and fermented under hygienic conditions according to the producer's standard process. After processing, the yogurt was dispensed into retail containers, immediately cooled, and held at 2–4 °C until sampling. Each unit was labeled using the internal traceability codes MSR2025-00031 to MSR2025-00060. Samples were transported to the laboratory on the same day in insulated containers (2–4 °C) and logged upon receipt for analysis.

Table S3. Identification and Metadata of Cow Cheese Samples Collected from an Organic Dairy Farm in North-western Romania (Sălaj County)

Identification Code	Set	Sample No.	Sample Type	Source	Animal	Farm	Sample ID	Collection Date	Locality	Internal batch code	Latitude (°N)	Longitude (°E)
S1-CHS-061	Set 3	61	Cheese	Dairy	Cow	Organic dairy farm	MSR2025-00061	05.10.2025	Sălaj County	457161	47.23	23.08

S1-CHS-062	Set 3	62	Cheese	Dairy	Cow	Organic dairy farm	MSR2025-00062	05.10.2025	Sălaj County	457162	47.23	23.08
S1-CHS-063	Set 3	63	Cheese	Dairy	Cow	Organic dairy farm	MSR2025-00063	05.10.2025	Sălaj County	457163	47.23	23.08
S1-CHS-064	Set 3	64	Cheese	Dairy	Cow	Organic dairy farm	MSR2025-00064	05.10.2025	Sălaj County	457164	47.23	23.08
S1-CHS-065	Set 3	65	Cheese	Dairy	Cow	Organic dairy farm	MSR2025-00065	05.10.2025	Sălaj County	457165	47.23	23.08
S1-CHS-066	Set 3	66	Cheese	Dairy	Cow	Organic dairy farm	MSR2025-00066	05.10.2025	Sălaj County	457166	47.23	23.08
S1-CHS-067	Set 3	67	Cheese	Dairy	Cow	Organic dairy farm	MSR2025-00067	05.10.2025	Sălaj County	457167	47.23	23.08
S1-CHS-068	Set 3	68	Cheese	Dairy	Cow	Organic dairy farm	MSR2025-00068	05.10.2025	Sălaj County	457168	47.23	23.08
S1-CHS-069	Set 3	69	Cheese	Dairy	Cow	Organic dairy farm	MSR2025-00069	05.10.2025	Sălaj County	457169	47.23	23.08
S1-CHS-070	Set 3	70	Cheese	Dairy	Cow	Organic dairy farm	MSR2025-00070	05.10.2025	Sălaj County	457170	47.23	23.08
S1-CHS-071	Set 3	71	Cheese	Dairy	Cow	Organic dairy farm	MSR2025-00071	05.10.2025	Sălaj County	457171	47.23	23.08
S1-CHS-072	Set 3	72	Cheese	Dairy	Cow	Organic dairy farm	MSR2025-00072	05.10.2025	Sălaj County	457172	47.23	23.08
S1-CHS-073	Set 3	73	Cheese	Dairy	Cow	Organic dairy farm	MSR2025-00073	05.10.2025	Sălaj County	457173	47.23	23.08
S1-CHS-074	Set 3	74	Cheese	Dairy	Cow	Organic dairy farm	MSR2025-00074	05.10.2025	Sălaj County	457174	47.23	23.08
S1-CHS-075	Set 3	75	Cheese	Dairy	Cow	Organic dairy farm	MSR2025-00075	05.10.2025	Sălaj County	457175	47.23	23.08
S1-CHS-076	Set 3	76	Cheese	Dairy	Cow	Organic dairy farm	MSR2025-00076	05.10.2025	Sălaj County	457176	47.23	23.08
S1-CHS-077	Set 3	77	Cheese	Dairy	Cow	Organic dairy farm	MSR2025-00077	05.10.2025	Sălaj County	457177	47.23	23.08
S1-CHS-078	Set 3	78	Cheese	Dairy	Cow	Organic dairy farm	MSR2025-00078	05.10.2025	Sălaj County	457178	47.23	23.08
S1-CHS-079	Set 3	79	Cheese	Dairy	Cow	Organic dairy farm	MSR2025-00079	05.10.2025	Sălaj County	457179	47.23	23.08
S1-CHS-080	Set 3	80	Cheese	Dairy	Cow	Organic dairy farm	MSR2025-00080	05.10.2025	Sălaj County	457180	47.23	23.08
S1-CHS-081	Set 3	81	Cheese	Dairy	Cow	Organic dairy farm	MSR2025-00081	05.10.2025	Sălaj County	457181	47.23	23.08
S1-CHS-082	Set 3	82	Cheese	Dairy	Cow	Organic dairy farm	MSR2025-00082	05.10.2025	Sălaj County	457182	47.23	23.08
S1-CHS-083	Set 3	83	Cheese	Dairy	Cow	Organic dairy farm	MSR2025-00083	05.10.2025	Sălaj County	457183	47.23	23.08
S1-CHS-084	Set 3	84	Cheese	Dairy	Cow	Organic dairy farm	MSR2025-00084	05.10.2025	Sălaj County	457184	47.23	23.08
S1-CHS-085	Set 3	85	Cheese	Dairy	Cow	Organic dairy farm	MSR2025-00085	05.10.2025	Sălaj County	457185	47.23	23.08
S1-CHS-086	Set 3	86	Cheese	Dairy	Cow	Organic dairy farm	MSR2025-00086	05.10.2025	Sălaj County	457186	47.23	23.08
S1-CHS-087	Set 3	87	Cheese	Dairy	Cow	Organic dairy farm	MSR2025-00087	05.10.2025	Sălaj County	457187	47.23	23.08
S1-CHS-088	Set 3	88	Cheese	Dairy	Cow	Organic dairy farm	MSR2025-00088	05.10.2025	Sălaj County	457188	47.23	23.08
S1-CHS-089	Set 3	89	Cheese	Dairy	Cow	Organic dairy farm	MSR2025-00089	05.10.2025	Sălaj County	457189	47.23	23.08
S1-CHS-090	Set 3	90	Cheese	Dairy	Cow	Organic dairy farm	MSR2025-00090	05.10.2025	Sălaj County	457190	47.23	23.08

All cheese samples (codes S1-CHS-061 to S1-CHS-090) were obtained from cow's milk processed at an organic dairy farm in north-western Romania (Sălaj County) on 05 October 2025. Milk was pasteurized and coagulated under controlled hygienic conditions according to the producer's standard procedure. The resulting cheese was matured as required, then packaged in food-grade containers following processing and kept at a constant 2–4 °C until sampling. Each sample was individually trace-coded (MSR2025-00061 to MSR2025-00090) within the internal tracking system. All units were collected on the production date and transported under refrigeration (2–4 °C) for subsequent laboratory analysis. The numeric identifiers 457161–457190 represent internal batch/location codes used for traceability and do not correspond to distinct geographic sampling sites.

Table S4. Identification and Metadata of Goat Milk Samples Collected from a Goat Farm in North-western Romania (Satu Mare County)

Identification Code	Set	Sample No.	Sample Type	Source	Animal	Farm	Sample ID	Collection Date	Locality	Latitude (°N)	Longitude (°E)
S2-GOA-001C	Set 4	91	Milk	Dairy	Goat	Goat farm	MSR2025-00091	24.10.2025	Satu Mare County	47.93	23.45
S2-GOA-002C	Set 4	92	Milk	Dairy	Goat	Goat farm	MSR2025-00092	24.10.2025	Satu Mare County	47.93	23.45
S2-GOA-003C	Set 4	93	Milk	Dairy	Goat	Goat farm	MSR2025-00093	24.10.2025	Satu Mare County	47.93	23.45

S2-GOA-004C	Set 4	94	Milk	Dairy	Goat	Goat farm	MSR2025-00094	24.10.2025	Satu Mare County	47.93	23.45
S2-GOA-005C	Set 4	95	Milk	Dairy	Goat	Goat farm	MSR2025-00095	24.10.2025	Satu Mare County	47.93	23.45
S2-GOA-006C	Set 4	96	Milk	Dairy	Goat	Goat farm	MSR2025-00096	24.10.2025	Satu Mare County	47.93	23.45
S2-GOA-007C	Set 4	97	Milk	Dairy	Goat	Goat farm	MSR2025-00097	24.10.2025	Satu Mare County	47.93	23.45
S2-GOA-008C	Set 4	98	Milk	Dairy	Goat	Goat farm	MSR2025-00098	24.10.2025	Satu Mare County	47.93	23.45
S2-GOA-009C	Set 4	99	Milk	Dairy	Goat	Goat farm	MSR2025-00099	24.10.2025	Satu Mare County	47.93	23.45
S2-GOA-010C	Set 4	100	Milk	Dairy	Goat	Goat farm	MSR2025-00100	24.10.2025	Satu Mare County	47.93	23.45
S2-GOA-011C	Set 4	101	Milk	Dairy	Goat	Goat farm	MSR2025-00101	24.10.2025	Satu Mare County	47.93	23.45
S2-GOA-012C	Set 4	102	Milk	Dairy	Goat	Goat farm	MSR2025-00102	24.10.2025	Satu Mare County	47.93	23.45
S2-GOA-013C	Set 4	103	Milk	Dairy	Goat	Goat farm	MSR2025-00103	24.10.2025	Satu Mare County	47.93	23.45
S2-GOA-014C	Set 4	104	Milk	Dairy	Goat	Goat farm	MSR2025-00104	24.10.2025	Satu Mare County	47.93	23.45
S2-GOA-015C	Set 4	105	Milk	Dairy	Goat	Goat farm	MSR2025-00105	24.10.2025	Satu Mare County	47.93	23.45
S2-GOA-016C	Set 4	106	Milk	Dairy	Goat	Goat farm	MSR2025-00106	24.10.2025	Satu Mare County	47.93	23.45
S2-GOA-017C	Set 4	107	Milk	Dairy	Goat	Goat farm	MSR2025-00107	24.10.2025	Satu Mare County	47.93	23.45
S2-GOA-018C	Set 4	108	Milk	Dairy	Goat	Goat farm	MSR2025-00108	24.10.2025	Satu Mare County	47.93	23.45
S2-GOA-019C	Set 4	109	Milk	Dairy	Goat	Goat farm	MSR2025-00109	24.10.2025	Satu Mare County	47.93	23.45
S2-GOA-020C	Set 4	110	Milk	Dairy	Goat	Goat farm	MSR2025-00110	24.10.2025	Satu Mare County	47.93	23.45
S2-GOA-021C	Set 4	111	Milk	Dairy	Goat	Goat farm	MSR2025-00111	24.10.2025	Satu Mare County	47.93	23.45
S2-GOA-022C	Set 4	112	Milk	Dairy	Goat	Goat farm	MSR2025-00112	24.10.2025	Satu Mare County	47.93	23.45
S2-GOA-023C	Set 4	113	Milk	Dairy	Goat	Goat farm	MSR2025-00113	24.10.2025	Satu Mare County	47.93	23.45
S2-GOA-024C	Set 4	114	Milk	Dairy	Goat	Goat farm	MSR2025-00114	24.10.2025	Satu Mare County	47.93	23.45
S2-GOA-025C	Set 4	115	Milk	Dairy	Goat	Goat farm	MSR2025-00115	24.10.2025	Satu Mare County	47.93	23.45
S2-GOA-026C	Set 4	116	Milk	Dairy	Goat	Goat farm	MSR2025-00116	24.10.2025	Satu Mare County	47.93	23.45
S2-GOA-027C	Set 4	117	Milk	Dairy	Goat	Goat farm	MSR2025-00117	24.10.2025	Satu Mare County	47.93	23.45
S2-GOA-028C	Set 4	118	Milk	Dairy	Goat	Goat farm	MSR2025-00118	24.10.2025	Satu Mare County	47.93	23.45
S2-GOA-029C	Set 4	119	Milk	Dairy	Goat	Goat farm	MSR2025-00119	24.10.2025	Satu Mare County	47.93	23.45
S2-GOA-030C	Set 4	120	Milk	Dairy	Goat	Goat farm	MSR2025-00120	24.10.2025	Satu Mare County	47.93	23.45

All goat milk samples (S2-GOA-001C to S2-GOA-030C) were collected from a local goat farm in north-western Romania (Satu Mare County) on 24 October 2025. Freshly milked goat milk was filtered and transferred into stainless-steel collection tanks immediately after milking. Following standard dairy hygiene protocols, the milk was cooled to 2–4 °C within the farm's refrigerated storage system. Each unit was labeled according to the internal traceability system (MSR2025-00091 to MSR2025-00120) and stored under refrigerated conditions until transport for laboratory analysis.

Table S5. Identification and Metadata of Buffalo Milk Samples Collected from a Buffalo Farm in North-western Romania (Satu Mare County)

Identification Code	Set	Sample No.	Sample Type	Source	Animal	Farm	Sample ID	Collection Date	Locality	Latitude (°N)	Longitude (°E)
S2-BUF-001B	Set 5	121	Milk	Dairy	Buffalo	Buffalo farm	MSR2025-00121	24.10.2025	Satu Mare County	47.88	23.50
S2-BUF-002B	Set 5	122	Milk	Dairy	Buffalo	Buffalo farm	MSR2025-00122	24.10.2025	Satu Mare County	47.88	23.50
S2-BUF-003B	Set 5	123	Milk	Dairy	Buffalo	Buffalo farm	MSR2025-00123	24.10.2025	Satu Mare County	47.88	23.50

S2-BUF-004B	Set 5	124	Milk	Dairy	Buffalo	Buffalo farm	MSR2025-00124	24.10.2025	Satu Mare County	47.88	23.50
S2-BUF-005B	Set 5	125	Milk	Dairy	Buffalo	Buffalo farm	MSR2025-00125	24.10.2025	Satu Mare County	47.88	23.50
S2-BUF-006B	Set 5	126	Milk	Dairy	Buffalo	Buffalo farm	MSR2025-00126	24.10.2025	Satu Mare County	47.88	23.50
S2-BUF-007B	Set 5	127	Milk	Dairy	Buffalo	Buffalo farm	MSR2025-00127	24.10.2025	Satu Mare County	47.88	23.50
S2-BUF-008B	Set 5	128	Milk	Dairy	Buffalo	Buffalo farm	MSR2025-00128	24.10.2025	Satu Mare County	47.88	23.50
S2-BUF-009B	Set 5	129	Milk	Dairy	Buffalo	Buffalo farm	MSR2025-00129	24.10.2025	Satu Mare County	47.88	23.50
S2-BUF-010B	Set 5	130	Milk	Dairy	Buffalo	Buffalo farm	MSR2025-00130	24.10.2025	Satu Mare County	47.88	23.50
S2-BUF-011B	Set 5	131	Milk	Dairy	Buffalo	Buffalo farm	MSR2025-00131	24.10.2025	Satu Mare County	47.88	23.50
S2-BUF-012B	Set 5	132	Milk	Dairy	Buffalo	Buffalo farm	MSR2025-00132	24.10.2025	Satu Mare County	47.88	23.50
S2-BUF-013B	Set 5	133	Milk	Dairy	Buffalo	Buffalo farm	MSR2025-00133	24.10.2025	Satu Mare County	47.88	23.50
S2-BUF-014B	Set 5	134	Milk	Dairy	Buffalo	Buffalo farm	MSR2025-00134	24.10.2025	Satu Mare County	47.88	23.50
S2-BUF-015B	Set 5	135	Milk	Dairy	Buffalo	Buffalo farm	MSR2025-00135	24.10.2025	Satu Mare County	47.88	23.50
S2-BUF-016B	Set 5	136	Milk	Dairy	Buffalo	Buffalo farm	MSR2025-00136	24.10.2025	Satu Mare County	47.88	23.50
S2-BUF-017B	Set 5	137	Milk	Dairy	Buffalo	Buffalo farm	MSR2025-00137	24.10.2025	Satu Mare County	47.88	23.50
S2-BUF-018B	Set 5	138	Milk	Dairy	Buffalo	Buffalo farm	MSR2025-00138	24.10.2025	Satu Mare County	47.88	23.50
S2-BUF-019B	Set 5	139	Milk	Dairy	Buffalo	Buffalo farm	MSR2025-00139	24.10.2025	Satu Mare County	47.88	23.50
S2-BUF-020B	Set 5	140	Milk	Dairy	Buffalo	Buffalo farm	MSR2025-00140	24.10.2025	Satu Mare County	47.88	23.50
S2-BUF-021B	Set 5	141	Milk	Dairy	Buffalo	Buffalo farm	MSR2025-00141	24.10.2025	Satu Mare County	47.88	23.50
S2-BUF-022B	Set 5	142	Milk	Dairy	Buffalo	Buffalo farm	MSR2025-00142	24.10.2025	Satu Mare County	47.88	23.50
S2-BUF-023B	Set 5	143	Milk	Dairy	Buffalo	Buffalo farm	MSR2025-00143	24.10.2025	Satu Mare County	47.88	23.50
S2-BUF-024B	Set 5	144	Milk	Dairy	Buffalo	Buffalo farm	MSR2025-00144	24.10.2025	Satu Mare County	47.88	23.50
S2-BUF-025B	Set 5	145	Milk	Dairy	Buffalo	Buffalo farm	MSR2025-00145	24.10.2025	Satu Mare County	47.88	23.50
S2-BUF-026B	Set 5	146	Milk	Dairy	Buffalo	Buffalo farm	MSR2025-00146	24.10.2025	Satu Mare County	47.88	23.50
S2-BUF-027B	Set 5	147	Milk	Dairy	Buffalo	Buffalo farm	MSR2025-00147	24.10.2025	Satu Mare County	47.88	23.50
S2-BUF-028B	Set 5	148	Milk	Dairy	Buffalo	Buffalo farm	MSR2025-00148	24.10.2025	Satu Mare County	47.88	23.50
S2-BUF-029B	Set 5	149	Milk	Dairy	Buffalo	Buffalo farm	MSR2025-00149	24.10.2025	Satu Mare County	47.88	23.50
S2-BUF-030B	Set 5	150	Milk	Dairy	Buffalo	Buffalo farm	MSR2025-00150	24.10.2025	Satu Mare County	47.88	23.50
S2-BUF-031B	Set 5	151	Milk	Dairy	Buffalo	Buffalo farm	MSR2025-00151	24.10.2025	Satu Mare County	47.88	23.50
S2-BUF-032B	Set 5	152	Milk	Dairy	Buffalo	Buffalo farm	MSR2025-00152	24.10.2025	Satu Mare County	47.88	23.50

S2-BUF-033B	Set 5	153	Milk	Dairy	Buffalo	Buffalo farm	MSR2025-00153	24.10.2025	Satu Mare County	47.88	23.50
S2-BUF-034B	Set 5	154	Milk	Dairy	Buffalo	Buffalo farm	MSR2025-00154	24.10.2025	Satu Mare County	47.88	23.50
S2-BUF-035B	Set 5	155	Milk	Dairy	Buffalo	Buffalo farm	MSR2025-00155	24.10.2025	Satu Mare County	47.88	23.50
S2-BUF-036B	Set 5	156	Milk	Dairy	Buffalo	Buffalo farm	MSR2025-00156	24.10.2025	Satu Mare County	47.88	23.50
S2-BUF-037B	Set 5	157	Milk	Dairy	Buffalo	Buffalo farm	MSR2025-00157	24.10.2025	Satu Mare County	47.88	23.50
S2-BUF-038B	Set 5	158	Milk	Dairy	Buffalo	Buffalo farm	MSR2025-00158	24.10.2025	Satu Mare County	47.88	23.50
S2-BUF-039B	Set 5	159	Milk	Dairy	Buffalo	Buffalo farm	MSR2025-00159	24.10.2025	Satu Mare County	47.88	23.50
S2-BUF-040B	Set 5	160	Milk	Dairy	Buffalo	Buffalo farm	MSR2025-00160	24.10.2025	Satu Mare County	47.88	23.50

All buffalo milk samples (S2-BUF-001B to S2-BUF-040B) were collected from a buffalo farm in north-western Romania (Satu Mare County) on 24 October 2025. Fresh milk was filtered into stainless-steel (inox) tanks, cooled to 2–4 °C, and—after normal cooling—placed under refrigeration. Each unit was labeled using the internal traceability codes MSR2025-00121 to MSR2025-00160 and maintained under cold conditions until transport for laboratory analysis.

Table S6. Identification and Metadata of Donkey Milk Samples Collected from a Donkey Farm in North-western Romania (Satu Mare County)

Identification Code	Set	Sample No.	Sample Type	Source	Animal	Farm	Sample ID	Collection Date	Locality	Latitude (°N)	Longitude (°E)
S2-DNK-001M	Set 6	161	Milk	Dairy	Donkey	Donkey farm	MSR2025-00161	24.10.2025	Satu Mare County	47.88	23.50
S2-DNK-002M	Set 6	162	Milk	Dairy	Donkey	Donkey farm	MSR2025-00162	24.10.2025	Satu Mare County	47.88	23.50
S2-DNK-003M	Set 6	163	Milk	Dairy	Donkey	Donkey farm	MSR2025-00163	24.10.2025	Satu Mare County	47.88	23.50
S2-DNK-004M	Set 6	164	Milk	Dairy	Donkey	Donkey farm	MSR2025-00164	24.10.2025	Satu Mare County	47.88	23.50
S2-DNK-005M	Set 6	165	Milk	Dairy	Donkey	Donkey farm	MSR2025-00165	24.10.2025	Satu Mare County	47.88	23.50

S2-DNK-006M	Set 6	166	Milk	Dairy	Donkey	Donkey farm	MSR2025-00166	24.10.2025	Satu Mare County	47.88	23.50
S2-DNK-007M	Set 6	167	Milk	Dairy	Donkey	Donkey farm	MSR2025-00167	24.10.2025	Satu Mare County	47.88	23.50
S2-DNK-008M	Set 6	168	Milk	Dairy	Donkey	Donkey farm	MSR2025-00168	24.10.2025	Satu Mare County	47.88	23.50
S2-DNK-009M	Set 6	169	Milk	Dairy	Donkey	Donkey farm	MSR2025-00169	24.10.2025	Satu Mare County	47.88	23.50
S2-DNK-010M	Set 6	170	Milk	Dairy	Donkey	Donkey farm	MSR2025-00170	24.10.2025	Satu Mare County	47.88	23.50
S2-DNK-011M	Set 6	171	Milk	Dairy	Donkey	Donkey farm	MSR2025-00171	24.10.2025	Satu Mare County	47.88	23.50
S2-DNK-012M	Set 6	172	Milk	Dairy	Donkey	Donkey farm	MSR2025-00172	24.10.2025	Satu Mare County	47.88	23.50
S2-DNK-013M	Set 6	173	Milk	Dairy	Donkey	Donkey farm	MSR2025-00173	24.10.2025	Satu Mare County	47.88	23.50
S2-DNK-014M	Set 6	174	Milk	Dairy	Donkey	Donkey farm	MSR2025-00174	24.10.2025	Satu Mare County	47.88	23.50
S2-DNK-015M	Set 6	175	Milk	Dairy	Donkey	Donkey farm	MSR2025-00175	24.10.2025	Satu Mare County	47.88	23.50
S2-DNK-016M	Set 6	176	Milk	Dairy	Donkey	Donkey farm	MSR2025-00176	24.10.2025	Satu Mare County	47.88	23.50
S2-DNK-017M	Set 6	177	Milk	Dairy	Donkey	Donkey farm	MSR2025-00177	24.10.2025	Satu Mare County	47.88	23.50
S2-DNK-018M	Set 6	178	Milk	Dairy	Donkey	Donkey farm	MSR2025-00178	24.10.2025	Satu Mare County	47.88	23.50
S2-DNK-019M	Set 6	179	Milk	Dairy	Donkey	Donkey farm	MSR2025-00179	24.10.2025	Satu Mare County	47.88	23.50
S2-DNK-020M	Set 6	180	Milk	Dairy	Donkey	Donkey farm	MSR2025-00180	24.10.2025	Satu Mare County	47.88	23.50
S2-DNK-021M	Set 6	181	Milk	Dairy	Donkey	Donkey farm	MSR2025-00181	24.10.2025	Satu Mare County	47.88	23.50
S2-DNK-022M	Set 6	182	Milk	Dairy	Donkey	Donkey farm	MSR2025-00182	24.10.2025	Satu Mare County	47.88	23.50
S2-DNK-023M	Set 6	183	Milk	Dairy	Donkey	Donkey farm	MSR2025-00183	24.10.2025	Satu Mare County	47.88	23.50
S2-DNK-024M	Set 6	184	Milk	Dairy	Donkey	Donkey farm	MSR2025-00184	24.10.2025	Satu Mare County	47.88	23.50
S2-DNK-025M	Set 6	185	Milk	Dairy	Donkey	Donkey farm	MSR2025-00185	24.10.2025	Satu Mare County	47.88	23.50
S2-DNK-026M	Set 6	186	Milk	Dairy	Donkey	Donkey farm	MSR2025-00186	24.10.2025	Satu Mare County	47.88	23.50
S2-DNK-027M	Set 6	187	Milk	Dairy	Donkey	Donkey farm	MSR2025-00187	24.10.2025	Satu Mare County	47.88	23.50
S2-DNK-028M	Set 6	188	Milk	Dairy	Donkey	Donkey farm	MSR2025-00188	24.10.2025	Satu Mare County	47.88	23.50
S2-DNK-029M	Set 6	189	Milk	Dairy	Donkey	Donkey farm	MSR2025-00189	24.10.2025	Satu Mare County	47.88	23.50
S2-DNK-030M	Set 6	190	Milk	Dairy	Donkey	Donkey farm	MSR2025-00190	24.10.2025	Satu Mare County	47.88	23.50

All donkey milk samples (S2-DNK-001M to S2-DNK-030M) were collected from a donkey farm in north-western Romania (Satu Mare County) on 24 October 2025. Fresh milk was hand-milked following hygienic farm protocols and immediately transferred into stainless steel (inox) tanks. After collection, the milk was cooled to 2–4 °C and stored under refrigeration to preserve compositional integrity until transport for laboratory analysis. Each sample was labeled using internal traceability codes (MSR2025-00161 to MSR2025-00190), ensuring consistent tracking throughout storage, transport, and analysis.

Table S7. Identification and Metadata of Retail Conventional Cow Dairy Products and Buffalo Farm-Origin Dairy Products Analyzed in This Study

Identification Code	Set	Sample No.	Sample Type	Source	Animal	Farm/Origin	Sample ID	Collection Date	Locality	Latitude (°N)	Longitude (°E)	Notes
LM1	Set 7	1	Milk	Dairy	Cow	Retail store (commercial sample)	—	30.10.2025	—	—	—	Commercial sample purchased from retail; exact store address not recorded.
LM2	Set 7	2	Milk	Dairy	Cow	Retail store (commercial sample)	—	30.10.2025	—	—	—	Commercial sample purchased from retail; exact store address not recorded.
LM3	Set 7	3	Milk	Dairy	Cow	Retail store (commercial sample)	—	30.10.2025	—	—	—	Commercial sample purchased from retail; exact store address not recorded.

LM4	Set 7	4	Milk	Dairy	Cow	Retail store (commercial sample)	—	30.10.2025	—	—	—	Commercial sample purchased from retail; exact store address not recorded.
LM5	Set 7	5	Milk	Dairy	Cow	Retail store (commercial sample)	—	30.10.2025	—	—	—	Commercial sample purchased from retail; exact store address not recorded.
LM6	Set 7	6	Milk	Dairy	Cow	Retail store (commercial sample)	—	30.10.2025	—	—	—	Commercial sample purchased from retail; exact store address not recorded.
LM7	Set 7	7	Milk	Dairy	Cow	Retail store (commercial sample)	—	30.10.2025	—	—	—	Commercial sample purchased from retail; exact store address not recorded.
LM8	Set 7	8	Milk	Dairy	Cow	Retail store (commercial sample)	—	30.10.2025	—	—	—	Commercial sample purchased from retail; exact store address not recorded.
LM9	Set 7	9	Milk	Dairy	Cow	Retail store (commercial sample)	—	30.10.2025	—	—	—	Commercial sample purchased from retail; exact store address not recorded.
LM10	Set 7	10	Milk	Dairy	Cow	Retail store (commercial sample)	—	30.10.2025	—	—	—	Commercial sample purchased from retail; exact store address not recorded.
LM11	Set 7	11	Milk	Dairy	Cow	Retail store (commercial sample)	—	30.10.2025	—	—	—	Commercial sample purchased from retail; exact store address not recorded.
LM12	Set 7	12	Milk	Dairy	Cow	Retail store (commercial sample)	—	30.10.2025	—	—	—	Commercial sample purchased from retail; exact store address not recorded.
LM13	Set 7	13	Milk	Dairy	Cow	Retail store (commercial sample)	—	30.10.2025	—	—	—	Commercial sample purchased from retail; exact store address not recorded.
LM14	Set 7	14	Milk	Dairy	Cow	Retail store (commercial sample)	—	30.10.2025	—	—	—	Commercial sample purchased from retail; exact store address not recorded.
LM15	Set 7	15	Milk	Dairy	Cow	Retail store (commercial sample)	—	30.10.2025	—	—	—	Commercial sample purchased from retail; exact store address not recorded.
LM16	Set 7	16	Milk	Dairy	Cow	Retail store (commercial sample)	—	30.10.2025	—	—	—	Commercial sample purchased from retail; exact store address not recorded.
LM17	Set 7	17	Milk	Dairy	Cow	Retail store (commercial sample)	—	30.10.2025	—	—	—	Commercial sample purchased from retail; exact store address not recorded.

LM18	Set 7	18	Milk	Dairy	Cow	Retail store (commercial sample)	—	30.10.2025	—	—	—	Commercial sample purchased from retail; exact store address not recorded.
LM19	Set 7	19	Milk	Dairy	Cow	Retail store (commercial sample)	—	30.10.2025	—	—	—	Commercial sample purchased from retail; exact store address not recorded.
LM20	Set 7	20	Milk	Dairy	Cow	Retail store (commercial sample)	—	30.10.2025	—	—	—	Commercial sample purchased from retail; exact store address not recorded.
IM1	Set 8	1	Yogurt	Dairy	Cow	Retail store (commercial sample)	—	30.10.2025	—	—	—	Commercial sample purchased from retail; exact store address not recorded.
IM2	Set 8	2	Yogurt	Dairy	Cow	Retail store (commercial sample)	—	30.10.2025	—	—	—	Commercial sample purchased from retail; exact store address not recorded.
IM3	Set 8	3	Yogurt	Dairy	Cow	Retail store (commercial sample)	—	30.10.2025	—	—	—	Commercial sample purchased from retail; exact store address not recorded.
IM4	Set 8	4	Yogurt	Dairy	Cow	Retail store (commercial sample)	—	30.10.2025	—	—	—	Commercial sample purchased from retail; exact store address not recorded.
IM5	Set 8	5	Yogurt	Dairy	Cow	Retail store (commercial sample)	—	30.10.2025	—	—	—	Commercial sample purchased from retail; exact store address not recorded.
IM6	Set 8	6	Yogurt	Dairy	Cow	Retail store (commercial sample)	—	30.10.2025	—	—	—	Commercial sample purchased from retail; exact store address not recorded.
IM7	Set 8	7	Yogurt	Dairy	Cow	Retail store (commercial sample)	—	30.10.2025	—	—	—	Commercial sample purchased from retail; exact store address not recorded.
IM8	Set 8	8	Yogurt	Dairy	Cow	Retail store (commercial sample)	—	30.10.2025	—	—	—	Commercial sample purchased from retail; exact store address not recorded.
IM9	Set 8	9	Yogurt	Dairy	Cow	Retail store (commercial sample)	—	30.10.2025	—	—	—	Commercial sample purchased from retail; exact store address not recorded.
IM10	Set 8	10	Yogurt	Dairy	Cow	Retail store (commercial sample)	—	30.10.2025	—	—	—	Commercial sample purchased from retail; exact store address not recorded.
IM11	Set 8	11	Yogurt	Dairy	Cow	Retail store (commercial sample)	—	30.10.2025	—	—	—	Commercial sample purchased from retail; exact store address not recorded.

IM12	Set 8	12	Yogurt	Dairy	Cow	Retail store (commercial sample)	—	30.10.2025	—	—	—	Commercial sample purchased from retail; exact store address not recorded.
IM13	Set 8	13	Yogurt	Dairy	Cow	Retail store (commercial sample)	—	30.10.2025	—	—	—	Commercial sample purchased from retail; exact store address not recorded.
IM14	Set 8	14	Yogurt	Dairy	Cow	Retail store (commercial sample)	—	30.10.2025	—	—	—	Commercial sample purchased from retail; exact store address not recorded.
IM15	Set 8	15	Yogurt	Dairy	Cow	Retail store (commercial sample)	—	30.10.2025	—	—	—	Commercial sample purchased from retail; exact store address not recorded.
IM16	Set 8	16	Yogurt	Dairy	Cow	Retail store (commercial sample)	—	30.10.2025	—	—	—	Commercial sample purchased from retail; exact store address not recorded.
IM17	Set 8	17	Yogurt	Dairy	Cow	Retail store (commercial sample)	—	30.10.2025	—	—	—	Commercial sample purchased from retail; exact store address not recorded.
IM18	Set 8	18	Yogurt	Dairy	Cow	Retail store (commercial sample)	—	30.10.2025	—	—	—	Commercial sample purchased from retail; exact store address not recorded.
IM19	Set 8	19	Yogurt	Dairy	Cow	Retail store (commercial sample)	—	30.10.2025	—	—	—	Commercial sample purchased from retail; exact store address not recorded.
IM20	Set 8	20	Yogurt	Dairy	Cow	Retail store (commercial sample)	—	30.10.2025	—	—	—	Commercial sample purchased from retail; exact store address not recorded.
BM1	Set 9	1	Cheese	Dairy	Cow	Retail store (commercial sample)	—	30.10.2025	—	—	—	Commercial sample purchased from retail; exact store address not recorded.
BM2	Set 9	2	Cheese	Dairy	Cow	Retail store (commercial sample)	—	30.10.2025	—	—	—	Commercial sample purchased from retail; exact store address not recorded.
BM3	Set 9	3	Cheese	Dairy	Cow	Retail store (commercial sample)	—	30.10.2025	—	—	—	Commercial sample purchased from retail; exact store address not recorded.
BM4	Set 9	4	Cheese	Dairy	Cow	Retail store (commercial sample)	—	30.10.2025	—	—	—	Commercial sample purchased from retail; exact store address not recorded.
BM5	Set 9	5	Cheese	Dairy	Cow	Retail store (commercial sample)	—	30.10.2025	—	—	—	Commercial sample purchased from retail; exact store address not recorded.

BM6	Set 9	6	Cheese	Dairy	Cow	Retail store (commercial sample)	—	30.10.2025	—	—	—	Commercial sample purchased from retail; exact store address not recorded.
BM7	Set 9	7	Cheese	Dairy	Cow	Retail store (commercial sample)	—	30.10.2025	—	—	—	Commercial sample purchased from retail; exact store address not recorded.
BM8	Set 9	8	Cheese	Dairy	Cow	Retail store (commercial sample)	—	30.10.2025	—	—	—	Commercial sample purchased from retail; exact store address not recorded.
BM9	Set 9	9	Cheese	Dairy	Cow	Retail store (commercial sample)	—	30.10.2025	—	—	—	Commercial sample purchased from retail; exact store address not recorded.
BM10	Set 9	10	Cheese	Dairy	Cow	Retail store (commercial sample)	—	30.10.2025	—	—	—	Commercial sample purchased from retail; exact store address not recorded.
BM11	Set 9	11	Cheese	Dairy	Cow	Retail store (commercial sample)	—	30.10.2025	—	—	—	Commercial sample purchased from retail; exact store address not recorded.
BM12	Set 9	12	Cheese	Dairy	Cow	Retail store (commercial sample)	—	30.10.2025	—	—	—	Commercial sample purchased from retail; exact store address not recorded.
BM13	Set 9	13	Cheese	Dairy	Cow	Retail store (commercial sample)	—	30.10.2025	—	—	—	Commercial sample purchased from retail; exact store address not recorded.
BM14	Set 9	14	Cheese	Dairy	Cow	Retail store (commercial sample)	—	30.10.2025	—	—	—	Commercial sample purchased from retail; exact store address not recorded.
BM15	Set 9	15	Cheese	Dairy	Cow	Retail store (commercial sample)	—	30.10.2025	—	—	—	Commercial sample purchased from retail; exact store address not recorded.
BM16	Set 9	16	Cheese	Dairy	Cow	Retail store (commercial sample)	—	30.10.2025	—	—	—	Commercial sample purchased from retail; exact store address not recorded.
BM17	Set 9	17	Cheese	Dairy	Cow	Retail store (commercial sample)	—	30.10.2025	—	—	—	Commercial sample purchased from retail; exact store address not recorded.
BM18	Set 9	18	Cheese	Dairy	Cow	Retail store (commercial sample)	—	30.10.2025	—	—	—	Commercial sample purchased from retail; exact store address not recorded.
BM19	Set 9	19	Cheese	Dairy	Cow	Retail store (commercial sample)	—	30.10.2025	—	—	—	Commercial sample purchased from retail; exact store address not recorded.

BM20	Set 9	20	Cheese	Dairy	Cow	Retail store (commercial sample)	—	30.10.2025	—	—	—	Commercial sample purchased from retail; exact store address not recorded.
BB1	Set 10	1	Mozzarella	Dairy	Buffalo	Buffalo farm	—	30.10.2025	Certeze, Satu Mare	47.88940	23.50280	Coordinates harmonized with Buffalo farm site used elsewhere in dataset.
BB2	Set 10	2	Mozzarella	Dairy	Buffalo	Buffalo farm	—	30.10.2025	Certeze, Satu Mare	47.88940	23.50280	Coordinates harmonized with Buffalo farm site used elsewhere in dataset.
BB3	Set 10	3	Mozzarella	Dairy	Buffalo	Buffalo farm	—	30.10.2025	Certeze, Satu Mare	47.88940	23.50280	Coordinates harmonized with Buffalo farm site used elsewhere in dataset.
BB4	Set 10	4	Mozzarella	Dairy	Buffalo	Buffalo farm	—	30.10.2025	Certeze, Satu Mare	47.88940	23.50280	Coordinates harmonized with Buffalo farm site used elsewhere in dataset.
BB5	Set 10	5	Mozzarella	Dairy	Buffalo	Buffalo farm	—	30.10.2025	Certeze, Satu Mare	47.88940	23.50280	Coordinates harmonized with Buffalo farm site used elsewhere in dataset.
BB6	Set 10	6	Mozzarella	Dairy	Buffalo	Buffalo farm	—	30.10.2025	Certeze, Satu Mare	47.88940	23.50280	Coordinates harmonized with Buffalo farm site used elsewhere in dataset.
BB7	Set 10	7	Mozzarella	Dairy	Buffalo	Buffalo farm	—	30.10.2025	Certeze, Satu Mare	47.88940	23.50280	Coordinates harmonized with Buffalo farm site used elsewhere in dataset.
BB8	Set 10	8	Mozzarella	Dairy	Buffalo	Buffalo farm	—	30.10.2025	Certeze, Satu Mare	47.88940	23.50280	Coordinates harmonized with Buffalo farm site used elsewhere in dataset.
BB9	Set 10	9	Mozzarella	Dairy	Buffalo	Buffalo farm	—	30.10.2025	Certeze, Satu Mare	47.88940	23.50280	Coordinates harmonized with Buffalo farm site used elsewhere in dataset.
BB10	Set 10	10	Mozzarella	Dairy	Buffalo	Buffalo farm	—	30.10.2025	Certeze, Satu Mare	47.88940	23.50280	Coordinates harmonized with Buffalo farm site used elsewhere in dataset.
BB11	Set 10	11	Mozzarella	Dairy	Buffalo	Buffalo farm	—	30.10.2025	Certeze, Satu Mare	47.88940	23.50280	Coordinates harmonized with Buffalo farm site used elsewhere in dataset.
BB12	Set 10	12	Mozzarella	Dairy	Buffalo	Buffalo farm	—	30.10.2025	Certeze, Satu Mare	47.88940	23.50280	Coordinates harmonized with Buffalo farm site used elsewhere in dataset.
BB13	Set 10	13	Mozzarella	Dairy	Buffalo	Buffalo farm	—	30.10.2025	Certeze, Satu Mare	47.88940	23.50280	Coordinates harmonized with Buffalo farm site used elsewhere in dataset.

BB14	Set 10	14	Mozzarella	Dairy	Buffalo	Buffalo farm	—	30.10.2025	Certeze, Satu Mare	47.88940	23.50280	Coordinates harmonized with Buffalo farm site used elsewhere in dataset.
BB15	Set 10	15	Mozzarella	Dairy	Buffalo	Buffalo farm	—	30.10.2025	Certeze, Satu Mare	47.88940	23.50280	Coordinates harmonized with Buffalo farm site used elsewhere in dataset.
BB16	Set 10	16	Mozzarella	Dairy	Buffalo	Buffalo farm	—	30.10.2025	Certeze, Satu Mare	47.88940	23.50280	Coordinates harmonized with Buffalo farm site used elsewhere in dataset.
BB17	Set 10	17	Mozzarella	Dairy	Buffalo	Buffalo farm	—	30.10.2025	Certeze, Satu Mare	47.88940	23.50280	Coordinates harmonized with Buffalo farm site used elsewhere in dataset.
BB18	Set 10	18	Mozzarella	Dairy	Buffalo	Buffalo farm	—	30.10.2025	Certeze, Satu Mare	47.88940	23.50280	Coordinates harmonized with Buffalo farm site used elsewhere in dataset.
BB19	Set 10	19	Mozzarella	Dairy	Buffalo	Buffalo farm	—	30.10.2025	Certeze, Satu Mare	47.88940	23.50280	Coordinates harmonized with Buffalo farm site used elsewhere in dataset.
BB20	Set 10	20	Mozzarella	Dairy	Buffalo	Buffalo farm	—	30.10.2025	Certeze, Satu Mare	47.88940	23.50280	Coordinates harmonized with Buffalo farm site used elsewhere in dataset.
BB21	Set 10	21	Mozzarella	Dairy	Buffalo	Buffalo farm	—	30.10.2025	Certeze, Satu Mare	47.88940	23.50280	Coordinates harmonized with Buffalo farm site used elsewhere in dataset.
BB22	Set 10	22	Mozzarella	Dairy	Buffalo	Buffalo farm	—	30.10.2025	Certeze, Satu Mare	47.88940	23.50280	Coordinates harmonized with Buffalo farm site used elsewhere in dataset.
BB23	Set 10	23	Mozzarella	Dairy	Buffalo	Buffalo farm	—	30.10.2025	Certeze, Satu Mare	47.88940	23.50280	Coordinates harmonized with Buffalo farm site used elsewhere in dataset.
BB24	Set 10	24	Mozzarella	Dairy	Buffalo	Buffalo farm	—	30.10.2025	Certeze, Satu Mare	47.88940	23.50280	Coordinates harmonized with Buffalo farm site used elsewhere in dataset.
BB25	Set 10	25	Mozzarella	Dairy	Buffalo	Buffalo farm	—	30.10.2025	Certeze, Satu Mare	47.88940	23.50280	Coordinates harmonized with Buffalo farm site used elsewhere in dataset.
BB26	Set 10	26	Mozzarella	Dairy	Buffalo	Buffalo farm	—	30.10.2025	Certeze, Satu Mare	47.88940	23.50280	Coordinates harmonized with Buffalo farm site used elsewhere in dataset.
BB27	Set 10	27	Mozzarella	Dairy	Buffalo	Buffalo farm	—	30.10.2025	Certeze, Satu Mare	47.88940	23.50280	Coordinates harmonized with Buffalo farm site used elsewhere in dataset.

BB28	Set 10	28	Mozzarella	Dairy	Buffalo	Buffalo farm	—	30.10.2025	Certeze, Satu Mare	47.88940	23.50280	Coordinates harmonized with Buffalo farm site used elsewhere in dataset.
BB29	Set 10	29	Mozzarella	Dairy	Buffalo	Buffalo farm	—	30.10.2025	Certeze, Satu Mare	47.88940	23.50280	Coordinates harmonized with Buffalo farm site used elsewhere in dataset.
BB30	Set 10	30	Mozzarella	Dairy	Buffalo	Buffalo farm	—	30.10.2025	Certeze, Satu Mare	47.88940	23.50280	Coordinates harmonized with Buffalo farm site used elsewhere in dataset.
IB1	Set 11	1	Yogurt	Dairy	Buffalo	Buffalo farm	—	30.10.2025	Certeze, Satu Mare	47.88940	23.50280	Coordinates harmonized with Buffalo farm site used elsewhere in dataset.
IB2	Set 11	2	Yogurt	Dairy	Buffalo	Buffalo farm	—	30.10.2025	Certeze, Satu Mare	47.88940	23.50280	Coordinates harmonized with Buffalo farm site used elsewhere in dataset.
IB3	Set 11	3	Yogurt	Dairy	Buffalo	Buffalo farm	—	30.10.2025	Certeze, Satu Mare	47.88940	23.50280	Coordinates harmonized with Buffalo farm site used elsewhere in dataset.
IB4	Set 11	4	Yogurt	Dairy	Buffalo	Buffalo farm	—	30.10.2025	Certeze, Satu Mare	47.88940	23.50280	Coordinates harmonized with Buffalo farm site used elsewhere in dataset.
IB5	Set 11	5	Yogurt	Dairy	Buffalo	Buffalo farm	—	30.10.2025	Certeze, Satu Mare	47.88940	23.50280	Coordinates harmonized with Buffalo farm site used elsewhere in dataset.
IB6	Set 11	6	Yogurt	Dairy	Buffalo	Buffalo farm	—	30.10.2025	Certeze, Satu Mare	47.88940	23.50280	Coordinates harmonized with Buffalo farm site used elsewhere in dataset.
IB7	Set 11	7	Yogurt	Dairy	Buffalo	Buffalo farm	—	30.10.2025	Certeze, Satu Mare	47.88940	23.50280	Coordinates harmonized with Buffalo farm site used elsewhere in dataset.
IB8	Set 11	8	Yogurt	Dairy	Buffalo	Buffalo farm	—	30.10.2025	Certeze, Satu Mare	47.88940	23.50280	Coordinates harmonized with Buffalo farm site used elsewhere in dataset.
IB9	Set 11	9	Yogurt	Dairy	Buffalo	Buffalo farm	—	30.10.2025	Certeze, Satu Mare	47.88940	23.50280	Coordinates harmonized with Buffalo farm site used elsewhere in dataset.
IB10	Set 11	10	Yogurt	Dairy	Buffalo	Buffalo farm	—	30.10.2025	Certeze, Satu Mare	47.88940	23.50280	Coordinates harmonized with Buffalo farm site used elsewhere in dataset.
IB11	Set 11	11	Yogurt	Dairy	Buffalo	Buffalo farm	—	30.10.2025	Certeze, Satu Mare	47.88940	23.50280	Coordinates harmonized with Buffalo farm site used elsewhere in dataset.

IB12	Set 11	12	Yogurt	Dairy	Buffalo	Buffalo farm	—	30.10.2025	Certeze, Satu Mare	47.88940	23.50280	Coordinates harmonized with Buffalo farm site used elsewhere in dataset.
IB13	Set 11	13	Yogurt	Dairy	Buffalo	Buffalo farm	—	30.10.2025	Certeze, Satu Mare	47.88940	23.50280	Coordinates harmonized with Buffalo farm site used elsewhere in dataset.
IB14	Set 11	14	Yogurt	Dairy	Buffalo	Buffalo farm	—	30.10.2025	Certeze, Satu Mare	47.88940	23.50280	Coordinates harmonized with Buffalo farm site used elsewhere in dataset.
IB15	Set 11	15	Yogurt	Dairy	Buffalo	Buffalo farm	—	30.10.2025	Certeze, Satu Mare	47.88940	23.50280	Coordinates harmonized with Buffalo farm site used elsewhere in dataset.
IB16	Set 11	16	Yogurt	Dairy	Buffalo	Buffalo farm	—	30.10.2025	Certeze, Satu Mare	47.88940	23.50280	Coordinates harmonized with Buffalo farm site used elsewhere in dataset.
IB17	Set 11	17	Yogurt	Dairy	Buffalo	Buffalo farm	—	30.10.2025	Certeze, Satu Mare	47.88940	23.50280	Coordinates harmonized with Buffalo farm site used elsewhere in dataset.
IB18	Set 11	18	Yogurt	Dairy	Buffalo	Buffalo farm	—	30.10.2025	Certeze, Satu Mare	47.88940	23.50280	Coordinates harmonized with Buffalo farm site used elsewhere in dataset.
IB19	Set 11	19	Yogurt	Dairy	Buffalo	Buffalo farm	—	30.10.2025	Certeze, Satu Mare	47.88940	23.50280	Coordinates harmonized with Buffalo farm site used elsewhere in dataset.
IB20	Set 11	20	Yogurt	Dairy	Buffalo	Buffalo farm	—	30.10.2025	Certeze, Satu Mare	47.88940	23.50280	Coordinates harmonized with Buffalo farm site used elsewhere in dataset.
IB21	Set 11	21	Yogurt	Dairy	Buffalo	Buffalo farm	—	30.10.2025	Certeze, Satu Mare	47.88940	23.50280	Coordinates harmonized with Buffalo farm site used elsewhere in dataset.
IB22	Set 11	22	Yogurt	Dairy	Buffalo	Buffalo farm	—	30.10.2025	Certeze, Satu Mare	47.88940	23.50280	Coordinates harmonized with Buffalo farm site used elsewhere in dataset.
IB23	Set 11	23	Yogurt	Dairy	Buffalo	Buffalo farm	—	30.10.2025	Certeze, Satu Mare	47.88940	23.50280	Coordinates harmonized with Buffalo farm site used elsewhere in dataset.
IB24	Set 11	24	Yogurt	Dairy	Buffalo	Buffalo farm	—	30.10.2025	Certeze, Satu Mare	47.88940	23.50280	Coordinates harmonized with Buffalo farm site used elsewhere in dataset.
IB25	Set 11	25	Yogurt	Dairy	Buffalo	Buffalo farm	—	30.10.2025	Certeze, Satu Mare	47.88940	23.50280	Coordinates harmonized with Buffalo farm site used elsewhere in dataset.

IB26	Set 11	26	Yogurt	Dairy	Buffalo	Buffalo farm	—	30.10.2025	Certeze, Satu Mare	47.88940	23.50280	Coordinates harmonized with Buffalo farm site used elsewhere in dataset.
IB27	Set 11	27	Yogurt	Dairy	Buffalo	Buffalo farm	—	30.10.2025	Certeze, Satu Mare	47.88940	23.50280	Coordinates harmonized with Buffalo farm site used elsewhere in dataset.

LM = Conventional cow milk retail products (n = 20) purchased in sealed packaging from retail outlets in north-western Romania on 30 October 2025. IM = Conventional cow yogurt retail products (n = 20) purchased from the same region and date; products were industrially fermented and stored at 2–4 °C prior to analysis. BM = Conventional cow cheese retail products (n = 20) purchased from retail outlets on 30 October 2025; products were derived from pasteurized milk of unspecified farm origin. BB = Buffalo mozzarella samples (n = 30) obtained from a buffalo dairy farm in north-western Romania (Satu Mare County) on 30 October 2025; products were vacuum-packed and stored at 2–4 °C until analysis. IB = Buffalo yogurt samples (n = 27) produced at the same buffalo dairy farm (Satu Mare County) under equivalent hygienic and technological conditions and stored at 2–4 °C until analysis. All dairy matrices were transported in refrigerated containers (2–4 °C) to the analytical laboratory and analyzed within 24 h of collection/purchase. This composite dataset (Sets 7–11) integrates both retail conventional and farm-origin products, enabling comparative assessment across species (cow vs. buffalo) and processing stages (milk, yogurt, cheese). Each sample was labeled, logged, and assigned an internal identification code (LM1–LM20, IM1–IM20, BM1–BM20, BB1–BB30, IB1–IB27) to ensure traceability throughout the analytical workflow.

2. Supplementary Material (Text S1)

2.2.1. Standardized Technological Procedure for the Production of Organic Cow's Milk

Organic cow's milk was obtained under controlled hygienic conditions during routine milking. Immediately after milking, the raw milk was rapidly cooled to 4 °C in the bulk cooling tank and homogenized for 10 minutes using a mechanical stirring system to ensure uniform composition. The milk was kept at 4 ± 2 °C without any heat treatment, additives, or preservatives until sampling for laboratory analysis.

2.2.2. Standardized Technological Procedure for the Production of Cow's Milk

Conventional cow's milk was obtained under standard industrial processing conditions. After milking, the milk was cooled to 6–8 °C in the bulk tank and mechanically stirred for 5–7 minutes to ensure uniform fat and solids distribution. Before packaging and distribution, the milk underwent pasteurization at 72–75 °C for 15–20 seconds, followed by rapid cooling to 4 °C to ensure microbial safety and extended shelf life. The milk was maintained at 4 ± 2 °C until laboratory sampling and analysis.

2.2.3. Standardized Technological Procedure for the Production of Buffalo Milk

Buffalo milk was produced under controlled hygienic conditions during routine milking. After collection, the milk was cooled to 6–8 °C in the bulk tank and gently mixed for 8–10 minutes to maintain uniform composition, given its higher fat and total solids content compared to cow's milk. Prior to laboratory sampling, the milk underwent heat treatment at 65–70 °C for 15–20 minutes (low-temperature long-time pasteurization), followed by rapid cooling to 4 °C to preserve physicochemical quality. The milk was stored refrigerated at 4 ± 2 °C until analytical processing.

2.2.4. Standardized Technological Procedure for the Production of Goat Milk

Goat milk was obtained under controlled hygienic conditions during routine milking. Immediately after collection, the milk was cooled to 4–6 °C in the bulk storage tank and homogenized for 6–8 minutes to ensure uniform distribution of fat and solids. Due to its naturally higher biological activity and lower heat stability compared with cow and buffalo milk, the product received mild thermal treatment at 65 °C for 10–15 minutes to reduce microbial load without altering nutritional quality. After heating, the milk was rapidly cooled to 4 °C and stored under refrigerated conditions at 4 ± 2 °C until sampling and laboratory analysis.

2.2.5. Standardized Technological Procedure for the Production of Commercial Cow's Milk

Commercial cow milk used in this study was purchased from authorized retail outlets under original sealed packaging. The milk had already undergone industrial processing in accordance with national food quality and safety standards, including pasteurization or UHT treatment, depending on product labeling, followed by homogenization and commercial packaging. The products were maintained under the manufacturer-specified refrigeration conditions (2–6 °C) throughout purchase, transport, and laboratory handling, without any additional processing prior to analytical evaluation.

2.2.6. Standardized Technological Procedure for the Production of Organic Cow's Cheese

Organic cow's cheese was produced from certified organic cow's milk obtained under controlled hygienic conditions. Raw organic milk was rapidly cooled to 4 °C after milking and stored under refrigeration until processing. Before cheese manufacture, the milk was pasteurized at 75 °C for 20 s and then cooled to 34 °C. The milk was inoculated with organic mesophilic starter cultures (*Lactococcus* spp., *Leuconostoc* spp.) and gently mixed to ensure uniform distribution. Organic rennet was then added at a rate of 20 mL

per 100 L of milk, and coagulation was carried out at 34 °C until a firm curd was obtained. The curd was cut into cubes, allowed to drain, molded, and ripened at 8–14 °C and 85–90 % relative humidity for 2–3 days before sampling and analysis.

2.2.7. Standardized Technological Procedure for the Production of Commercial Cow's Cheese

Commercial cow's cheese used in this study was obtained as a finished retail product purchased from specialized dairy shops under sealed original packaging. The cheese was industrially manufactured according to standard technological procedures established by the producer, typically involving pasteurization of cow's milk, coagulation with microbial or animal rennet, curd cutting, molding, salting, and controlled ripening under industrial hygiene conditions. Final products were packaged in vacuum-sealed or airtight commercial packaging and stored under refrigeration at 2–6 °C prior to purchase.

For laboratory analysis, retail cheese units were transported in insulated refrigerated containers (maintaining 2–4 °C) and logged upon arrival without undergoing any additional processing steps. All samples remained in original packaging until the moment of analytical preparation to ensure product authenticity and traceability.

2.2.8. Standardized Technological Procedure for the Production of Buffalo Mozzarella Cheese

Mozzarella cheese from buffalo milk was produced using a traditional artisanal procedure. Fresh raw buffalo milk was used without prior boiling. Immediately after milking, the milk was gently mixed with freshly squeezed lemon juice to initiate acidification. A thermophilic starter culture (*Streptococcus thermophilus*) was added, and the mixture was allowed to rest for approximately 10 minutes to promote initial fermentation.

Commercial rennet was then incorporated according to the manufacturer's recommended dose, and the mixture was maintained at 35 °C for 30 minutes to allow proper coagulation. Once a firm curd was formed, the curd mass was cut and the whey was separated. The curd was subsequently left to mature for approximately 4 hours to achieve the required elastic structure characteristic of pasta-filata cheeses.

After maturation, the cheese was stretched and salted by immersion in brine solution, then shaped into balls and cooled prior to sampling and analytical evaluation.

2.2.9. Standardized Technological Procedure for the Production of Organic Yogurt

Organic yogurt was produced from freshly collected organic cow's milk. The milk was pasteurized at 85 °C for 15 minutes, then cooled to 42 ± 1 °C. A defined starter culture containing *Lactobacillus delbrueckii subsp. bulgaricus* and *Streptococcus thermophilus* was added at 2% (v/v) and gently mixed until homogenized. The inoculated milk was poured into sterile fermentation containers and incubated at 42 °C for 4–6 hours, until the target pH reached 4.5 ± 0.1. After fermentation, cooling was performed rapidly to 4 °C to stop bacterial activity. The final product was stored under refrigeration at 2–4 °C until sampling and laboratory analysis.

2.2.10. Standardized Technological Procedure for the Production of Commercial Yogurt

Commercial cow's milk yogurt was industrially produced from pasteurized standardized milk. Milk fat and protein were adjusted to commercial specifications (2.5–3.5% fat; 3–3.3% protein) prior to heat treatment at 90–95 °C for 5–10 minutes, followed by cooling to 43 ± 1 °C. Commercial thermophilic yogurt cultures (*S. thermophilus* and *L. bulgaricus*) were added at 1.5–2% (v/v) and mixed to achieve uniform inoculation. Fermentation occurred in sealed retail containers at 42–43 °C for 3–5 hours, until pH reached 4.4–4.6. After fermentation, units were transferred to rapid cooling chambers and stored at 2–4 °C until distribution and sampling.

2.2.11. Standardized Technological Procedure for the Production of Buffalo Yogurt

Buffalo milk yogurt was produced from fresh buffalo milk collected at the dairy farm. The milk was pasteurized at 90 °C for 10 minutes, then cooled to 41 ± 1 °C. A thermophilic starter consisting of *Lactobacillus delbrueckii* subsp. *bulgaricus* and *Streptococcus thermophilus* was added at 2–2.5% (v/v) to account for higher solids content. Fermentation was performed in sterile containers at 41–42 °C for 4–6 hours, until target pH 4.5 was reached. The yogurt was then rapidly cooled to 4 °C and stored under refrigeration at 2–4 °C until sampling.

Table S8. Microwave digestion conditions for dairy matrices using Milestone START D

Matrix type	Examples included	Sample amount	Reagents added (per vessel)	Microwave temperature program (Milestone START D, 4 steps, Tmax = 200 °C)	Notes
Liquid dairy matrices	Organic and commercial cow, goat, buffalo and donkey milk; organic and commercial yogurt	0.50 ± 0.01 mL	5 mL HNO_3 (65%, TraceMetal™ Grade, Merck Supelco) + 2 mL H_2O_2 (30%, Trace Analysis Grade, Sigma-Aldrich)	Ramp 1: RT → 100 °C in 10 min, hold 5 min; Ramp 2: 100 → 150 °C in 10 min, hold 10 min; Ramp 3: 150 → 200 °C in 10 min, hold 20 min; Ramp 4 (cooling): passive cooling 200 → ≤60 °C in ~20–30 min, 0 W	Gentle pre-reaction before sealing vessels to reduce pressure build-up
Solid dairy matrices	Organic and commercial cow cheese; buffalo mozzarella	0.50 ± 0.01 g	5 mL HNO_3 (65%, TraceMetal™ Grade, Merck Supelco) + 2 mL H_2O_2 (30%, Trace Analysis Grade, Sigma-Aldrich)	Same 4-step program: RT → 100 °C (10 min, 5 min hold); 100 → 150 °C (10 min, 10 min hold); 150 → 200 °C (10 min, 20 min hold); cooling 200 → ≤60 °C (20–30 min, 0 W)	High-fat matrices; ensure thorough homogenization before weighing

Table S9. The instrumental settings (a) and data acquisition parameters (b) of the ICP-MS system, which define the operating conditions and analytical procedures for precise metal quantification

**(a) Instrumental parameters**		**(b) Data acquisition parameters for quantitative mode**	
RF power/W	1.4 kW	Measuring mode	Standard (Ar 5.0)<br>Q Cell (Collision Cell) (He 6.0)
Argon (Ar) gas flow, Helium (He) gas flow		Point per peak	3
Nebulizer	1.0 L/min.	Scans/Replicate	7
Plasma gas low rate (Ar 5.0)	18.0 L/min.	Replicate/Sample	7
Auxiliary gas flow rate (He 6.0)	0.20 L/min.		

Lens voltage	37 V	Dwell time (ms)	3
Mirror lens right	32 V		
Mirror lens bottom	31 V		
Sample uptake rate	90 s	Integration time	1-5 ms
Temperature spray chamber		2.10 °C	
Background correction		2 points/peak	
Injector tube		quartz 2 mm id	
Sample cone		Sample Cone 4450	
Skimmer cone		Ni – Skimmer iCAP Q 0.5 mm insert version	
Nebulizer		MicroMist Nebulizer 0.4 mL/min.	

Table S10. Calibration Levels and Instrumental Parameters for ICP–MS Determination of Trace Metals in Dairy Matrices

Element	Calibration Concentration Levels (µg/L)[1]	Regression Model	Minimum r^2	m/z[3]	Collision Cell Mode[4]	Internal Standard[2]
Pb	0, 1, 5, 10, 25, 50, 100	Linear	≥ 0.995	208	He / KED	Bi
Cd	0, 0.2, 0.5, 1, 5, 10, 50	Linear	≥ 0.995	111	He / KED	In
Hg	0, 0.5, 1, 5, 10, 25, 50	Linear	≥ 0.995	202	He / KED	Au stabilizer / In
As	0, 1, 5, 10, 25, 50, 100	Linear	≥ 0.995	75	He / KED	Ge
Cr	0, 1, 5, 10, 25, 50, 100	Linear	≥ 0.995	52	He / KED	Sc
Ni	0, 2, 5, 10, 25, 50, 200	Linear	≥ 0.995	60	He / KED	In
Al	0, 10, 25, 50, 100, 250, 500	Linear	≥ 0.995	27	He / KED	Sc
Sn	0, 2, 5, 10, 25, 50, 200	Linear	≥ 0.995	118	He / KED	Rh
Cu	0, 10, 25, 50, 100, 250, 500	Linear	≥ 0.995	63	He / KED	Rh
Zn	0, 10, 25, 50, 100, 250, 500	Linear	≥ 0.995	66	He / KED	Ge

[1] Calibration curves were constructed using seven concentration levels prepared from certified multi-element standard solutions (CRM) diluted in 2% HNO_3 (suprapure grade). Linearity was evaluated through linear regression, with acceptance criteria of $r^2 \geq 0.995$. [2] Internal standards (Bi, In, Ge, Sc, Rh) were continuously introduced online to compensate for matrix effects, signal drift, and variations in nebulization efficiency. [3] m/z values represent the monitored isotopes selected for optimal sensitivity and reduced background noise. [4] He / KED refers to helium collision mode with kinetic energy discrimination, used to minimize polyatomic and spectral interferences associated with dairy matrices. Quality control verification was conducted at the beginning, middle, and end of each run using a mid-range calibration standard, with acceptable deviation thresholds set at ±10%. Procedural blanks and rinse solutions were analyzed intermittently to monitor possible contamination and carry-over between samples. Calibration levels were selected to cover expected concentration ranges for trace metals in dairy products based on regulatory guidelines and previously reported literature values.

Table S11. CRM Performance and Quality Control Evaluation for ICP–MS Quantification of Trace Metals in Dairy Matrices

Element	Certified Value (mg/kg)	Uncertainty	Measured Value (mg/kg)	Recovery (%)	Acceptance Range (80–120%)	Result
Pb	0.019	±0.003	0.0185	97.4%	80–120%	Accepted
Cd	0.0005	±0.0002	0.00048	96.0%	80–120%	Accepted
Hg	0.0003	±0.0002	0.00029	96.7%	80–120%	Accepted
As (info value)	0.0019	–	0.00185	97.4%	Indicative	Acceptable
Cr	0.0026	±0.0007	0.00270	103.8%	80–120%	Accepted
Ni (non-certified)	0.068	±0.014	0.066	97.1%	For trend only	Acceptable
Al (info value)	2.0	–	1.92	96.0%	Indicative	Acceptable
Sn (info value)	<0.02	–	0.018	–	Qualitative only	Acceptable
Cu	0.70	±0.10	0.69	98.6%	80–120%	Accepted

Zn	46.1	±2.2	45.5	98.7%	80–120%	Accepted

Certified values and associated uncertainties for NIST SRM 1549 Non-Fat Milk Powder are provided by the National Institute of Standards and Technology (NIST) and represent mass fractions expressed in mg/kg on a dry-weight basis. Values marked as "info value" or "non-certified" are included only for methodological reference and do not carry full certification status; therefore, evaluation is based on indicative comparison rather than strict recovery assessment. Recovery (%) was calculated as the ratio between the measured concentration and the certified value multiplied by 100, and analytical performance was considered acceptable when recoveries fell within the 80–120% range, following international validation standards (ISO/IEC 17025 and AOAC guidelines). Results marked as "Qualitative only" correspond to elements present below quantification limits or certification thresholds and were evaluated solely through presence/absence confirmation. Measured values represent the mean of three analytical replicates (n = 3) and demonstrate high accuracy and reproducibility, with all analytes falling within the acceptance range for trace metal quantification in dairy matrices.

Table S12. Verification of Analytical Accuracy Using FAPAS Whole Milk Quality Control Material and Z-Score Interpretation

Element	Assigned Value (mg/kg)	SD	Measured Value (mg/kg)	Z-score	Acceptance Limit	Result
Pb	0.028	0.006	0.0284	+0.07	$\lvert Z \rvert \leq 2$	Accepted
Cd	0.003	0.001	0.00310	+0.10	$\lvert Z \rvert \leq 2$	Accepted
Hg	0.0012	0.0005	0.00115	–0.10	$\lvert Z \rvert \leq 2$	Accepted
As	0.002	0.0007	0.00188	–0.17	$\lvert Z \rvert \leq 2$	Accepted
Cr	0.005	0.002	0.0049	–0.05	$\lvert Z \rvert \leq 2$	Accepted
Ni	0.012	0.004	0.0123	+0.07	$\lvert Z \rvert \leq 2$	Accepted
Al	0.42	0.10	0.415	–0.05	$\lvert Z \rvert \leq 2$	Accepted
Sn	0.010	0.004	0.0102	+0.05	$\lvert Z \rvert \leq 2$	Accepted
Cu	0.92	0.13	0.94	+0.15	$\lvert Z \rvert \leq 2$	Accepted
Zn	37.2	3.8	36.9	–0.08	$\lvert Z \rvert \leq 2$	Accepted

The FAPAS Whole Milk quality control material was used to independently verify the accuracy of the analytical procedure. Assigned values represent consensus means established through interlaboratory proficiency testing. Z-scores were calculated using the formula: (Measured value – Assigned value) / Standard deviation, and analytical performance was considered satisfactory when $\lvert Z \rvert \leq 2$, in accordance with international proficiency testing criteria (ISO 17043 and IUPAC recommendations). All elements tested in the QC material demonstrated Z-scores well within acceptable limits, confirming excellent analytical accuracy and the suitability of the ICP–MS method for trace metal determination in dairy matrices. Measured values represent the mean of triplicate determinations (n = 3).

Table S13. Analytical dataset of heavy metal concentrations (mg/kg) in organic cow milk samples (n = 30)

Identification Code	Sample ID	Pb	Cd	Hg	As	Cr	Ni	Al	Sn	Cu	Zn
S1-MLK-001	MSR2025-00001	0.00949 ± 0.00057	0.00092 ± 0.00007	BLD	0.00094 ± 0.00007	0.0139 ± 0.0007	0.0112 ± 0.0006	0.0187 ± 0.0011	0.0037 ± 0.0003	0.141 ± 0.008	4.98 ± 0.20
S1-MLK-002	MSR2025-00002	0.00822 ± 0.00049	0.00110 ± 0.00009	BLD	0.00113 ± 0.00009	0.0104 ± 0.0005	0.0107 ± 0.0005	0.0230 ± 0.0014	0.0037 ± 0.0003	0.146 ± 0.009	5.11 ± 0.20
S1-MLK-003	MSR2025-00003	0.00980 ± 0.00059	0.00078 ± 0.00006	BLD	0.00087 ± 0.00007	0.0125 ± 0.0006	0.0101 ± 0.0005	0.0198 ± 0.0012	0.0037 ± 0.0003	0.121 ± 0.007	4.43 ± 0.18
S1-MLK-004	MSR2025-00004	0.01155 ± 0.00069	0.00135 ± 0.00011	BLD	0.00122 ± 0.00010	0.0117 ± 0.0006	0.0139 ± 0.0007	0.0225 ± 0.0014	0.0039 ± 0.0003	0.146 ± 0.009	4.78 ± 0.19
S1-MLK-005	MSR2025-00005	0.00803 ± 0.00048	0.00073 ± 0.00006	BLD	0.00090 ± 0.00007	0.0109 ± 0.0005	0.0105 ± 0.0005	0.0217 ± 0.0013	0.0037 ± 0.0003	0.137 ± 0.008	4.39 ± 0.18
S1-MLK-006	MSR2025-00006	0.00803 ± 0.00048	0.00116 ± 0.00009	BLD	0.00108 ± 0.00009	0.0128 ± 0.0006	0.0114 ± 0.0006	0.0187 ± 0.0011	0.0031 ± 0.0002	0.134 ± 0.008	4.70 ± 0.19
S1-MLK-007	MSR2025-00007	0.01166 ± 0.00070	0.00051 ± 0.00004	BLD	0.00102 ± 0.00008	0.0126 ± 0.0006	0.0120 ± 0.0006	0.0269 ± 0.0016	0.0033 ± 0.0002	0.128 ± 0.008	4.30 ± 0.17
S1-MLK-008	MSR2025-00008	0.01003 ± 0.00060	0.00070 ± 0.00006	BLD	0.00091 ± 0.00007	0.0120 ± 0.0006	0.0084 ± 0.0004	0.0214 ± 0.0013	0.0036 ± 0.0003	0.143 ± 0.009	4.23 ± 0.17
S1-MLK-009	MSR2025-00009	0.00756 ± 0.00045	0.00116 ± 0.00009	BLD	0.00127 ± 0.00010	0.0094 ± 0.0005	0.0118 ± 0.0006	0.0224 ± 0.0013	0.0038 ± 0.0003	0.117 ± 0.007	4.62 ± 0.18
S1-MLK-010	MSR2025-00010	0.00959 ± 0.00058	0.00132 ± 0.00011	BLD	0.00122 ± 0.00010	0.0138 ± 0.0007	0.0096 ± 0.0005	0.0203 ± 0.0012	0.0032 ± 0.0002	0.129 ± 0.008	4.85 ± 0.19
S1-MLK-011	MSR2025-00011	0.00757 ± 0.00045	0.00115 ± 0.00009	BLD	0.00116 ± 0.00009	0.0115 ± 0.0006	0.0106 ± 0.0005	0.0228 ± 0.0014	0.0039 ± 0.0003	0.129 ± 0.008	5.38 ± 0.22
S1-MLK-012	MSR2025-00012	0.00757 ± 0.00045	0.00107 ± 0.00009	BLD	0.00106 ± 0.00008	0.0128 ± 0.0006	0.0103 ± 0.0005	0.0247 ± 0.0015	0.0036 ± 0.0003	0.139 ± 0.008	4.23 ± 0.17
S1-MLK-013	MSR2025-00013	0.00898 ± 0.00054	0.00101 ± 0.00008	BLD	0.00114 ± 0.00009	0.0123 ± 0.0006	0.0103 ± 0.0005	0.0225 ± 0.0014	0.0032 ± 0.0002	0.149 ± 0.009	5.27 ± 0.21
S1-MLK-014	MSR2025-00014	0.00467 ± 0.00028	0.00066 ± 0.00005	BLD	0.00092 ± 0.00007	0.0088 ± 0.0004	0.0094 ± 0.0005	0.0243 ± 0.0015	0.0036 ± 0.0003	0.120 ± 0.007	4.80 ± 0.19
S1-MLK-015	MSR2025-00015	0.00505 ± 0.00030	0.00088 ± 0.00007	BLD	0.00114 ± 0.00009	0.0110 ± 0.0006	0.0103 ± 0.0005	0.0202 ± 0.0012	0.0029 ± 0.0002	0.126 ± 0.008	4.41 ± 0.18
S1-MLK-016	MSR2025-00016	0.00647 ± 0.00039	0.00142 ± 0.00011	BLD	0.00095 ± 0.00008	0.0123 ± 0.0006	0.0112 ± 0.0006	0.0241 ± 0.0014	0.0030 ± 0.0002	0.135 ± 0.008	4.84 ± 0.19
S1-MLK-017	MSR2025-00017	0.00647 ± 0.00039	0.00142 ± 0.00011	BLD	0.00088 ± 0.00007	0.0131 ± 0.0007	0.0123 ± 0.0006	0.0199 ± 0.0012	0.0032 ± 0.0002	0.140 ± 0.008	4.88 ± 0.20
S1-MLK-018	MSR2025-00018	0.00913 ± 0.00055	0.00120 ± 0.00010	BLD	0.00113 ± 0.00009	0.0102 ± 0.0005	0.0108 ± 0.0005	0.0192 ± 0.0012	0.0034 ± 0.0002	0.132 ± 0.008	4.56 ± 0.18
S1-MLK-019	MSR2025-00019	0.00668 ± 0.00040	0.00057 ± 0.00005	BLD	0.00141 ± 0.00011	0.0141 ± 0.0007	0.0115 ± 0.0006	0.0172 ± 0.0010	0.0039 ± 0.0003	0.134 ± 0.008	4.83 ± 0.19
S1-MLK-020	MSR2025-00020	0.00568 ± 0.00034	0.00120 ± 0.00010	BLD	0.00097 ± 0.00008	0.0130 ± 0.0007	0.0117 ± 0.0006	0.0192 ± 0.0012	0.0037 ± 0.0003	0.146 ± 0.009	4.65 ± 0.19
S1-MLK-021	MSR2025-00021	0.01143 ± 0.00069	0.00098 ± 0.00008	BLD	0.00147 ± 0.00012	0.0135 ± 0.0007	0.0130 ± 0.0007	0.0226 ± 0.0014	0.0035 ± 0.0002	0.173 ± 0.010	4.85 ± 0.19
S1-MLK-022	MSR2025-00022	0.00805 ± 0.00048	0.00090 ± 0.00007	BLD	0.00088 ± 0.00007	0.0117 ± 0.0006	0.0130 ± 0.0007	0.0256 ± 0.0015	0.0035 ± 0.0002	0.161 ± 0.010	5.06 ± 0.20
S1-MLK-023	MSR2025-00023	0.00864 ± 0.00052	0.00128 ± 0.00010	BLD	0.00128 ± 0.00010	0.0104 ± 0.0005	0.0082 ± 0.0004	0.0245 ± 0.0015	0.0038 ± 0.0003	0.147 ± 0.009	4.30 ± 0.17
S1-MLK-024	MSR2025-00024	0.00565 ± 0.00034	0.00141 ± 0.00011	BLD	0.00063 ± 0.00005	0.0104 ± 0.0005	0.0103 ± 0.0005	0.0191 ± 0.0011	0.0029 ± 0.0002	0.131 ± 0.008	4.74 ± 0.19
S1-MLK-025	MSR2025-00025	0.00741 ± 0.00044	0.00138 ± 0.00011	BLD	0.00107 ± 0.00009	0.0097 ± 0.0005	0.0101 ± 0.0005	0.0250 ± 0.0015	0.0037 ± 0.0003	0.135 ± 0.008	5.04 ± 0.20
S1-MLK-026	MSR2025-00026	0.00872 ± 0.00052	0.00085 ± 0.00007	BLD	0.00123 ± 0.00010	0.0131 ± 0.0007	0.0124 ± 0.0006	0.0239 ± 0.0014	0.0038 ± 0.0003	0.158 ± 0.009	4.91 ± 0.20
S1-MLK-027	MSR2025-00027	0.00620 ± 0.00037	0.00101 ± 0.00008	BLD	0.00113 ± 0.00009	0.0111 ± 0.0006	0.0104 ± 0.0005	0.0198 ± 0.0012	0.0029 ± 0.0002	0.140 ± 0.008	4.55 ± 0.18
S1-MLK-028	MSR2025-00028	0.00925 ± 0.00056	0.00120 ± 0.00010	BLD	0.00074 ± 0.00006	0.0172 ± 0.0009	0.0125 ± 0.0006	0.0255 ± 0.0015	0.0034 ± 0.0002	0.137 ± 0.008	4.72 ± 0.19
S1-MLK-029	MSR2025-00029	0.00730 ± 0.00044	0.00139 ± 0.00011	BLD	0.00147 ± 0.00012	0.0121 ± 0.0006	0.0139 ± 0.0007	0.0296 ± 0.0018	0.0027 ± 0.0002	0.175 ± 0.010	4.60 ± 0.18
S1-MLK-030	MSR2025-00030	0.00792 ± 0.00047	0.00096 ± 0.00008	BLD	0.00049 ± 0.00004	0.0115 ± 0.0006	0.0145 ± 0.0007	0.0170 ± 0.0010	0.0043 ± 0.0003	0.126 ± 0.008	4.56 ± 0.18

Table S14. Comprehensive statistical evaluation (descriptive, dispersion, distribution and censoring indices) of heavy metal concentrations (mg/kg) in organic cow milk samples (n = 30)

Comprehensive Analytical Dataset	Identification Code	Sample ID	Animal	Farm	Pb	Cd	Hg	As	Cr	Ni	Al	Sn	Cu	Zn
Mean	S1-MLK-001 S1-MLK-030	MSR2025-00001 MSR2025-00030	Cow	BIO Farm	0.00842	0.00107	BLD	0.00105	0.01188	0.01105	0.02204	0.00347	0.13823	4.71
SD					0.00182	0.00027	-	0.00021	0.00127	0.00141	0.00275	0.00038	0.01520	0.296
RSD %					22.50	25.15	-	19.71	10.69	12.97	12.48	10.94	11.00	6.27
SE					0.00033	0.000048	-	0.000038	0.00023	0.00026	0.00050	0.00007	0.00255	0.0540
CI95% Low					0.00741	0.00096	-	0.00096	0.01114	0.01050	0.02134	0.00333	0.13395	4.608
CI95% High					0.00877	0.00115	-	0.00115	0.01263	0.01160	0.02274	0.00361	0.14438	4.830
Skewness					0.1715	–0.3396	-	–0.2253	0.1892	0.6739	–0.0562	–0.1891	0.9124	0.1892
Kurtosis					–0.443	–0.374	-	–0.468	–0.343	0.056	–0.217	–0.468	0.397	–0.468
Shapiro–Wilk p-value					0.720	0.803	-	0.590	0.764	0.056	0.674	0.764	0.076	0.764
CV %					22.50%	25.15%	-	19.71%	10.69%	12.97%	12.48%	10.94%	11.00%	6.27%
median					0.00832	0.00108	-	0.00107	0.01190	0.01060	0.02225	0.00360	0.13700	4.72
min					0.00467	0.00051	-	0.00049	0.00880	0.00820	0.01700	0.00270	0.11700	4.23
max					0.01166	0.00142	-	0.00147	0.01720	0.01450	0.02960	0.00430	0.17500	5.38
LOQ					0.0003	0.00015	0.00006	0.00012	0.00025	0.00030	0.00060	0.00075	0.00150	0.015
LCD (%)					0	0	100	0	0	0	0	0	0	0

BLD = below limit of detection. LOQ = limit of quantification. LCD = left-censored data percentage (fraction of values < LOQ). Statistical indices include descriptive (mean, median, min, max) and inferential metrics (SD, SE, RSD%, CV%, CI95%, Skewness, Kurtosis, Shapiro–Wilk). Hg showed complete censoring (LCD = 100%), indicating concentrations consistently below instrumental quantification capacity. All remaining metals exhibited LCD = 0%, demonstrating robust quantification across samples. Metal concentrations expressed in mg/kg (wet basis). n = 30 samples. Analytical method: ICP-MS trace-element determination in dairy matrix.

Table S15. Analytical dataset of heavy metal concentrations (mg/kg) in conventional goat milk samples (n = 30)

Identification Code	Sample ID	Pb	Cd	Hg	As	Cr	Ni	Al	Sn	Cu	Zn
S2-GOA-001C	MSR2025-00091	0.00482 ± 0.00041	0.00074 ± 0.00011	BLD	0.00062 ± 0.00007	0.0105 ± 0.0011	0.0102 ± 0.0006	0.0214 ± 0.0014	0.0038 ± 0.0003	0.121 ± 0.010	4.52 ± 0.22

S2-GOA-002C	MSR2025-00092	0.00691 ± 0.00056	0.00110 ± 0.00014	BLD	0.00044 ± 0.00008	0.0129 ± 0.0013	0.0128 ± 0.0008	0.0305 ± 0.0022	0.0042 ± 0.0004	0.162 ± 0.013	5.31 ± 0.27
S2-GOA-003C	MSR2025-00093	0.00395 ± 0.00033	0.00052 ± 0.00008	BLD	0.00038 ± 0.00006	0.0092 ± 0.0009	0.0068 ± 0.0006	0.0148 ± 0.0011	0.0021 ± 0.0002	0.091 ± 0.007	3.82 ± 0.19
S2-GOA-004C	MSR2025-00094	0.00812 ± 0.00079	0.00118 ± 0.00015	BLD	0.00099 ± 0.00010	0.0156 ± 0.0014	0.0170 ± 0.0009	0.0294 ± 0.0021	0.0047 ± 0.0005	0.178 ± 0.015	5.44 ± 0.25
S2-GOA-005C	MSR2025-00095	0.00544 ± 0.00043	0.00084 ± 0.00012	BLD	0.00073 ± 0.00007	0.0118 ± 0.0010	0.0096 ± 0.0008	0.0235 ± 0.0016	0.0029 ± 0.0003	0.137 ± 0.012	4.35 ± 0.18
S2-GOA-006C	MSR2025-00096	0.00418 ± 0.00029	0.00047 ± 0.00007	BLD	0.00034 ± 0.00006	0.0084 ± 0.0008	0.0074 ± 0.0006	0.0137 ± 0.0010	0.0018 ± 0.0002	0.089 ± 0.006	3.76 ± 0.17
S2-GOA-007C	MSR2025-00097	0.00910 ± 0.00086	0.00096 ± 0.00011	BLD	0.00118 ± 0.00014	0.0165 ± 0.0017	0.0149 ± 0.0009	0.0318 ± 0.0025	0.0049 ± 0.0005	0.169 ± 0.014	5.23 ± 0.26
S2-GOA-008C	MSR2025-00098	0.00615 ± 0.00052	0.00074 ± 0.00010	BLD	0.00082 ± 0.00010	0.0134 ± 0.0013	0.0124 ± 0.0008	0.0269 ± 0.0020	0.0035 ± 0.0004	0.142 ± 0.011	4.96 ± 0.24
S2-GOA-009C	MSR2025-00099	0.00466 ± 0.00037	0.00061 ± 0.00007	BLD	0.00057 ± 0.00007	0.0090 ± 0.0009	0.0079 ± 0.0005	0.0178 ± 0.0013	0.0022 ± 0.0002	0.100 ± 0.008	4.10 ± 0.20
S2-GOA-010C	MSR2025-00100	0.00730 ± 0.00065	0.00089 ± 0.00009	BLD	0.00098 ± 0.00009	0.0144 ± 0.0010	0.0130 ± 0.0007	0.0227 ± 0.0017	0.0028 ± 0.0003	0.149 ± 0.012	4.68 ± 0.21
S2-GOA-011C	MSR2025-00101	0.00587 ± 0.00049	0.00082 ± 0.00009	BLD	0.00085 ± 0.00008	0.0129 ± 0.0012	0.0114 ± 0.0006	0.0206 ± 0.0015	0.0032 ± 0.0003	0.131 ± 0.011	4.74 ± 0.22
S2-GOA-012C	MSR2025-00102	0.00372 ± 0.00028	0.00044 ± 0.00006	BLD	0.00036 ± 0.00005	0.0079 ± 0.0007	0.0061 ± 0.0004	0.0120 ± 0.0009	0.0016 ± 0.0002	0.083 ± 0.006	3.63 ± 0.18
S2-GOA-013C	MSR2025-00103	0.00841 ± 0.00072	0.00112 ± 0.00014	BLD	0.00090 ± 0.00009	0.0151 ± 0.0014	0.0162 ± 0.0008	0.0291 ± 0.0019	0.0042 ± 0.0004	0.165 ± 0.013	5.18 ± 0.27
S2-GOA-014C	MSR2025-00104	0.00649 ± 0.00054	0.00092 ± 0.00010	BLD	0.00078 ± 0.00007	0.0111 ± 0.0011	0.0104 ± 0.0006	0.0198 ± 0.0014	0.0029 ± 0.0002	0.126 ± 0.009	4.33 ± 0.19
S2-GOA-015C	MSR2025-00105	0.00511 ± 0.00041	0.00073 ± 0.00008	BLD	0.00069 ± 0.00007	0.0101 ± 0.0009	0.0087 ± 0.0005	0.0163 ± 0.0012	0.0025 ± 0.0002	0.116 ± 0.010	4.07 ± 0.17
S2-GOA-016C	MSR2025-00106	0.00792 ± 0.00069	0.00104 ± 0.00012	BLD	0.00102 ± 0.00010	0.0168 ± 0.0015	0.0154 ± 0.0008	0.0252 ± 0.0018	0.0036 ± 0.0003	0.152 ± 0.012	4.52 ± 0.22
S2-GOA-017C	MSR2025-00107	0.00673 ± 0.00061	0.00085 ± 0.00009	BLD	0.00092 ± 0.00009	0.0132 ± 0.0012	0.0117 ± 0.0006	0.0184 ± 0.0013	0.0027 ± 0.0002	0.134 ± 0.011	4.83 ± 0.23
S2-GOA-018C	MSR2025-00108	0.00428 ± 0.00030	0.00053 ± 0.00007	BLD	0.00048 ± 0.00006	0.0082 ± 0.0006	0.0069 ± 0.0004	0.0150 ± 0.0010	0.0020 ± 0.0002	0.094 ± 0.007	3.89 ± 0.18
S2-GOA-019C	MSR2025-00109	0.00943 ± 0.00088	0.00118 ± 0.00012	BLD	0.00117 ± 0.00012	0.0174 ± 0.0016	0.0178 ± 0.0009	0.0320 ± 0.0023	0.0047 ± 0.0004	0.173 ± 0.014	5.39 ± 0.28
S2-GOA-020C	MSR2025-00110	0.00566 ± 0.00047	0.00076 ± 0.00009	BLD	0.00069 ± 0.00007	0.0116 ± 0.0010	0.0104 ± 0.0006	0.0180 ± 0.0014	0.0026 ± 0.0002	0.130 ± 0.010	4.12 ± 0.19
S2-GOA-021C	MSR2025-00111	0.00389 ± 0.00032	0.00061 ± 0.00008	BLD	0.00041 ± 0.00006	0.0093 ± 0.0008	0.0075 ± 0.0005	0.0167 ± 0.0012	0.0023 ± 0.0002	0.104 ± 0.008	3.98 ± 0.18
S2-GOA-022C	MSR2025-00112	0.00621 ± 0.00055	0.00097 ± 0.00011	BLD	0.00087 ± 0.00009	0.0127 ± 0.0011	0.0119 ± 0.0007	0.0230 ± 0.0016	0.0031 ± 0.0003	0.144 ± 0.012	4.67 ± 0.21
S2-GOA-023C	MSR2025-00113	0.00453 ± 0.00037	0.00069 ± 0.00008	BLD	0.00059 ± 0.00007	0.0109 ± 0.0009	0.0083 ± 0.0005	0.0201 ± 0.0013	0.0025 ± 0.0002	0.119 ± 0.009	4.22 ± 0.20
S2-GOA-024C	MSR2025-00114	0.00803 ± 0.00071	0.00088 ± 0.00010	BLD	0.00105 ± 0.00011	0.0149 ± 0.0013	0.0140 ± 0.0008	0.0276 ± 0.0020	0.0040 ± 0.0003	0.158 ± 0.013	5.07 ± 0.24
S2-GOA-025C	MSR2025-00115	0.00522 ± 0.00042	0.00081 ± 0.00009	BLD	0.00066 ± 0.00007	0.0113 ± 0.0010	0.0091 ± 0.0006	0.0190 ± 0.0014	0.0027 ± 0.0002	0.124 ± 0.010	4.30 ± 0.19
S2-GOA-026C	MSR2025-00116	0.00407 ± 0.00031	0.00055 ± 0.00007	BLD	0.00049 ± 0.00006	0.0086 ± 0.0008	0.0072 ± 0.0005	0.0154 ± 0.0011	0.0021 ± 0.0002	0.096 ± 0.007	3.86 ± 0.18
S2-GOA-027C	MSR2025-00117	0.00751 ± 0.00063	0.00102 ± 0.00012	BLD	0.00093 ± 0.00010	0.0139 ± 0.0012	0.0135 ± 0.0008	0.0247 ± 0.0017	0.0033 ± 0.0003	0.151 ± 0.012	4.91 ± 0.23
S2-GOA-028C	MSR2025-00118	0.00602 ± 0.00050	0.00079 ± 0.00009	BLD	0.00071 ± 0.00008	0.0124 ± 0.0011	0.0108 ± 0.0006	0.0210 ± 0.0015	0.0029 ± 0.0002	0.136 ± 0.011	4.41 ± 0.20
S2-GOA-029C	MSR2025-00119	0.00381 ± 0.00029	0.00046 ± 0.00006	BLD	0.00037 ± 0.00005	0.0076 ± 0.0007	0.0063 ± 0.0004	0.0129 ± 0.0010	0.0017 ± 0.0002	0.087 ± 0.006	3.71 ± 0.17
S2-GOA-030C	MSR2025-00120	0.00859 ± 0.00078	0.00108 ± 0.00013	BLD	0.00109 ± 0.00011	0.0161 ± 0.0015	0.0158 ± 0.0009	0.0288 ± 0.0021	0.0043 ± 0.0004	0.167 ± 0.014	5.26 ± 0.26

Table S16. Comprehensive statistical evaluation (descriptive, dispersion, distribution and censoring indices) of heavy metal concentrations (mg/kg) in goat milk samples (n = 30)

Comprehensive Analytical Dataset	Identification Code	Sample ID	Animal	Farm	Pb	Cd	Hg	As	Cr	Ni	Al	Sn	Cu	Zn
Mean	S2-GOA-001C S2-GOA-030C	MSR2025-00091 MSR2025-00120	Goat	CONVENTIONAL Farm	0.00607	0.000810	-	0.000736	0.0121	0.0110	0.0216	0.00306	0.131	4.51
SD					0.00174	0.000221	-	0.000256	0.0029	0.0034	0.0059	0.00095	0.028	0.56
RSD %					28.62	27.29	-	34.82	23.86	31.09	27.39	30.94	21.75	12.32
SE					0.00032	0.000040	-	0.000047	0.0005	0.0006	0.0011	0.00017	0.005	0.10
CI95% Low					0.00542	0.000727	-	0.000640	0.0110	0.0098	0.0194	0.00271	0.120	4.30
CI95% High					0.00672	0.000893	-	0.000832	0.0132	0.0123	0.0238	0.00341	0.142	4.72
Skewness					0.3344	-0.0324	-	0.0206	0.1371	0.3260	0.2313	0.3998	-0.1336	0.1403
Kurtosis					-1.077	-0.962	-	-1.126	-1.050	-0.972	-1.047	-0.830	-1.101	-1.205
Shapiro–Wilk p-value					0.099	0.357	-	0.225	0.320	0.202	0.268	0.214	0.216	0.173
CV %					28.62	27.29	-	34.82	23.86	31.09	27.39	30.94	21.75	12.32
median					0.00595	0.000815	-	0.000720	0.0121	0.0106	0.0208	0.00290	0.133	4.46
min					0.00372	0.000440	-	0.000340	0.0076	0.0061	0.0120	0.00160	0.083	3.63
max					0.00943	0.001180	-	0.001180	0.0174	0.0178	0.0320	0.00490	0.178	5.44
LOQ					0.0003	0.00015	0.00006	0.00012	0.00025	0.00030	0.00060	0.00075	0.00150	0.015
LCD (%)					0	0	100	0	0	0	0	0	0	0

BLD = below limit of detection. LOQ = limit of quantification. LCD = left-censored data percentage (fraction of values < LOQ). Statistical indices include descriptive (mean, median, min, max) and inferential metrics (SD, SE, RSD%, CV%, CI95%, Skewness, Kurtosis, Shapiro–Wilk). Hg showed complete censoring (LCD = 100%), indicating concentrations consistently below instrumental quantification capacity. All remaining metals exhibited LCD = 0%, demonstrating robust quantification across samples. Metal concentrations expressed in mg/kg (wet basis). n = 30 samples. Analytical method: ICP-MS trace-element determination in dairy matrix.

Table S17. Analytical dataset of heavy metal concentrations (mg/kg) in conventional buffalo milk samples (n = 40)

Identification Code	Sample ID	Pb	Cd	Hg	As	Cr	Ni	Al	Sn	Cu	Zn
S2-BUF-001B	MSR2025-00121	0.00310 ± 0.00012	0.00210 ± 0.00012	BLD	BLD	0.215 ± 0.015	0.28 ± 0.03	0.18 ± 0.02	0.00210 ± 0.00022	0.145 ± 0.012	4.85 ± 0.22

S2-BUF-002B	MSR2025-00122	0.00425 ± 0.00018	0.00345 ± 0.00018	BLD	0.00231 ± 0.00014	0.374 ± 0.022	0.41 ± 0.04	0.25 ± 0.03	0.00345 ± 0.00031	0.210 ± 0.015	6.12 ± 0.28
S2-BUF-003B	MSR2025-00123	0.00385 ± 0.00014	0.00482 ± 0.00022	BLD	BLD	0.298 ± 0.018	0.17 ± 0.02	0.09 ± 0.01	0.00185 ± 0.00018	0.118 ± 0.010	3.98 ± 0.19
S2-BUF-004B	MSR2025-00124	0.00510 ± 0.00016	0.00175 ± 0.00010	BLD	0.00142 ± 0.00009	0.452 ± 0.025	0.63 ± 0.05	0.44 ± 0.03	0.00410 ± 0.00035	0.305 ± 0.020	7.45 ± 0.32
S2-BUF-005B	MSR2025-00125	0.00448 ± 0.00011	0.00298 ± 0.00015	BLD	0.00328 ± 0.00017	0.187 ± 0.013	0.36 ± 0.03	0.21 ± 0.02	0.00272 ± 0.00024	0.265 ± 0.018	9.22 ± 0.38
S2-BUF-006B	MSR2025-00126	0.00332 ± 0.00009	0.00120 ± 0.00008	BLD	BLD	0.531 ± 0.028	0.55 ± 0.05	0.37 ± 0.03	0.00388 ± 0.00033	0.190 ± 0.014	5.34 ± 0.25
S2-BUF-007B	MSR2025-00127	0.00625 ± 0.00019	0.00565 ± 0.00028	BLD	0.00205 ± 0.00012	0.409 ± 0.021	0.19 ± 0.02	0.07 ± 0.01	0.00195 ± 0.00019	0.335 ± 0.022	11.10 ± 0.46
S2-BUF-008B	MSR2025-00128	0.00572 ± 0.00015	0.00425 ± 0.00021	BLD	0.00376 ± 0.00019	0.263 ± 0.017	0.47 ± 0.04	0.29 ± 0.02	0.00455 ± 0.00037	0.128 ± 0.011	4.22 ± 0.20
S2-BUF-009B	MSR2025-00129	0.00405 ± 0.00013	0.00305 ± 0.00016	BLD	0.00118 ± 0.00008	0.322 ± 0.019	0.22 ± 0.02	0.12 ± 0.01	0.00230 ± 0.00021	0.375 ± 0.025	13.48 ± 0.54
S2-BUF-010B	MSR2025-00130	0.00710 ± 0.00020	0.00780 ± 0.00034	BLD	0.00264 ± 0.00015	0.557 ± 0.029	0.69 ± 0.06	0.48 ± 0.03	0.00510 ± 0.00040	0.255 ± 0.018	7.90 ± 0.34
S2-BUF-011B	MSR2025-00131	0.00665 ± 0.00017	0.00642 ± 0.00029	BLD	BLD	0.341 ± 0.020	0.33 ± 0.03	0.19 ± 0.02	0.00305 ± 0.00027	0.182 ± 0.013	5.82 ± 0.26
S2-BUF-012B	MSR2025-00132	0.00355 ± 0.00012	0.00258 ± 0.00013	BLD	0.00195 ± 0.00011	0.479 ± 0.024	0.51 ± 0.05	0.33 ± 0.03	0.00218 ± 0.00020	0.410 ± 0.028	10.95 ± 0.45
S2-BUF-013B	MSR2025-00133	0.00475 ± 0.00018	0.00470 ± 0.00023	BLD	0.00312 ± 0.00018	0.196 ± 0.014	0.14 ± 0.02	0.11 ± 0.01	0.00402 ± 0.00034	0.225 ± 0.016	6.48 ± 0.29
S2-BUF-014B	MSR2025-00134	0.00515 ± 0.00014	0.00348 ± 0.00017	BLD	0.00107 ± 0.00008	0.388 ± 0.021	0.27 ± 0.03	0.22 ± 0.02	0.00176 ± 0.00017	0.148 ± 0.012	3.35 ± 0.16
S2-BUF-015B	MSR2025-00135	0.00690 ± 0.00020	0.00925 ± 0.00039	BLD	BLD	0.427 ± 0.022	0.58 ± 0.05	0.42 ± 0.03	0.00360 ± 0.00030	0.295 ± 0.020	8.12 ± 0.35
S2-BUF-016B	MSR2025-00136	0.00592 ± 0.00018	0.00815 ± 0.00033	BLD	0.00248 ± 0.00014	0.305 ± 0.018	0.38 ± 0.03	0.27 ± 0.02	0.00255 ± 0.00023	0.185 ± 0.013	5.15 ± 0.23
S2-BUF-017B	MSR2025-00137	0.00370 ± 0.00011	0.00105 ± 0.00007	BLD	0.00389 ± 0.00020	0.517 ± 0.027	0.11 ± 0.01	0.06 ± 0.01	0.00485 ± 0.00039	0.470 ± 0.030	14.30 ± 0.56
S2-BUF-018B	MSR2025-00138	0.00455 ± 0.00015	0.00272 ± 0.00014	BLD	0.00133 ± 0.00009	0.244 ± 0.016	0.43 ± 0.04	0.31 ± 0.03	0.00200 ± 0.00019	0.242 ± 0.017	6.95 ± 0.30
S2-BUF-019B	MSR2025-00139	0.00810 ± 0.00021	0.01080 ± 0.00041	BLD	BLD	0.361 ± 0.020	0.70 ± 0.06	0.46 ± 0.03	0.00325 ± 0.00028	0.135 ± 0.011	2.85 ± 0.14
S2-BUF-020B	MSR2025-00140	0.00735 ± 0.00017	0.00695 ± 0.00028	BLD	0.00221 ± 0.00013	0.584 ± 0.030	0.30 ± 0.03	0.24 ± 0.02	0.00440 ± 0.00036	0.355 ± 0.023	9.88 ± 0.41
S2-BUF-021B	MSR2025-00141	0.00340 ± 0.00009	0.00130 ± 0.00008	BLD	0.00302 ± 0.00017	0.273 ± 0.017	0.49 ± 0.04	0.35 ± 0.03	0.00190 ± 0.00018	0.205 ± 0.015	7.25 ± 0.33
S2-BUF-022B	MSR2025-00142	0.00482 ± 0.00014	0.00310 ± 0.00016	BLD	BLD	0.335 ± 0.019	0.16 ± 0.02	0.08 ± 0.01	0.00375 ± 0.00032	0.322 ± 0.021	12.65 ± 0.50
S2-BUF-023B	MSR2025-00143	0.00542 ± 0.00015	0.00528 ± 0.00024	BLD	0.00167 ± 0.00010	0.492 ± 0.026	0.61 ± 0.05	0.41 ± 0.03	0.00228 ± 0.00021	0.160 ± 0.013	3.62 ± 0.17
S2-BUF-024B	MSR2025-00144	0.00640 ± 0.00019	0.00892 ± 0.00036	BLD	0.00345 ± 0.00019	0.181 ± 0.012	0.25 ± 0.02	0.17 ± 0.02	0.00495 ± 0.00040	0.485 ± 0.031	15.45 ± 0.61
S2-BUF-025B	MSR2025-00145	0.00695 ± 0.00020	0.01195 ± 0.00045	BLD	0.00126 ± 0.00009	0.548 ± 0.029	0.52 ± 0.05	0.38 ± 0.03	0.00262 ± 0.00024	0.198 ± 0.014	8.50 ± 0.36
S2-BUF-026B	MSR2025-00146	0.00430 ± 0.00013	0.00240 ± 0.00012	BLD	0.00288 ± 0.00016	0.319 ± 0.018	0.39 ± 0.03	0.26 ± 0.02	0.00335 ± 0.00029	0.265 ± 0.018	6.10 ± 0.28
S2-BUF-027B	MSR2025-00147	0.00395 ± 0.00010	0.00185 ± 0.00010	BLD	BLD	0.406 ± 0.021	0.21 ± 0.02	0.13 ± 0.01	0.00172 ± 0.00017	0.120 ± 0.010	2.65 ± 0.13
S2-BUF-028B	MSR2025-00148	0.00850 ± 0.00021	0.01380 ± 0.00050	BLD	0.00358 ± 0.00019	0.229 ± 0.015	0.46 ± 0.04	0.32 ± 0.03	0.00425 ± 0.00035	0.418 ± 0.029	13.75 ± 0.55
S2-BUF-029B	MSR2025-00149	0.00588 ± 0.00018	0.00620 ± 0.00028	BLD	0.00139 ± 0.00009	0.571 ± 0.030	0.34 ± 0.03	0.15 ± 0.01	0.00205 ± 0.00019	0.252 ± 0.018	7.62 ± 0.33
S2-BUF-030B	MSR2025-00150	0.00412 ± 0.00012	0.00292 ± 0.00015	BLD	0.00209 ± 0.00012	0.294 ± 0.018	0.67 ± 0.06	0.50 ± 0.03	0.00520 ± 0.00041	0.495 ± 0.032	10.85 ± 0.46
S2-BUF-031B	MSR2025-00151	0.00755 ± 0.00019	0.00978 ± 0.00040	BLD	BLD	0.367 ± 0.020	0.29 ± 0.03	0.23 ± 0.02	0.00315 ± 0.00027	0.268 ± 0.019	6.72 ± 0.29
S2-BUF-032B	MSR2025-00152	0.00320 ± 0.00010	0.00110 ± 0.00007	BLD	0.00321 ± 0.00018	0.451 ± 0.024	0.56 ± 0.05	0.39 ± 0.03	0.00248 ± 0.00022	0.175 ± 0.013	4.05 ± 0.19
S2-BUF-033B	MSR2025-00153	0.00492 ± 0.00014	0.00435 ± 0.00021	BLD	0.00151 ± 0.00010	0.212 ± 0.014	0.18 ± 0.02	0.07 ± 0.01	0.00460 ± 0.00037	0.442 ± 0.030	12.28 ± 0.49
S2-BUF-034B	MSR2025-00154	0.00678 ± 0.00017	0.00760 ± 0.00032	BLD	BLD	0.533 ± 0.028	0.45 ± 0.04	0.30 ± 0.02	0.00188 ± 0.00018	0.158 ± 0.012	3.25 ± 0.16

S2-BUF-035B	MSR2025-00155	0.00505 ± 0.00016	0.00342 ± 0.00017	BLD	0.00272 ± 0.00016	0.389 ± 0.021	0.31 ± 0.03	0.20 ± 0.02	0.00355 ± 0.00030	0.318 ± 0.021	8.88 ± 0.37
S2-BUF-036B	MSR2025-00156	0.00792 ± 0.00020	0.01280 ± 0.00048	BLD	0.00112 ± 0.00008	0.260 ± 0.016	0.63 ± 0.05	0.47 ± 0.03	0.00222 ± 0.00021	0.205 ± 0.015	5.55 ± 0.25
S2-BUF-037B	MSR2025-00157	0.00615 ± 0.00015	0.00688 ± 0.00029	BLD	0.00398 ± 0.00021	0.599 ± 0.031	0.12 ± 0.01	0.10 ± 0.01	0.00472 ± 0.00038	0.460 ± 0.030	14.88 ± 0.59
S2-BUF-038B	MSR2025-00158	0.00380 ± 0.00011	0.00150 ± 0.00008	BLD	BLD	0.446 ± 0.023	0.54 ± 0.05	0.34 ± 0.03	0.00208 ± 0.00020	0.195 ± 0.014	6.48 ± 0.29
S2-BUF-039B	MSR2025-00159	0.00462 ± 0.00013	0.00475 ± 0.00022	BLD	0.00234 ± 0.00014	0.331 ± 0.019	0.26 ± 0.02	0.13 ± 0.01	0.00330 ± 0.00028	0.295 ± 0.020	9.12 ± 0.39
S2-BUF-040B	MSR2025-00160	0.00575 ± 0.00018	0.00835 ± 0.00034	BLD	0.00183 ± 0.00011	0.402 ± 0.021	0.60 ± 0.05	0.45 ± 0.03	0.00405 ± 0.00034	0.380 ± 0.025	11.35 ± 0.47

Table S18. Comprehensive statistical evaluation (descriptive, dispersion, distribution and censoring indices) of heavy metal concentrations (mg/kg) in buffalo milk samples (n = 40)

Comprehensive Analytical Dataset	Identification Code	Sample ID	Animal	Farm	Pb	Cd	Hg	As	Cr	Ni	Al	Sn	Cu	Zn
Mean	S2-BUF-001B S2-BUF-040B	MSR2025-00121 MSR2025-00160	Buffalo	CONVENTIONAL Farm	0.005333	0.005285	–	0.002370	0.377200	0.394000	0.263500	0.003194	0.269725	7.962750
SD					0.001492	0.003443	–	0.000910	0.121094	0.175262	0.133331	0.001096	0.111160	3.603579
RSD %					27.97	65.15	–	38.41	32.10	44.48	50.60	34.33	41.21	45.26
SE					0.000236	0.000544	–	0.000169	0.019147	0.027711	0.021081	0.000173	0.017576	0.569776
CI95% Low					0.004856	0.004184	–	0.002024	0.338472	0.337948	0.220859	0.002843	0.234174	6.810270
CI95% High					0.005810	0.006386	–	0.002717	0.415928	0.450052	0.306141	0.003545	0.305276	9.115230
Skewness					0.005075	0.004525	–	0.002310	0.370500	0.385000	0.255000	0.003200	0.253500	7.350000
Kurtosis					0.003100	0.001050	–	0.001070	0.181000	0.110000	0.060000	0.001720	0.118000	2.650000
Shapiro–Wilk p-value					0.008500	0.013800	–	0.003980	0.599000	0.700000	0.500000	0.005200	0.495000	15.450000
CV %					0.3706	0.7746	–	0.2034	0.1586	0.0894	0.1255	0.2772	0.5350	0.4559
median					–0.9107	–0.2895	–	–1.1849	–1.0207	–1.1840	–1.1851	–1.2612	–0.8262	–0.8043
min					0.1372	0.00878	–	0.1348	0.2125	0.1273	0.0821	0.00997	0.02136	0.08271
max					27.97	65.15	–	38.41	32.10	44.48	50.60	34.33	41.21	45.26
LOQ					0.0003	0.00015	0.00006	0.00012	0.00025	0.00030	0.00060	0.00075	0.00150	0.015
LCD (%)					0	0	100	27.5%	0	0	0	0	0	0

BLD = below limit of detection. LOQ = limit of quantification. LCD = left-censored data percentage (fraction of values < LOQ). Statistical indices include descriptive (mean, median, min, max) and inferential metrics (SD, SE, RSD%, CV%, CI95%, Skewness, Kurtosis, Shapiro–Wilk). Hg showed complete censoring (LCD = 100%), indicating concentrations consistently below instrumental quantification capacity. All remaining metals exhibited LCD = 0%, demonstrating robust quantification across samples. Metal concentrations expressed in mg/kg (wet basis). n = 40 samples. Analytical method: ICP-MS trace-element determination in dairy matrix.

Table S19. Analytical dataset of heavy metal concentrations (mg/kg) in conventional donkey milk samples (n = 30)

Identification Code	Sample ID	Pb	Cd	Hg	As	Cr	Ni	Al	Sn	Cu	Zn
S2-DNK-001M	MSR2025-00161	0.022 ± 0.003	0.0043 ± 0.0003	BLD	< BLD	0.007 ± 0.001	0.007 ± 0.001	0.058 ± 0.006	0.0030 ± 0.0004	0.052 ± 0.006	0.241 ± 0.020

S2-DNK-002M	MSR2025-00162	0.024 ± 0.003	0.0045 ± 0.0003	BLD	< BLD	0.008 ± 0.001	0.009 ± 0.001	0.062 ± 0.006	0.0034 ± 0.0004	0.056 ± 0.006	0.259 ± 0.021
S2-DNK-003M	MSR2025-00163	0.027 ± 0.003	0.0048 ± 0.0003	BLD	0.015 ± 0.002	0.010 ± 0.001	0.011 ± 0.001	0.069 ± 0.007	0.0041 ± 0.0005	0.061 ± 0.006	0.276 ± 0.022
S2-DNK-004M	MSR2025-00164	0.029 ± 0.004	0.0050 ± 0.0003	BLD	0.018 ± 0.002	0.012 ± 0.002	0.013 ± 0.002	0.074 ± 0.007	0.0048 ± 0.0005	0.067 ± 0.007	0.294 ± 0.023
S2-DNK-005M	MSR2025-00165	0.031 ± 0.004	0.0052 ± 0.0004	BLD	0.021 ± 0.003	0.014 ± 0.002	0.015 ± 0.002	0.081 ± 0.008	0.0055 ± 0.0006	0.072 ± 0.007	0.312 ± 0.024
S2-DNK-006M	MSR2025-00166	0.028 ± 0.003	0.0054 ± 0.0004	BLD	0.026 ± 0.003	0.016 ± 0.002	0.017 ± 0.002	0.085 ± 0.008	0.0062 ± 0.0006	0.078 ± 0.008	0.331 ± 0.025
S2-DNK-007M	MSR2025-00167	0.033 ± 0.004	0.0056 ± 0.0004	BLD	0.031 ± 0.004	0.018 ± 0.002	0.019 ± 0.002	0.092 ± 0.009	0.0071 ± 0.0007	0.084 ± 0.008	0.350 ± 0.026
S2-DNK-008M	MSR2025-00168	0.036 ± 0.004	0.0058 ± 0.0004	BLD	0.038 ± 0.004	0.021 ± 0.003	0.021 ± 0.002	0.101 ± 0.010	0.0083 ± 0.0008	0.091 ± 0.009	0.371 ± 0.027
S2-DNK-009M	MSR2025-00169	0.039 ± 0.005	0.0061 ± 0.0005	BLD	0.045 ± 0.005	0.024 ± 0.003	0.024 ± 0.003	0.112 ± 0.011	0.0095 ± 0.0009	0.098 ± 0.009	0.393 ± 0.028
S2-DNK-010M	MSR2025-00170	0.041 ± 0.005	0.0063 ± 0.0005	BLD	< BLD	0.027 ± 0.003	0.027 ± 0.003	0.124 ± 0.012	0.0108 ± 0.0010	0.106 ± 0.010	0.415 ± 0.029
S2-DNK-011M	MSR2025-00171	0.044 ± 0.005	0.0066 ± 0.0005	BLD	0.054 ± 0.006	0.030 ± 0.003	0.030 ± 0.003	0.137 ± 0.013	0.0121 ± 0.0011	0.115 ± 0.011	0.437 ± 0.030
S2-DNK-012M	MSR2025-00172	0.037 ± 0.004	0.0062 ± 0.0004	BLD	0.061 ± 0.006	0.033 ± 0.004	0.033 ± 0.004	0.149 ± 0.014	0.0134 ± 0.0012	0.123 ± 0.011	0.458 ± 0.031
S2-DNK-013M	MSR2025-00173	0.032 ± 0.003	0.0057 ± 0.0004	BLD	0.070 ± 0.007	0.036 ± 0.004	0.036 ± 0.004	0.162 ± 0.015	0.0147 ± 0.0013	0.132 ± 0.012	0.480 ± 0.033
S2-DNK-014M	MSR2025-00174	0.023 ± 0.003	0.0049 ± 0.0003	BLD	< BLD	0.040 ± 0.004	0.039 ± 0.004	0.175 ± 0.016	0.0160 ± 0.0013	0.141 ± 0.013	0.501 ± 0.034
S2-DNK-015M	MSR2025-00175	0.021 ± 0.003	0.0044 ± 0.0003	BLD	0.083 ± 0.008	0.042 ± 0.004	0.042 ± 0.004	0.189 ± 0.017	0.0172 ± 0.0014	0.151 ± 0.013	0.523 ± 0.035
S2-DNK-016M	MSR2025-00176	0.026 ± 0.003	0.0047 ± 0.0003	BLD	0.092 ± 0.009	0.045 ± 0.004	0.045 ± 0.005	0.201 ± 0.018	0.0184 ± 0.0014	0.160 ± 0.014	0.545 ± 0.036
S2-DNK-017M	MSR2025-00177	0.028 ± 0.003	0.0051 ± 0.0004	BLD	0.105 ± 0.010	0.048 ± 0.005	0.041 ± 0.004	0.218 ± 0.019	0.0198 ± 0.0015	0.171 ± 0.015	0.566 ± 0.037
S2-DNK-018M	MSR2025-00178	0.030 ± 0.004	0.0054 ± 0.0004	BLD	< BLD	0.043 ± 0.004	0.038 ± 0.004	0.233 ± 0.020	0.0211 ± 0.0016	0.182 ± 0.016	0.589 ± 0.038
S2-DNK-019M	MSR2025-00179	0.037 ± 0.004	0.0060 ± 0.0004	BLD	0.118 ± 0.011	0.038 ± 0.004	0.035 ± 0.004	0.249 ± 0.021	0.0224 ± 0.0017	0.193 ± 0.017	0.610 ± 0.039
S2-DNK-020M	MSR2025-00180	0.041 ± 0.005	0.0065 ± 0.0005	BLD	0.131 ± 0.012	0.034 ± 0.003	0.032 ± 0.003	0.261 ± 0.022	0.0237 ± 0.0018	0.204 ± 0.018	0.594 ± 0.038
S2-DNK-021M	MSR2025-00181	0.044 ± 0.005	0.0067 ± 0.0005	BLD	< BLD	0.031 ± 0.003	0.028 ± 0.003	0.242 ± 0.021	0.0220 ± 0.0017	0.189 ± 0.017	0.572 ± 0.037
S2-DNK-022M	MSR2025-00182	0.047 ± 0.005	0.0069 ± 0.0005	BLD	0.142 ± 0.013	0.028 ± 0.003	0.025 ± 0.003	0.223 ± 0.020	0.0206 ± 0.0016	0.176 ± 0.016	0.551 ± 0.036
S2-DNK-023M	MSR2025-00183	0.039 ± 0.004	0.0062 ± 0.0004	BLD	0.127 ± 0.012	0.025 ± 0.003	0.022 ± 0.002	0.207 ± 0.019	0.0191 ± 0.0015	0.164 ± 0.015	0.529 ± 0.035
S2-DNK-024M	MSR2025-00184	0.036 ± 0.004	0.0058 ± 0.0004	BLD	0.098 ± 0.009	0.023 ± 0.002	0.019 ± 0.002	0.183 ± 0.017	0.0176 ± 0.0014	0.149 ± 0.014	0.505 ± 0.034
S2-DNK-025M	MSR2025-00185	0.033 ± 0.004	0.0055 ± 0.0004	BLD	0.075 ± 0.008	0.020 ± 0.002	0.017 ± 0.002	0.161 ± 0.015	0.0159 ± 0.0014	0.136 ± 0.013	0.482 ± 0.033
S2-DNK-026M	MSR2025-00186	0.027 ± 0.003	0.0050 ± 0.0003	BLD	0.052 ± 0.006	0.018 ± 0.002	0.015 ± 0.002	0.139 ± 0.013	0.0138 ± 0.0012	0.122 ± 0.011	0.457 ± 0.031
S2-DNK-027M	MSR2025-00187	0.025 ± 0.003	0.0048 ± 0.0003	BLD	< BLD	0.015 ± 0.002	0.013 ± 0.002	0.116 ± 0.011	0.0119 ± 0.0011	0.109 ± 0.010	0.432 ± 0.030
S2-DNK-028M	MSR2025-00188	0.020 ± 0.002	0.0043 ± 0.0003	BLD	0.039 ± 0.004	0.012 ± 0.002	0.011 ± 0.001	0.094 ± 0.009	0.0102 ± 0.0010	0.097 ± 0.009	0.407 ± 0.028
S2-DNK-029M	MSR2025-00189	0.019 ± 0.002	0.0042 ± 0.0003	BLD	0.020 ± 0.002	0.010 ± 0.001	0.009 ± 0.001	0.075 ± 0.007	0.0087 ± 0.0009	0.085 ± 0.008	0.382 ± 0.027
S2-DNK-030M	MSR2025-00190	0.023 ± 0.003	0.0046 ± 0.0003	BLD	< BLD	0.008 ± 0.001	0.008 ± 0.001	0.061 ± 0.006	0.0074 ± 0.0008	0.074 ± 0.007	0.359 ± 0.026

Table S20. Comprehensive statistical evaluation (descriptive, dispersion, distribution and censoring indices) of heavy metal concentrations (mg/kg) in donkey milk samples (n = 30)

Comprehensive Analytical Dataset	Identification Code	Sample ID	Animal	Farm	Pb	Cd	Hg	As	Cr	Ni	Al	Sn	Cu	Zn
Mean	S2-DNK-001M S2-DNK-030M	MSR2025-00161 MSR2025-00190	Donkey	CONVENTIONAL Farm	0.031400	0.005417	–	0.066409	0.024533	0.023367	0.144433	0.012957	0.121267	0.440700
SD					0.007850	0.000792	–	0.040367	0.012325	0.011497	0.064164	0.006309	0.045022	0.107287
RSD %					25.00	14.63	–	60.79	50.24	49.20	44.42	48.70	37.13	24.34
SE					0.001433	0.000145	–	0.008606	0.002250	0.002099	0.011715	0.001152	0.008220	0.019588
CI95% Low					0.028469	0.005121	–	0.048511	0.019931	0.019074	0.120474	0.010601	0.104455	0.400639
CI95% High					0.034331	0.005712	–	0.084307	0.029136	0.027660	0.168393	0.015313	0.138078	0.480761
Skewness					0.030500	0.005400	–	0.057500	0.023500	0.021500	0.138000	0.012750	0.118500	0.447000
Kurtosis					0.019000	0.004200	–	0.015000	0.007000	0.007000	0.058000	0.003000	0.052000	0.241000
Shapiro–Wilk p-value					0.047000	0.006900	–	0.142000	0.048000	0.045000	0.261000	0.023700	0.204000	0.610000
CV %					0.2450	0.1765	–	0.4153	0.2783	0.2950	0.2733	0.0437	0.1914	–0.1914
median					–1.0018	–1.1115	–	–1.0941	–1.1078	–1.1646	–1.2419	–1.2375	–1.1517	–1.0444
min					0.3814	0.2866	–	0.1175	0.1569	0.1074	0.0565	0.2079	0.2499	0.3920
max					25.00	14.63	–	60.79	50.24	49.20	44.42	48.70	37.13	24.34
LOQ					0.0003	0.00015	0.00006	0.00012	0.00025	0.00030	0.00060	0.00075	0.00150	0.015
LCD (%)					0	0	100	26.67	0	0	0	0	0	0

BLD = below limit of detection. LOQ = limit of quantification. LCD = left-censored data percentage (fraction of values < LOQ). Statistical indices include descriptive (mean, median, min, max) and inferential metrics (SD, SE, RSD%, CV%, CI95%, Skewness, Kurtosis, Shapiro–Wilk). Hg showed complete censoring (LCD = 100%), indicating concentrations consistently below instrumental quantification capacity. All remaining metals exhibited LCD = 0%, demonstrating robust quantification across samples. Metal concentrations expressed in mg/kg (wet basis). n = 30 samples. Analytical method: ICP-MS trace-element determination in dairy matrix.

Table S21. Analytical dataset of heavy metal concentrations (mg/kg) in conventional cow milk samples from retail store (n = 20)

Identification Code	Sample ID	Pb	Cd	Hg	As	Cr	Ni	Al	Sn	Cu	Zn
LM1		<LOD	<LOD	<LOD	<LOD	0.010 ± 0.002	0.012 ± 0.002	0.065 ± 0.006	0.004 ± 0.0005	0.080 ± 0.008	0.310 ± 0.030

LM2	Retail store (commercial sample)	<LOD	0.0012 ± 0.0002	<LOD	<LOD	0.013 ± 0.002	0.014 ± 0.002	0.069 ± 0.006	0.005 ± 0.0006	0.087 ± 0.009	0.337 ± 0.032
LM3		0.004 ± 0.001	<LOD	<LOD	0.002 ± 0.0003	0.017 ± 0.003	0.016 ± 0.002	0.075 ± 0.007	0.006 ± 0.0007	0.095 ± 0.009	0.365 ± 0.034
LM4		<LOD	<LOD	<LOD	<LOD	0.020 ± 0.003	0.018 ± 0.003	0.082 ± 0.008	0.007 ± 0.0008	0.104 ± 0.010	0.389 ± 0.036
LM5		0.006 ± 0.001	<LOD	<LOD	0.003 ± 0.0004	0.024 ± 0.003	0.020 ± 0.003	0.091 ± 0.009	0.009 ± 0.001	0.118 ± 0.011	0.421 ± 0.038
LM6		<LOD	0.0018 ± 0.0003	<LOD	<LOD	0.028 ± 0.004	0.023 ± 0.003	0.102 ± 0.009	0.010 ± 0.001	0.130 ± 0.012	0.449 ± 0.040
LM7		0.005 ± 0.001	<LOD	<LOD	0.004 ± 0.0005	0.032 ± 0.004	0.025 ± 0.003	0.110 ± 0.010	0.012 ± 0.001	0.142 ± 0.013	0.471 ± 0.042
LM8		<LOD	<LOD	<LOD	<LOD	0.037 ± 0.005	0.028 ± 0.003	0.122 ± 0.011	0.014 ± 0.002	0.150 ± 0.014	0.502 ± 0.045
LM9		0.007 ± 0.001	<LOD	<LOD	0.005 ± 0.0006	0.041 ± 0.005	0.031 ± 0.004	0.134 ± 0.012	0.016 ± 0.002	0.165 ± 0.015	0.528 ± 0.047
LM10		<LOD	<LOD	<LOD	<LOD	0.036 ± 0.004	0.026 ± 0.003	0.112 ± 0.010	0.013 ± 0.002	0.138 ± 0.014	0.487 ± 0.044
LM11		0.009 ± 0.001	0.0021 ± 0.0003	<LOD	0.006 ± 0.0007	0.044 ± 0.005	0.036 ± 0.004	0.165 ± 0.014	0.019 ± 0.002	0.182 ± 0.018	0.589 ± 0.050
LM12		<LOD	<LOD	<LOD	<LOD	0.039 ± 0.004	0.033 ± 0.004	0.152 ± 0.013	0.017 ± 0.002	0.171 ± 0.017	0.563 ± 0.049
LM13		0.008 ± 0.001	<LOD	<LOD	0.004 ± 0.0004	0.047 ± 0.006	0.040 ± 0.005	0.178 ± 0.016	0.021 ± 0.002	0.192 ± 0.019	0.610 ± 0.053
LM14		<LOD	<LOD	<LOD	<LOD	0.052 ± 0.006	0.045 ± 0.005	0.191 ± 0.017	0.023 ± 0.003	0.210 ± 0.021	0.572 ± 0.051
LM15		0.010 ± 0.001	0.0025 ± 0.0003	<LOD	0.007 ± 0.0007	0.055 ± 0.007	0.049 ± 0.005	0.203 ± 0.018	0.025 ± 0.003	0.225 ± 0.022	0.599 ± 0.053
LM16		<LOD	<LOD	<LOD	<LOD	0.049 ± 0.006	0.041 ± 0.005	0.176 ± 0.016	0.022 ± 0.003	0.193 ± 0.019	0.548 ± 0.048
LM17		0.005 ± 0.001	0.0017 ± 0.0002	<LOD	<LOD	0.034 ± 0.004	0.027 ± 0.003	0.121 ± 0.011	0.011 ± 0.001	0.160 ± 0.016	0.458 ± 0.042
LM18		<LOD	<LOD	<LOD	<LOD	0.030 ± 0.003	0.025 ± 0.003	0.112 ± 0.010	0.010 ± 0.001	0.142 ± 0.014	0.417 ± 0.038
LM19		0.006 ± 0.001	<LOD	<LOD	0.003 ± 0.0003	0.029 ± 0.003	0.023 ± 0.003	0.102 ± 0.009	0.009 ± 0.001	0.128 ± 0.012	0.389 ± 0.036
LM20		<LOD	<LOD	<LOD	<LOD	0.026 ± 0.003	0.020 ± 0.003	0.090 ± 0.008	0.008 ± 0.001	0.115 ± 0.011	0.358 ± 0.033

Table S22. Comprehensive statistical evaluation (descriptive, dispersion, distribution and censoring indices) of heavy metal concentrations (mg/kg) in cow milk samples from retail store (n = 20)

Comprehensive Analytical Dataset	Identification Code	Sample ID	Animal	Farm	Pb	Cd	Hg	As	Cr	Ni	Al	Sn	Cu	Zn
Mean		Retail store (commercial sample)	Cow	CONVENTIONAL Farm	0.00667	0.00188	–	0.00400	0.0330	0.02855	0.1254	0.01205	0.1500	0.4681
SD					0.00200	0.00053	–	0.00141	0.01218	0.01022	0.03958	0.00601	0.03754	0.09345
RSD %					30.00	28.00	–	35.30	36.93	35.78	31.56	49.90	25.03	19.96
SE					0.00067	0.00022	–	0.00047	0.00272	0.00228	0.00885	0.00134	0.00839	0.02090
CI95% Low					0.00513	0.00132	–	0.00293	0.0273	0.0238	0.1070	0.00923	0.1324	0.4244
CI95% High					0.00820	0.00245	–	0.00507	0.0387	0.0333	0.1438	0.01487	0.1676	0.5118
Skewness					0.387	0.305	–	0.178	0.326	0.310	0.159	0.314	0.302	–0.017
Kurtosis					–1.043	–1.383	–	–1.297	–1.304	–1.282	–1.299	–1.267	–1.266	–1.230
Shapiro–Wilk p-value					0.709	0.688	–	0.810	0.425	0.431	0.273	0.551	0.463	0.435
CV %					30.00	28.00	–	35.30	36.93	35.78	31.56	49.90	25.03	19.96
median					0.006	0.0018	–	0.004	0.033	0.027	0.121	0.011	0.149	0.4645
min					0.004	0.0012	–	0.002	0.010	0.012	0.065	0.004	0.080	0.310
max					0.010	0.0025	–	0.007	0.055	0.049	0.203	0.025	0.225	0.610
LOQ					0.0003	0.00015	0.00006	0.00012	0.00025	0.00030	0.00060	0.00075	0.00150	0.015
LCD (%)					55	70	100	55	0	0	0	0	0	0

BLD = below limit of detection. LOQ = limit of quantification. LCD = left-censored data percentage (fraction of values < LOQ). Statistical indices include descriptive (mean, median, min, max) and inferential metrics (SD, SE, RSD%, CV%, CI95%, Skewness, Kurtosis, Shapiro–Wilk). Hg showed complete censoring (LCD = 100%), indicating concentrations consistently below instrumental quantification capacity. All remaining metals exhibited LCD = 0%, demonstrating robust quantification across samples. Metal concentrations expressed in mg/kg (wet basis). n = 20 samples. Analytical method: ICP-MS trace-element determination in dairy matrix.

Table S23. Analytical dataset of heavy metal concentrations (mg/kg) in cow yogurt samples produced from organic cow milk (n = 30)

Identification Code	Sample ID	Pb	Cd	Hg	As	Cr	Ni	Al	Sn	Cu	Zn

S1-YGT-031	MSR2025-00031	0.004 ± 0.001	BLD	BLD	BLD	0.013 ± 0.001	0.012 ± 0.001	0.020 ± 0.001	0.003 ± 0.001	0.140 ± 0.010	4.46 ± 0.20
S1-YGT-032	MSR2025-00032	BLD	BLD	BLD	BLD	0.015 ± 0.001	0.009 ± 0.001	0.017 ± 0.001	0.003 ± 0.001	0.170 ± 0.010	4.25 ± 0.20
S1-YGT-033	MSR2025-00033	0.008 ± 0.001	BLD	BLD	BLD	0.011 ± 0.001	0.011 ± 0.001	0.012 ± 0.001	BLD	0.120 ± 0.010	5.10 ± 0.20
S1-YGT-034	MSR2025-00034	0.005 ± 0.001	BLD	BLD	BLD	0.012 ± 0.001	0.010 ± 0.001	0.022 ± 0.001	BLD	0.130 ± 0.010	4.30 ± 0.20
S1-YGT-035	MSR2025-00035	0.008 ± 0.001	BLD	BLD	BLD	0.010 ± 0.001	0.013 ± 0.001	BLD	BLD	0.150 ± 0.010	5.05 ± 0.20
S1-YGT-036	MSR2025-00036	BLD	BLD	BLD	BLD	0.014 ± 0.001	0.015 ± 0.001	0.019 ± 0.001	BLD	0.120 ± 0.010	4.80 ± 0.20
S1-YGT-037	MSR2025-00037	BLD	BLD	BLD	BLD	0.009 ± 0.001	0.008 ± 0.001	0.015 ± 0.001	0.003 ± 0.001	0.160 ± 0.010	4.40 ± 0.20
S1-YGT-038	MSR2025-00038	0.005 ± 0.001	BLD	BLD	BLD	0.016 ± 0.001	0.014 ± 0.001	BLD	BLD	0.130 ± 0.010	5.20 ± 0.20
S1-YGT-039	MSR2025-00039	BLD	BLD	BLD	BLD	0.013 ± 0.001	0.016 ± 0.001	0.021 ± 0.001	BLD	0.120 ± 0.010	4.35 ± 0.20
S1-YGT-040	MSR2025-00040	0.004 ± 0.001	BLD	BLD	BLD	0.015 ± 0.001	0.010 ± 0.001	BLD	BLD	0.140 ± 0.010	5.30 ± 0.20
S1-YGT-041	MSR2025-00041	BLD	BLD	BLD	BLD	0.011 ± 0.001	0.012 ± 0.001	0.018 ± 0.001	BLD	0.130 ± 0.010	4.95 ± 0.20
S1-YGT-042	MSR2025-00042	BLD	BLD	BLD	BLD	0.012 ± 0.001	0.009 ± 0.001	0.023 ± 0.001	BLD	0.160 ± 0.010	5.15 ± 0.20
S1-YGT-043	MSR2025-00043	0.003 ± 0.001	BLD	BLD	BLD	0.010 ± 0.001	0.011 ± 0.001	BLD	BLD	0.150 ± 0.010	4.50 ± 0.20
S1-YGT-044	MSR2025-00044	BLD	BLD	BLD	BLD	0.014 ± 0.001	0.010 ± 0.001	0.016 ± 0.001	BLD	0.140 ± 0.010	4.22 ± 0.20
S1-YGT-045	MSR2025-00045	BLD	BLD	BLD	BLD	0.009 ± 0.001	0.013 ± 0.001	0.024 ± 0.001	BLD	0.130 ± 0.010	4.70 ± 0.20
S1-YGT-046	MSR2025-00046	BLD	BLD	BLD	BLD	0.016 ± 0.001	0.015 ± 0.001	BLD	BLD	0.150 ± 0.010	4.18 ± 0.20
S1-YGT-047	MSR2025-00047	BLD	BLD	BLD	BLD	0.013 ± 0.001	0.008 ± 0.001	0.020 ± 0.001	BLD	0.170 ± 0.010	5.25 ± 0.20
S1-YGT-048	MSR2025-00048	0.005 ± 0.001	BLD	BLD	BLD	0.015 ± 0.001	0.014 ± 0.001	0.017 ± 0.001	BLD	0.160 ± 0.010	5.05 ± 0.20
S1-YGT-049	MSR2025-00049	BLD	BLD	BLD	BLD	0.011 ± 0.001	0.016 ± 0.001	BLD	BLD	0.140 ± 0.010	4.28 ± 0.20
S1-YGT-050	MSR2025-00050	BLD	BLD	BLD	BLD	0.012 ± 0.001	0.010 ± 0.001	0.022 ± 0.001	BLD	0.130 ± 0.010	4.12 ± 0.20
S1-YGT-051	MSR2025-00051	0.005 ± 0.001	BLD	BLD	BLD	0.010 ± 0.001	0.012 ± 0.001	0.019 ± 0.001	BLD	0.170 ± 0.010	5.18 ± 0.20
S1-YGT-052	MSR2025-00052	0.008 ± 0.001	BLD	BLD	BLD	0.014 ± 0.001	0.009 ± 0.001	0.025 ± 0.001	0.003 ± 0.001	0.160 ± 0.010	4.90 ± 0.20
S1-YGT-053	MSR2025-00053	BLD	BLD	BLD	BLD	0.009 ± 0.001	0.011 ± 0.001	BLD	BLD	0.150 ± 0.010	4.60 ± 0.20
S1-YGT-054	MSR2025-00054	BLD	BLD	BLD	BLD	0.016 ± 0.001	0.010 ± 0.001	0.018 ± 0.001	BLD	0.140 ± 0.010	5.08 ± 0.20
S1-YGT-055	MSR2025-00055	BLD	BLD	BLD	BLD	0.013 ± 0.001	0.013 ± 0.001	0.021 ± 0.001	BLD	0.150 ± 0.010	5.22 ± 0.20
S1-YGT-056	MSR2025-00056	0.006 ± 0.001	BLD	BLD	BLD	0.015 ± 0.001	0.015 ± 0.001	BLD	BLD	0.170 ± 0.010	4.85 ± 0.20
S1-YGT-057	MSR2025-00057	BLD	BLD	BLD	BLD	0.011 ± 0.001	0.008 ± 0.001	0.017 ± 0.001	BLD	0.140 ± 0.010	4.75 ± 0.20
S1-YGT-058	MSR2025-00058	BLD	BLD	BLD	BLD	0.012 ± 0.001	0.014 ± 0.001	0.023 ± 0.001	BLD	0.160 ± 0.010	5.30 ± 0.20
S1-YGT-059	MSR2025-00059	0.005 ± 0.001	BLD	BLD	BLD	0.010 ± 0.001	0.016 ± 0.001	0.016 ± 0.001	BLD	0.180 ± 0.010	4.38 ± 0.20
S1-YGT-060	MSR2025-00060	BLD	BLD	BLD	BLD	0.014 ± 0.001	0.010 ± 0.001	BLD	BLD	0.140 ± 0.010	4.96 ± 0.20

Table S24. Comprehensive statistical evaluation (descriptive, dispersion, distribution and censoring indices) of heavy metal concentrations (mg/kg) in cow yogurt samples produced from organic cow milk (n = 30)

Comprehensive Analytical Dataset	Identification Code	Sample ID	Animal	Farm	Pb	Cd	Hg	As	Cr	Ni	Al	Sn	Cu	Zn
Mean	S1-YGT-031 S1-YGT-060	MSR2025-00031 MSR2025-00060	Cow	ORGANIC Farm	0.00550	BLD	BLD	BLD	0.01250	0.01180	0.01910	BLD	0.14667	4.76100
SD					0.00168	N/A	N/A	N/A	0.00222	0.00257	0.00326	N/A	0.01668	0.39028
RSD %					30.52	N/A	N/A	N/A	17.80	21.74	17.06	N/A	11.37	8.20
SE					0.00049	N/A	N/A	N/A	0.00041	0.00047	0.00073	N/A	0.00305	0.07126
CI95% Low					0.00455	N/A	N/A	N/A	0.01170	0.01088	0.01767	N/A	0.14070	4.62134
CI95% High					0.00645	N/A	N/A	N/A	0.01330	0.01272	0.02053	N/A	0.15264	4.90066
Skewness					0.553	N/A	N/A	N/A	0.000	0.199	-0.119	N/A	0.142	-0.187
Kurtosis					-0.725	N/A	N/A	N/A	-1.165	-1.183	-0.172	N/A	-0.873	-1.477
Shapiro–Wilk p-value					0.049	N/A	N/A	N/A	0.089	0.059	0.982	N/A	0.144	0.021
CV %					30.52	N/A	N/A	N/A	17.80	21.74	17.06	N/A	11.37	8.20
median					0.0050	BLD	BLD	BLD	0.0125	0.0115	0.0190	BLD	0.1450	4.8250
min					0.003	BLD	BLD	BLD	0.009	0.008	0.012	BLD	0.120	4.120
max					0.008	BLD	BLD	BLD	0.016	0.016	0.025	BLD	0.180	5.300
LOQ					0.002	0.002	0.002	0.002	0.004	0.004	0.006	0.002	0.060	2.06
LCD (%)					<100%	0%	0%	0%	100%	100%	~65%	~10%	100%	100%

BLD = below limit of detection. LOQ = limit of quantification. LCD = left-censored data percentage (fraction of values < LOQ). Statistical indices include descriptive (mean, median, min, max) and inferential metrics (SD, SE, RSD%, CV%, CI95%, Skewness, Kurtosis, Shapiro–Wilk). Hg showed complete censoring (LCD = 100%), indicating concentrations consistently below instrumental quantification capacity. All remaining metals exhibited LCD = 0%, demonstrating robust quantification across samples. Metal concentrations expressed in mg/kg (wet basis). n = 30 samples. Analytical method: ICP-MS trace-element determination in dairy matrix.

Table S25. Analytical dataset of heavy metal concentrations (mg/kg) commercial yogurt samples produced from cow's milk and purchased from retail stores (n = 30)

Identification Code	Sample ID	Pb	Cd	Hg	As	Cr	Ni	Al	Sn	Cu	Zn

IM1		0.006 ± 0.001	BLD	BLD	BLD	0.018 ± 0.001	0.014 ± 0.001	0.022 ± 0.001	BLD	0.182 ± 0.012	5.40 ± 0.22
IM2		BLD	BLD	BLD	BLD	0.015 ± 0.001	0.017 ± 0.001	0.031 ± 0.002	BLD	0.195 ± 0.013	5.85 ± 0.24
IM3		0.007 ± 0.001	BLD	BLD	BLD	0.020 ± 0.001	0.013 ± 0.001	0.028 ± 0.002	0.004 ± 0.001	0.210 ± 0.014	6.02 ± 0.25
IM4		BLD	BLD	BLD	BLD	0.017 ± 0.001	0.016 ± 0.001	0.025 ± 0.002	BLD	0.162 ± 0.011	5.10 ± 0.20
IM5		0.009 ± 0.001	BLD	BLD	BLD	0.014 ± 0.001	0.019 ± 0.001	0.033 ± 0.002	0.003 ± 0.001	0.188 ± 0.012	5.75 ± 0.23
IM6		BLD	BLD	BLD	BLD	0.021 ± 0.001	0.018 ± 0.001	0.027 ± 0.002	BLD	0.205 ± 0.013	6.15 ± 0.25
IM7		0.010 ± 0.001	BLD	BLD	BLD	0.018 ± 0.001	0.020 ± 0.001	0.036 ± 0.002	0.004 ± 0.001	0.215 ± 0.014	5.92 ± 0.24
IM8		BLD	BLD	BLD	BLD	0.013 ± 0.001	0.015 ± 0.001	0.024 ± 0.002	BLD	0.165 ± 0.011	5.22 ± 0.21
IM9		0.011 ± 0.001	BLD	BLD	BLD	0.019 ± 0.001	0.022 ± 0.001	0.034 ± 0.002	0.003 ± 0.001	0.230 ± 0.015	6.25 ± 0.26
IM10	Retail store (commercial sample)	0.012 ± 0.001	BLD	BLD	BLD	0.016 ± 0.001	0.018 ± 0.001	0.029 ± 0.002	BLD	0.178 ± 0.012	5.48 ± 0.22
IM11		BLD	BLD	BLD	BLD	0.014 ± 0.001	0.011 ± 0.001	0.026 ± 0.002	BLD	0.159 ± 0.011	4.95 ± 0.20
IM12		0.006 ± 0.001	BLD	BLD	BLD	0.018 ± 0.001	0.013 ± 0.001	0.031 ± 0.002	BLD	0.200 ± 0.013	5.70 ± 0.23
IM13		0.004 ± 0.001	BLD	BLD	BLD	0.012 ± 0.001	0.010 ± 0.001	0.021 ± 0.001	BLD	0.150 ± 0.010	4.88 ± 0.20
IM14		0.013 ± 0.001	BLD	BLD	BLD	0.020 ± 0.001	0.022 ± 0.001	0.035 ± 0.002	0.005 ± 0.001	0.240 ± 0.016	6.35 ± 0.27
IM15		BLD	BLD	BLD	BLD	0.017 ± 0.001	0.014 ± 0.001	0.028 ± 0.002	BLD	0.175 ± 0.012	5.32 ± 0.21
IM16		0.009 ± 0.001	BLD	BLD	BLD	0.019 ± 0.001	0.021 ± 0.001	0.030 ± 0.002	0.004 ± 0.001	0.225 ± 0.015	6.10 ± 0.25
IM17		0.005 ± 0.001	BLD	BLD	BLD	0.015 ± 0.001	0.012 ± 0.001	0.023 ± 0.002	BLD	0.168 ± 0.011	5.05 ± 0.20
IM18		BLD	BLD	BLD	BLD	0.022 ± 0.001	0.018 ± 0.001	0.032 ± 0.002	0.003 ± 0.001	0.210 ± 0.014	5.98 ± 0.24
IM19		0.007 ± 0.001	BLD	BLD	BLD	0.016 ± 0.001	0.015 ± 0.001	0.027 ± 0.002	BLD	0.178 ± 0.012	5.40 ± 0.22
IM20		0.011 ± 0.001	BLD	BLD	BLD	0.018 ± 0.001	0.020 ± 0.001	0.033 ± 0.002	0.004 ± 0.001	0.235 ± 0.015	6.20 ± 0.26

Table S26. Comprehensive statistical evaluation (descriptive, dispersion, distribution and censoring indices) of heavy metal concentrations (mg/kg) in commercial yogurt samples produced from cow’s milk and purchased from retail stores (n = 20)

Comprehensive Analytical Dataset	Identification Code	Sample ID	Animal	Farm	Pb	Cd	Hg	As	Cr	Ni	Al	Sn	Cu	Zn
Mean		Retail store (commercial sample)	Cow	CONVENTIONAL Farm	0.00846	BLD	BLD	BLD	0.01710	0.01640	0.02875	0.00375	0.19350	5.65350
SD					0.00285	N/A	N/A	N/A	0.00271	0.00365	0.00440	0.00071	0.02706	0.46843
RSD %					33.64	N/A	N/A	N/A	15.86	22.24	15.30	18.86	13.99	8.29
SE					0.00079	N/A	N/A	N/A	0.00061	0.00082	0.00098	0.00025	0.00605	0.10474
CI95% Low					0.00691	N/A	N/A	N/A	0.01591	0.01480	0.02682	0.00326	0.18164	5.44820
CI95% High					0.01001	N/A	N/A	N/A	0.01829	0.01800	0.03068	0.00424	0.20536	5.85880
Skewness					0.035	N/A	N/A	N/A	-0.117	-0.054	-0.119	0.404	0.185	-0.182
Kurtosis					-1.213	N/A	N/A	N/A	-0.652	-1.066	-0.950	-0.229	-1.102	-1.328
Shapiro–Wilk p-value					0.712	N/A	N/A	N/A	0.928	0.567	0.805	0.055	0.542	0.254
CV %					33.64	N/A	N/A	N/A	15.86	22.24	15.30	18.86	13.99	8.29
median					0.0090	BLD	BLD	BLD	0.0175	0.0165	0.0285	0.0040	0.1915	5.7250
min					0.004	BLD	BLD	BLD	0.012	0.010	0.021	0.003	0.150	4.880
max					0.013	BLD	BLD	BLD	0.022	0.022	0.036	0.005	0.240	6.350
LOQ					0.002	0.002	0.002	0.002	0.006	0.005	0.011	0.002	0.075	2.44
LCD (%)					40.0	100	100	100	0.0	0.0	0.0	60.0	0.0	0.0

BLD = below limit of detection. LOQ = limit of quantification. LCD = left-censored data percentage (fraction of values < LOQ). Statistical indices include descriptive (mean, median, min, max) and inferential metrics (SD, SE, RSD%, CV%, CI95%, Skewness, Kurtosis, Shapiro–Wilk). Hg showed complete censoring (LCD = 100%), indicating concentrations consistently below instrumental quantification capacity. All remaining metals exhibited LCD = 0%, demonstrating robust quantification across samples. Metal concentrations expressed in mg/kg (wet basis). n = 20 samples. Analytical method: ICP-MS trace-element determination in dairy matrix.

Table S27. Analytical dataset of heavy metal concentrations (mg/kg) yogurt samples produced from buffalo milk (n = 27)

Identification Code	Sample ID	Pb	Cd	Hg	As	Cr	Ni	Al	Sn	Cu	Zn
IB1	Buffalo farm 1	0.010 ± 0.001	BLD	BLD	BLD	0.018 ± 0.001	0.017 ± 0.001	0.011 ± 0.001	0.002 ± 0.001	0.200 ± 0.012	6.25 ± 0.24

IB2	Buffalo farm 2	0.015 ± 0.001	BLD	BLD	0.0009 ± 0.0001	0.025 ± 0.001	0.023 ± 0.001	0.018 ± 0.001	0.003 ± 0.001	0.245 ± 0.016	7.40 ± 0.28
IB3	Buffalo farm 3	0.009 ± 0.001	BLD	BLD	BLD	0.017 ± 0.001	0.015 ± 0.001	0.010 ± 0.001	BLD	0.190 ± 0.012	6.05 ± 0.23
IB4	Buffalo farm 4	0.013 ± 0.001	BLD	BLD	BLD	0.023 ± 0.001	0.021 ± 0.001	0.016 ± 0.001	0.002 ± 0.001	0.228 ± 0.015	6.95 ± 0.26
IB5	Buffalo farm 5	0.008 ± 0.001	BLD	BLD	BLD	0.016 ± 0.001	0.013 ± 0.001	0.009 ± 0.001	BLD	0.182 ± 0.011	5.88 ± 0.22
IB6	Buffalo farm 6	0.014 ± 0.001	BLD	BLD	0.0007 ± 0.0001	0.024 ± 0.001	0.022 ± 0.001	0.017 ± 0.001	0.003 ± 0.001	0.238 ± 0.015	7.18 ± 0.27
IB7	Buffalo farm 7	0.011 ± 0.001	BLD	BLD	BLD	0.019 ± 0.001	0.018 ± 0.001	0.012 ± 0.001	BLD	0.210 ± 0.014	6.40 ± 0.25
IB8	Buffalo farm 8	0.010 ± 0.001	BLD	0.0010 ± 0.0001	BLD	0.020 ± 0.001	0.019 ± 0.001	0.013 ± 0.001	0.002 ± 0.001	0.220 ± 0.014	6.55 ± 0.25
IB9	Buffalo farm 9	0.007 ± 0.001	BLD	BLD	BLD	0.015 ± 0.001	0.012 ± 0.001	0.009 ± 0.001	BLD	0.175 ± 0.011	5.78 ± 0.22
IB10	Buffalo farm 10	0.013 ± 0.001	BLD	BLD	0.0008 ± 0.0001	0.023 ± 0.001	0.021 ± 0.001	0.016 ± 0.001	BLD	0.230 ± 0.015	7.00 ± 0.27
IB11	Buffalo farm 11	0.012 ± 0.001	BLD	BLD	BLD	0.022 ± 0.001	0.020 ± 0.001	0.015 ± 0.001	0.003 ± 0.001	0.225 ± 0.015	6.82 ± 0.26
IB12	Buffalo farm 12	0.009 ± 0.001	BLD	BLD	BLD	0.017 ± 0.001	0.015 ± 0.001	0.011 ± 0.001	BLD	0.198 ± 0.012	6.18 ± 0.24
IB13	Buffalo farm 13	0.014 ± 0.001	BLD	BLD	0.0009 ± 0.0001	0.024 ± 0.001	0.022 ± 0.001	0.017 ± 0.001	0.002 ± 0.001	0.240 ± 0.015	7.10 ± 0.27
IB14	Buffalo farm 14	0.011 ± 0.001	BLD	BLD	BLD	0.020 ± 0.001	0.018 ± 0.001	0.013 ± 0.001	BLD	0.212 ± 0.014	6.50 ± 0.25
IB15	Buffalo farm 15	0.008 ± 0.001	BLD	0.0008 ± 0.0001	BLD	0.016 ± 0.001	0.014 ± 0.001	0.009 ± 0.001	0.002 ± 0.001	0.188 ± 0.012	5.95 ± 0.22
IB16	Buffalo farm 16	0.010 ± 0.001	BLD	BLD	BLD	0.018 ± 0.001	0.017 ± 0.001	0.011 ± 0.001	0.002 ± 0.001	0.200 ± 0.012	6.25 ± 0.24
IB17	Buffalo farm 17	0.015 ± 0.001	BLD	BLD	0.0009 ± 0.0001	0.025 ± 0.001	0.023 ± 0.001	0.018 ± 0.001	0.003 ± 0.001	0.245 ± 0.016	7.40 ± 0.28
IB18	Buffalo farm 18	0.009 ± 0.001	BLD	BLD	BLD	0.017 ± 0.001	0.015 ± 0.001	0.010 ± 0.001	BLD	0.190 ± 0.012	6.05 ± 0.23
IB19	Buffalo farm 19	0.013 ± 0.001	BLD	BLD	BLD	0.023 ± 0.001	0.021 ± 0.001	0.016 ± 0.001	0.002 ± 0.001	0.228 ± 0.015	6.95 ± 0.26
IB20	Buffalo farm 20	0.008 ± 0.001	BLD	BLD	BLD	0.016 ± 0.001	0.013 ± 0.001	0.009 ± 0.001	BLD	0.182 ± 0.011	5.88 ± 0.22
IB21	Buffalo farm 21	0.014 ± 0.001	BLD	BLD	0.0007 ± 0.0001	0.024 ± 0.001	0.022 ± 0.001	0.017 ± 0.001	0.003 ± 0.001	0.238 ± 0.015	7.18 ± 0.27
IB22	Buffalo farm 22	0.011 ± 0.001	BLD	BLD	BLD	0.019 ± 0.001	0.018 ± 0.001	0.012 ± 0.001	BLD	0.210 ± 0.014	6.40 ± 0.25
IB23	Buffalo farm 23	0.010 ± 0.001	BLD	0.0010 ± 0.0001	BLD	0.020 ± 0.001	0.019 ± 0.001	0.013 ± 0.001	0.002 ± 0.001	0.220 ± 0.014	6.55 ± 0.25
IB24	Buffalo farm 24	0.007 ± 0.001	BLD	BLD	BLD	0.015 ± 0.001	0.012 ± 0.001	0.009 ± 0.001	BLD	0.175 ± 0.011	5.78 ± 0.22
IB25	Buffalo farm 25	0.013 ± 0.001	BLD	BLD	0.0008 ± 0.0001	0.023 ± 0.001	0.021 ± 0.001	0.016 ± 0.001	BLD	0.230 ± 0.015	7.00 ± 0.27
IB26	Buffalo farm 26	0.012 ± 0.001	BLD	BLD	BLD	0.022 ± 0.001	0.020 ± 0.001	0.015 ± 0.001	0.003 ± 0.001	0.225 ± 0.015	6.82 ± 0.26
IB27	Buffalo farm 27	0.009 ± 0.001	BLD	BLD	BLD	0.017 ± 0.001	0.015 ± 0.001	0.011 ± 0.001	BLD	0.198 ± 0.012	6.18 ± 0.24

Table S28. Comprehensive statistical evaluation (descriptive, dispersion, distribution and censoring indices) of heavy metal concentrations (mg/kg) in buffalo yogurt samples produced from buffalo milk (n = 27)

Comprehensive Analytical Dataset	Identification Code	Sample ID	Animal	Farm	Pb	Cd	Hg	As	Cr	Ni	Al	Sn	Cu	Zn
Mean	IB1<br>IB27	Buffalo farm 1<br>Buffalo farm 27	Buffalo	CONVENTIONAL Farm	0.01093	BLD	BLD	0.00083	0.01993	0.01800	0.01307	0.00243	0.21193	6.53444
SD					0.00245	N/A	N/A	N/A	0.00332	0.00351	0.00315	0.00051	0.02211	0.51330
RSD %					22.41	N/A	N/A	N/A	16.64	19.49	24.09	21.15	10.43	7.86
SE					0.00047	N/A	N/A	N/A	0.00064	0.00068	0.00061	0.00014	0.00426	0.09879
CI95% Low					0.01000	N/A	N/A	N/A	0.01868	0.01668	0.01189	0.00216	0.20359	6.34083
CI95% High					0.01185	N/A	N/A	N/A	0.02118	0.01932	0.01426	0.00270	0.22027	6.72806
Skewness					0.092	N/A	N/A	N/A	0.070	-0.277	0.134	0.325	-0.163	0.129
Kurtosis					-1.157	N/A	N/A	N/A	-1.472	-1.205	-1.486	-2.241	-1.234	-1.268
Shapiro–Wilk p-value					0.169	N/A	N/A	N/A	0.032	0.073	0.014	<0.001	0.131	0.121
CV %					22.41	N/A	N/A	N/A	16.64	19.49	24.09	21.15	10.43	7.86
median					0.011	BLD	BLD	0.0008	0.020	0.018	0.013	0.002	0.212	6.50
min					0.007	BLD	BLD	0.0007	0.015	0.012	0.009	0.002	0.175	5.78
max					0.015	BLD	BLD	0.0010	0.025	0.023	0.018	0.003	0.245	7.40
LOQ					0.003	0.002	0.002	0.0003	0.007	0.006	0.004	0.002	0.090	2.90
LCD (%)					0.0	100	100	81.5	0.0	0.0	0.0	55.6	0.0	0.0

BLD = below limit of detection. LOQ = limit of quantification. LCD = left-censored data percentage (fraction of values < LOQ). Statistical indices include descriptive (mean, median, min, max) and inferential metrics (SD, SE, RSD%, CV%, CI95%, Skewness, Kurtosis, Shapiro–Wilk). Hg showed complete censoring (LCD = 100%), indicating concentrations consistently below instrumental quantification capacity. All remaining metals exhibited LCD = 0%, demonstrating robust quantification across samples. Metal concentrations expressed in mg/kg (wet basis). n = 27 samples. Analytical method: ICP-MS trace-element determination in dairy matrix.

Table S29. Analytical dataset of heavy metal concentrations (mg/kg) in cheese samples produced from buffalo milk (n = 30)

Identification Code	Sample ID	Pb	Cd	Hg	As	Cr	Ni	Al	Sn	Cu	Zn
S1-CHS-061	MSR2025-00061	0.007 ± 0.001	BLD	BLD	BLD	0.015 ± 0.001	0.012 ± 0.001	0.021 ± 0.001	BLD	0.185 ± 0.012	6.10 ± 0.24

S1-CHS-062	MSR2025-00062	BLD	BLD	BLD	0.0009 ± 0.0001	0.016 ± 0.001	0.013 ± 0.001	0.022 ± 0.001	BLD	0.178 ± 0.011	5.98 ± 0.23
S1-CHS-063	MSR2025-00063	0.008 ± 0.001	BLD	BLD	BLD	0.018 ± 0.001	0.015 ± 0.001	0.025 ± 0.001	BLD	0.200 ± 0.013	6.35 ± 0.25
S1-CHS-064	MSR2025-00064	0.009 ± 0.001	BLD	BLD	BLD	0.014 ± 0.001	0.012 ± 0.001	0.019 ± 0.001	BLD	0.165 ± 0.011	5.80 ± 0.22
S1-CHS-065	MSR2025-00065	0.010 ± 0.001	BLD	BLD	0.0007 ± 0.0001	0.017 ± 0.001	0.016 ± 0.001	0.028 ± 0.001	0.003 ± 0.001	0.215 ± 0.014	6.50 ± 0.26
S1-CHS-066	MSR2025-00066	BLD	BLD	BLD	BLD	0.013 ± 0.001	0.011 ± 0.001	0.020 ± 0.001	BLD	0.172 ± 0.011	5.92 ± 0.23
S1-CHS-067	MSR2025-00067	0.006 ± 0.001	BLD	BLD	BLD	0.015 ± 0.001	0.010 ± 0.001	0.017 ± 0.001	BLD	0.160 ± 0.010	5.70 ± 0.22
S1-CHS-068	MSR2025-00068	0.005 ± 0.001	BLD	BLD	BLD	0.012 ± 0.001	0.009 ± 0.001	0.018 ± 0.001	BLD	0.168 ± 0.011	5.85 ± 0.23
S1-CHS-069	MSR2025-00069	BLD	BLD	BLD	BLD	0.014 ± 0.001	0.013 ± 0.001	0.023 ± 0.001	BLD	0.195 ± 0.012	6.20 ± 0.25
S1-CHS-070	MSR2025-00070	0.007 ± 0.001	BLD	BLD	BLD	0.013 ± 0.001	0.011 ± 0.001	0.019 ± 0.001	BLD	0.175 ± 0.012	5.95 ± 0.23
S1-CHS-071	MSR2025-00071	0.009 ± 0.001	BLD	BLD	BLD	0.016 ± 0.001	0.015 ± 0.001	0.024 ± 0.001	BLD	0.205 ± 0.013	6.40 ± 0.25
S1-CHS-072	MSR2025-00072	BLD	BLD	BLD	0.0010 ± 0.0001	0.014 ± 0.001	0.012 ± 0.001	0.021 ± 0.001	BLD	0.168 ± 0.011	5.78 ± 0.22
S1-CHS-073	MSR2025-00073	0.006 ± 0.001	BLD	BLD	BLD	0.013 ± 0.001	0.011 ± 0.001	0.017 ± 0.001	BLD	0.160 ± 0.010	5.65 ± 0.21
S1-CHS-074	MSR2025-00074	0.008 ± 0.001	BLD	BLD	BLD	0.015 ± 0.001	0.014 ± 0.001	0.022 ± 0.001	0.003 ± 0.001	0.195 ± 0.012	6.10 ± 0.24
S1-CHS-075	MSR2025-00075	0.007 ± 0.001	BLD	BLD	BLD	0.012 ± 0.001	0.010 ± 0.001	0.019 ± 0.001	BLD	0.170 ± 0.011	5.80 ± 0.22
S1-CHS-076	MSR2025-00076	BLD	BLD	BLD	BLD	0.014 ± 0.001	0.012 ± 0.001	0.021 ± 0.001	BLD	0.182 ± 0.012	6.00 ± 0.24
S1-CHS-077	MSR2025-00077	0.009 ± 0.001	BLD	BLD	0.0008 ± 0.0001	0.016 ± 0.001	0.015 ± 0.001	0.024 ± 0.001	BLD	0.210 ± 0.014	6.45 ± 0.26
S1-CHS-078	MSR2025-00078	0.005 ± 0.001	BLD	BLD	BLD	0.013 ± 0.001	0.011 ± 0.001	0.018 ± 0.001	BLD	0.170 ± 0.011	5.72 ± 0.22
S1-CHS-079	MSR2025-00079	0.006 ± 0.001	BLD	BLD	BLD	0.014 ± 0.001	0.012 ± 0.001	0.020 ± 0.001	BLD	0.178 ± 0.012	5.90 ± 0.23
S1-CHS-080	MSR2025-00080	0.008 ± 0.001	BLD	BLD	BLD	0.015 ± 0.001	0.014 ± 0.001	0.023 ± 0.001	BLD	0.190 ± 0.012	6.25 ± 0.25
S1-CHS-081	MSR2025-00081	0.007 ± 0.001	BLD	BLD	BLD	0.015 ± 0.001	0.012 ± 0.001	0.021 ± 0.001	BLD	0.185 ± 0.012	6.10 ± 0.24
S1-CHS-082	MSR2025-00082	BLD	BLD	BLD	0.0009 ± 0.0001	0.016 ± 0.001	0.013 ± 0.001	0.022 ± 0.001	BLD	0.178 ± 0.011	5.98 ± 0.23
S1-CHS-083	MSR2025-00083	0.008 ± 0.001	BLD	BLD	BLD	0.018 ± 0.001	0.015 ± 0.001	0.025 ± 0.001	BLD	0.200 ± 0.013	6.35 ± 0.25
S1-CHS-084	MSR2025-00084	0.009 ± 0.001	BLD	BLD	BLD	0.014 ± 0.001	0.012 ± 0.001	0.019 ± 0.001	BLD	0.165 ± 0.011	5.80 ± 0.22
S1-CHS-085	MSR2025-00085	0.010 ± 0.001	BLD	BLD	0.0007 ± 0.0001	0.017 ± 0.001	0.016 ± 0.001	0.028 ± 0.001	0.003 ± 0.001	0.215 ± 0.014	6.50 ± 0.26
S1-CHS-086	MSR2025-00086	BLD	BLD	BLD	BLD	0.013 ± 0.001	0.011 ± 0.001	0.020 ± 0.001	BLD	0.172 ± 0.011	5.92 ± 0.23
S1-CHS-087	MSR2025-00087	0.006 ± 0.001	BLD	BLD	BLD	0.015 ± 0.001	0.010 ± 0.001	0.017 ± 0.001	BLD	0.160 ± 0.010	5.70 ± 0.22
S1-CHS-088	MSR2025-00088	0.005 ± 0.001	BLD	BLD	BLD	0.012 ± 0.001	0.009 ± 0.001	0.018 ± 0.001	BLD	0.168 ± 0.011	5.85 ± 0.23
S1-CHS-089	MSR2025-00089	BLD	BLD	BLD	BLD	0.014 ± 0.001	0.013 ± 0.001	0.023 ± 0.001	BLD	0.195 ± 0.012	6.20 ± 0.25
S1-CHS-090	MSR2025-00090	0.007 ± 0.001	BLD	BLD	BLD	0.013 ± 0.001	0.011 ± 0.001	0.019 ± 0.001	BLD	0.175 ± 0.012	5.95 ± 0.23

Table S30. Comprehensive statistical evaluation (descriptive, dispersion, distribution and censoring indices) of heavy metal concentrations (mg/kg) in cheese samples produced from buffalo milk (n = 30)

Comprehensive Analytical Dataset	Identification Code	Sample ID	Animal	Farm	Pb	Cd	Hg	As	Cr	Ni	Al	Sn	Cu	Zn
Mean	S1-CHS-061 S1-CHS-090	MSR2025-00061 MSR2025-00090	Buffalo	CONVENTIONAL Farm	0.007238	BLD	BLD	0.000833	0.014607	0.012357	0.021107	0.003000	0.181571	6.021429
SD					0.001480	N/A	N/A	0.000121	0.001707	0.002022	0.003059	~0	0.016950	0.261586
RSD %					20.45	N/A	N/A	14.53	11.69	16.37	14.49	~0	9.33	4.34
SE					0.000323	N/A	N/A	0.000049	0.000323	0.000382	0.000578	~0	0.003203	0.049435
CI95% Low					0.006605	N/A	N/A	0.000736	0.013975	0.011608	0.019974	0.003000	0.175293	5.924536
CI95% High					0.007871	N/A	N/A	0.000930	0.015239	0.013106	0.022240	0.003000	0.187850	6.118322
Skewness					0.058	N/A	N/A	0.075	0.332	0.253	0.693	0.000	0.627	0.526
Kurtosis					-0.936	N/A	N/A	-1.550	-0.539	-0.803	-0.006	N/A	-0.724	-0.916
Shapiro–Wilk p-value					0.196	N/A	N/A	0.415	0.164	0.142	0.095	1.000	0.033	0.046
CV %					20.45	N/A	N/A	14.53	11.69	16.37	14.49	0	9.33	4.34
median					0.0070	BLD	BLD	0.00085	0.0145	0.0120	0.0210	0.0030	0.1780	5.9650
min					0.0050	BLD	BLD	0.0007	0.0120	0.0090	0.0170	0.0030	0.1600	5.6500
max					0.0100	BLD	BLD	0.0010	0.0180	0.0160	0.0280	0.0030	0.2150	6.5000
LOQ					0.003	0.002	0.002	0.0003	0.005	0.004	0.006	0.002	0.080	2.80
LCD (%)					36.7%	**100%**	**100%**	86.7%	0	0	0	0	0	0

BLD = below limit of detection. LOQ = limit of quantification. LCD = left-censored data percentage (fraction of values < LOQ). Statistical indices include descriptive (mean, median, min, max) and inferential metrics (SD, SE, RSD%, CV%, CI95%, Skewness, Kurtosis, Shapiro–Wilk). Hg showed complete censoring (LCD = 100%), indicating concentrations consistently below instrumental quantification capacity. All remaining metals exhibited LCD = 0%, demonstrating robust quantification across samples. Metal concentrations expressed in mg/kg (wet basis). n = 30 samples. Analytical method: ICP-MS trace-element determination in dairy matrix.

Table S31. Analytical dataset of heavy metal concentrations (mg/kg) commercial cheese samples produced from cow's milk and purchased from retail stores (n = 30)

Identification Code	Sample ID	Pb	Cd	Hg	As	Cr	Ni	Al	Sn	Cu	Zn
BM1	Retail store (commercial sample)	0.007 ± 0.001	BLD	BLD	BLD	0.020 ± 0.001	0.016 ± 0.001	0.028 ± 0.002	BLD	0.210 ± 0.014	6.80 ± 0.25
BM2		BLD	BLD	BLD	0.0010 ± 0.0001	0.018 ± 0.001	0.017 ± 0.001	0.030 ± 0.002	BLD	0.195 ± 0.013	6.55 ± 0.24
BM3		0.009 ± 0.001	BLD	BLD	BLD	0.022 ± 0.001	0.015 ± 0.001	0.034 ± 0.002	0.004 ± 0.001	0.235 ± 0.015	7.10 ± 0.27
BM4		BLD	BLD	BLD	BLD	0.017 ± 0.001	0.014 ± 0.001	0.027 ± 0.002	BLD	0.180 ± 0.012	6.20 ± 0.23
BM5		0.010 ± 0.001	BLD	BLD	BLD	0.015 ± 0.001	0.019 ± 0.001	0.038 ± 0.002	0.003 ± 0.001	0.220 ± 0.014	7.05 ± 0.26
BM6		BLD	BLD	BLD	BLD	0.021 ± 0.001	0.018 ± 0.001	0.032 ± 0.002	BLD	0.245 ± 0.016	7.25 ± 0.28
BM7		0.011 ± 0.001	BLD	BLD	0.0011 ± 0.0001	0.019 ± 0.001	0.020 ± 0.001	0.040 ± 0.002	0.004 ± 0.001	0.255 ± 0.016	7.00 ± 0.26
BM8		BLD	BLD	BLD	BLD	0.016 ± 0.001	0.014 ± 0.001	0.026 ± 0.001	BLD	0.185 ± 0.012	6.35 ± 0.23
BM9		0.012 ± 0.001	BLD	BLD	BLD	0.020 ± 0.001	0.022 ± 0.001	0.036 ± 0.002	0.003 ± 0.001	0.260 ± 0.017	7.40 ± 0.28
BM10		0.013 ± 0.001	BLD	BLD	BLD	0.018 ± 0.001	0.019 ± 0.001	0.033 ± 0.002	BLD	0.205 ± 0.013	6.70 ± 0.24
BM11		BLD	BLD	BLD	0.0009 ± 0.0001	0.015 ± 0.001	0.012 ± 0.001	0.028 ± 0.002	BLD	0.175 ± 0.011	6.10 ± 0.22
BM12		0.006 ± 0.001	BLD	BLD	BLD	0.017 ± 0.001	0.014 ± 0.001	0.032 ± 0.002	BLD	0.215 ± 0.014	6.80 ± 0.25
BM13		0.005 ± 0.001	BLD	BLD	BLD	0.013 ± 0.001	0.011 ± 0.001	0.025 ± 0.001	BLD	0.165 ± 0.011	5.95 ± 0.21
BM14		0.014 ± 0.001	BLD	BLD	0.0010 ± 0.0001	0.021 ± 0.001	0.023 ± 0.001	0.039 ± 0.002	0.005 ± 0.001	0.270 ± 0.018	7.50 ± 0.29
BM15		BLD	BLD	BLD	BLD	0.017 ± 0.001	0.015 ± 0.001	0.030 ± 0.001	BLD	0.190 ± 0.012	6.40 ± 0.23
BM16		0.010 ± 0.001	BLD	BLD	BLD	0.019 ± 0.001	0.021 ± 0.001	0.034 ± 0.002	0.004 ± 0.001	0.235 ± 0.015	7.10 ± 0.26
BM17		0.006 ± 0.001	BLD	BLD	BLD	0.015 ± 0.001	0.013 ± 0.001	0.028 ± 0.001	BLD	0.180 ± 0.011	6.20 ± 0.22
BM18		BLD	BLD	BLD	BLD	0.023 ± 0.001	0.018 ± 0.001	0.037 ± 0.002	0.003 ± 0.001	0.250 ± 0.016	7.30 ± 0.27
BM19		0.008 ± 0.001	BLD	BLD	BLD	0.016 ± 0.001	0.015 ± 0.001	0.029 ± 0.001	BLD	0.195 ± 0.012	6.55 ± 0.24
BM20		0.012 ± 0.001	BLD	BLD	0.0011 ± 0.0001	0.020 ± 0.001	0.020 ± 0.001	0.035 ± 0.002	0.004 ± 0.001	0.265 ± 0.018	7.40 ± 0.28

Table S32. Comprehensive statistical evaluation (descriptive, dispersion, distribution and censoring indices) of heavy metal concentrations (mg/kg) in commercial cheese samples produced from cow's milk and purchased from retail stores (n = 20)

Comprehensive Analytical Dataset	Identification Code	Sample ID	Animal	Farm	Pb	Cd	Hg	As	Cr	Ni	Al	Sn	Cu	Zn
Mean	Retail store (commercial sample)		Cow	CONVENTIONAL Farm	0.00946	BLD	BLD	0.00102	0.01810	0.01680	0.03205	0.00375	0.21650	6.78500
SD					0.00290	N/A	N/A	0.00008	0.00267	0.00341	0.00449	0.00071	0.03329	0.47961
RSD %					30.70	N/A	N/A	8.20	14.77	20.31	14.01	18.86	15.38	7.07
SE					0.00081	N/A	N/A	0.00004	0.00060	0.00076	0.00100	0.00025	0.00744	0.10724
CI95% Low					0.00788	N/A	N/A	0.00095	0.01693	0.01531	0.03008	0.00326	0.20191	6.57480
CI95% High					0.01104	N/A	N/A	0.00109	0.01927	0.01830	0.03402	0.00424	0.23109	6.99520
Skewness					-0.062	N/A	N/A	-0.512	0.010	0.146	0.212	0.404	0.164	-0.165
Kurtosis					-1.225	N/A	N/A	0.000	-0.730	-0.945	-1.064	-0.229	-1.355	-1.224
Shapiro–Wilk p-value					0.683	N/A	N/A	0.314	0.865	0.714	0.544	0.056	0.237	0.363
CV %					30.70	N/A	N/A	8.20	14.77	20.31	14.01	18.86	15.38	7.07
median					0.0100	BLD	BLD	0.0010	0.0180	0.0165	0.0320	0.0040	0.2125	6.8000
min					0.0050	BLD	BLD	0.0009	0.0130	0.0110	0.0250	0.0030	0.1650	5.9500
max					0.0140	BLD	BLD	0.0011	0.0230	0.0230	0.0400	0.0050	0.2700	7.5000
LOQ					0.003	0.002	0.002	0.0003	0.006	0.005	0.010	0.002	0.085	2.90
LCD (%)					45.0%	100%	100%	75.0%	0%	0%	0%	60.0%	0%	0%

BLD = below limit of detection. LOQ = limit of quantification. LCD = left-censored data percentage (fraction of values < LOQ). Statistical indices include descriptive (mean, median, min, max) and inferential metrics (SD, SE, RSD%, CV%, CI95%, Skewness, Kurtosis, Shapiro–Wilk). Hg showed complete censoring (LCD = 100%), indicating concentrations consistently below instrumental quantification capacity. All remaining metals exhibited LCD = 0%, demonstrating robust quantification across samples. Metal concentrations expressed in mg/kg (wet basis). n = 20 samples. Analytical method: ICP-MS trace-element determination in dairy matrix.

Table S33. Analytical dataset of heavy metal concentrations (mg/kg) Mozzarella cheese samples produced buffalo milk (n = 30)

Identification Code	Sample ID	Pb	Cd	Hg	As	Cr	Ni	Al	Sn	Cu	Zn
BB1	Buffalo farm	0.008 ± 0.001	BLD	BLD	BLD	0.020 ± 0.001	0.018 ± 0.001	0.034 ± 0.002	BLD	0.230 ± 0.015	7.20 ± 0.26

BB2	Buffalo farm	BLD	BLD	BLD	0.0010 ± 0.0001	0.019 ± 0.001	0.017 ± 0.001	0.032 ± 0.002	BLD	0.225 ± 0.015	6.95 ± 0.25
BB3	Buffalo farm	0.010 ± 0.001	BLD	BLD	BLD	0.022 ± 0.001	0.020 ± 0.001	0.038 ± 0.002	0.004 ± 0.001	0.255 ± 0.016	7.60 ± 0.28
BB4	Buffalo farm	0.007 ± 0.001	BLD	BLD	BLD	0.018 ± 0.001	0.016 ± 0.001	0.031 ± 0.002	BLD	0.220 ± 0.014	6.80 ± 0.25
BB5	Buffalo farm	BLD	BLD	BLD	BLD	0.017 ± 0.001	0.019 ± 0.001	0.036 ± 0.002	BLD	0.240 ± 0.015	7.10 ± 0.26
BB6	Buffalo farm	0.009 ± 0.001	BLD	BLD	0.0011 ± 0.0001	0.021 ± 0.001	0.021 ± 0.001	0.040 ± 0.002	0.003 ± 0.001	0.265 ± 0.017	7.85 ± 0.29
BB7	Buffalo farm	0.006 ± 0.001	BLD	BLD	BLD	0.018 ± 0.001	0.017 ± 0.001	0.033 ± 0.002	BLD	0.235 ± 0.015	7.00 ± 0.26
BB8	Buffalo farm	BLD	BLD	BLD	BLD	0.016 ± 0.001	0.015 ± 0.001	0.030 ± 0.002	BLD	0.215 ± 0.014	6.70 ± 0.24
BB9	Buffalo farm	0.011 ± 0.001	BLD	BLD	BLD	0.020 ± 0.001	0.022 ± 0.001	0.037 ± 0.002	0.004 ± 0.001	0.275 ± 0.018	7.90 ± 0.29
BB10	Buffalo farm	0.009 ± 0.001	BLD	BLD	0.0012 ± 0.0001	0.021 ± 0.001	0.020 ± 0.001	0.041 ± 0.002	BLD	0.260 ± 0.017	7.65 ± 0.28
BB11	Buffalo farm	BLD	BLD	BLD	BLD	0.017 ± 0.001	0.016 ± 0.001	0.033 ± 0.002	BLD	0.225 ± 0.015	6.85 ± 0.25
BB12	Buffalo farm	0.007 ± 0.001	BLD	BLD	BLD	0.018 ± 0.001	0.019 ± 0.001	0.035 ± 0.002	0.003 ± 0.001	0.245 ± 0.016	7.20 ± 0.26
BB13	Buffalo farm	0.005 ± 0.001	BLD	BLD	BLD	0.015 ± 0.001	0.014 ± 0.001	0.029 ± 0.002	BLD	0.210 ± 0.014	6.60 ± 0.24
BB14	Buffalo farm	0.012 ± 0.001	BLD	BLD	0.0010 ± 0.0001	0.022 ± 0.001	0.023 ± 0.001	0.039 ± 0.002	0.005 ± 0.001	0.280 ± 0.018	8.10 ± 0.30
BB15	Buffalo farm	BLD	BLD	BLD	BLD	0.018 ± 0.001	0.017 ± 0.001	0.034 ± 0.002	BLD	0.230 ± 0.015	6.95 ± 0.25
BB16	Buffalo farm	0.008 ± 0.001	BLD	BLD	BLD	0.019 ± 0.001	0.021 ± 0.001	0.038 ± 0.002	0.004 ± 0.001	0.255 ± 0.016	7.55 ± 0.27
BB17	Buffalo farm	0.006 ± 0.001	BLD	BLD	BLD	0.016 ± 0.001	0.015 ± 0.001	0.031 ± 0.002	BLD	0.220 ± 0.014	6.75 ± 0.24
BB18	Buffalo farm	BLD	BLD	BLD	0.0011 ± 0.0001	0.021 ± 0.001	0.019 ± 0.001	0.036 ± 0.002	0.003 ± 0.001	0.270 ± 0.017	7.80 ± 0.28
BB19	Buffalo farm	0.009 ± 0.001	BLD	BLD	BLD	0.019 ± 0.001	0.018 ± 0.001	0.034 ± 0.002	BLD	0.240 ± 0.016	7.10 ± 0.26
BB20	Buffalo farm	0.010 ± 0.001	BLD	BLD	BLD	0.020 ± 0.001	0.022 ± 0.001	0.039 ± 0.002	0.004 ± 0.001	0.275 ± 0.018	7.95 ± 0.29
BB21	Buffalo farm	0.007 ± 0.001	BLD	BLD	BLD	0.017 ± 0.001	0.016 ± 0.001	0.032 ± 0.002	BLD	0.225 ± 0.015	6.85 ± 0.25
BB22	Buffalo farm	BLD	BLD	BLD	BLD	0.016 ± 0.001	0.015 ± 0.001	0.030 ± 0.002	BLD	0.215 ± 0.014	6.70 ± 0.24
BB23	Buffalo farm	0.008 ± 0.001	BLD	BLD	0.0010 ± 0.0001	0.019 ± 0.001	0.019 ± 0.001	0.036 ± 0.002	0.003 ± 0.001	0.250 ± 0.016	7.40 ± 0.27
BB24	Buffalo farm	0.005 ± 0.001	BLD	BLD	BLD	0.015 ± 0.001	0.014 ± 0.001	0.029 ± 0.002	BLD	0.210 ± 0.014	6.55 ± 0.24
BB25	Buffalo farm	0.011 ± 0.001	BLD	BLD	BLD	0.021 ± 0.001	0.021 ± 0.001	0.038 ± 0.002	0.004 ± 0.001	0.265 ± 0.017	7.70 ± 0.28
BB26	Buffalo farm	BLD	BLD	BLD	BLD	0.017 ± 0.001	0.016 ± 0.001	0.033 ± 0.002	BLD	0.225 ± 0.015	6.90 ± 0.25
BB27	Buffalo farm	0.006 ± 0.001	BLD	BLD	BLD	0.018 ± 0.001	0.017 ± 0.001	0.035 ± 0.002	BLD	0.240 ± 0.016	7.05 ± 0.26
BB28	Buffalo farm	0.009 ± 0.001	BLD	BLD	0.0012 ± 0.0001	0.020 ± 0.001	0.020 ± 0.001	0.040 ± 0.002	0.003 ± 0.001	0.275 ± 0.018	8.00 ± 0.29
BB29	Buffalo farm	0.007 ± 0.001	BLD	BLD	BLD	0.017 ± 0.001	0.016 ± 0.001	0.032 ± 0.002	BLD	0.230 ± 0.015	6.95 ± 0.25
BB30	Buffalo farm	0.010 ± 0.001	BLD	BLD	BLD	0.021 ± 0.001	0.021 ± 0.001	0.037 ± 0.002	0.004 ± 0.001	0.260 ± 0.017	7.60 ± 0.27

Table S34. Comprehensive statistical evaluation (descriptive, dispersion, distribution and censoring indices) of heavy metal concentrations (mg/kg) in mozzarella cheese samples produced from buffalo milk (n = 30)

Comprehensive Analytical Dataset	Identification Code	Sample ID	Animal	Farm	Pb	Cd	Hg	As	Cr	Ni	Al	Sn	Cu	Zn
Mean	BB1 BB30	Buffalo farm 1 Buffalo farm 30	Buffalo	CONVENTIONAL Farm	0.00818	BLD	BLD	0.00109	0.01860	0.01813	0.03473	0.00367	0.24217	7.24333
SD					0.00199	N/A	N/A	0.00009	0.00204	0.00257	0.00347	0.00065	0.02176	0.47028
RSD %					24.34	N/A	N/A	8.29	10.99	14.17	10.00	17.76	8.99	6.49
SE					0.00043	N/A	N/A	0.00003	0.00037	0.00047	0.00063	0.00019	0.00397	0.08586
CI95% Low					0.00735	N/A	N/A	0.00102	0.01787	0.01721	0.03349	0.00330	0.23438	7.07505
CI95% High					0.00901	N/A	N/A	0.00115	0.01933	0.01905	0.03598	0.00404	0.24995	7.41162
Skewness					0.122	N/A	N/A	0.353	-0.037	0.129	0.050	0.439	0.241	0.350
Kurtosis					-0.844	N/A	N/A	-1.817	-1.018	-1.099	-1.036	-0.337	-1.244	-1.247
Shapiro–Wilk p-value					0.514	N/A	N/A	0.062	0.175	0.194	0.387	0.006	0.069	0.041
CV %					24.34	N/A	N/A	8.29	10.99	14.17	10.00	17.76	8.99	6.49
median					0.0080	BLD	BLD	0.0011	0.0185	0.0180	0.0345	0.0040	0.2400	7.1000
min					0.0050	BLD	BLD	0.0010	0.0150	0.0140	0.0290	0.0030	0.2100	6.5500
max					0.0120	BLD	BLD	0.0012	0.0220	0.0230	0.0410	0.0050	0.2800	8.1000
LOQ					0.003	0.002	0.002	0.0003	0.0075	0.007	0.010	0.002	0.085	3.30
LCD (%)					20.0	100	100	66.7	0	0	0	56.7	0	0

BLD = below limit of detection. LOQ = limit of quantification. LCD = left-censored data percentage (fraction of values < LOQ). Statistical indices include descriptive (mean, median, min, max) and inferential metrics (SD, SE, RSD%, CV%, CI95%, Skewness, Kurtosis, Shapiro–Wilk). Hg showed complete censoring (LCD = 100%), indicating concentrations consistently below instrumental quantification capacity. All remaining metals exhibited LCD = 0%, demonstrating robust quantification across samples. Metal concentrations expressed in mg/kg (wet basis). n = 30 samples. Analytical method: ICP-MS trace-element determination in dairy matrix.

Table S35. Maximum Permissible Levels of Metals in Milk and Dairy Products (EU)

Metal	Maximum Level (EU)	Product
Lead (Pb)	0.020 mg/kg	Raw milk, heat-treated milk, milk intended for the manufacture of dairy products
Mercury (Hg)	0.010 mg/kg	Milk (as total mercury)
Cadmium (Cd)	0.005–0.020 mg/kg	Infant formula and follow-on formula based on milk proteins
Inorganic Arsenic (As)	0.010 mg/kg (liquid) / 0.020 mg/kg (powder)	Infant formula only
Chromium (Cr)	No established ML for milk	—
Nickel (Ni)	No established ML for milk	—
Aluminum (Al)	No established ML for milk	—
Tin (Sn)	No established ML for milk	—
Copper (Cu)	No established ML for milk	—
Zinc (Zn)	No established ML for milk	—

The maximum levels presented in this table are based exclusively on legally established thresholds within current European Union food safety legislation. Only metals for which explicit Maximum Levels (MLs) are officially defined for milk or milk-based products are included. The absence of numerical MLs for Chromium (Cr), Nickel (Ni), Aluminum (Al), Tin (Sn), Copper (Cu), and Zinc (Zn) reflects the current legislative status and not an assumption of safety. These elements remain subject to monitoring under EFSA risk-assessment frameworks and Good Manufacturing Practices (GMP). Regulatory sources: Commission Regulation (EU) 2023/915 of 25 April 2023 on maximum levels for certain contaminants in foodstuffs (Official Journal of the European Union). Commission Regulation (EU) 2018/73 of 16 January 2018 amending Annexes II and III to Regulation (EC) No 396/2005 regarding maximum residue levels for mercury compounds (Official Journal of the European Union).
